# Strengthening primary care for diabetes and hypertension in Eswatini: study protocol for a nationwide cluster-randomized controlled trial

**DOI:** 10.1186/s13063-023-07096-4

**Published:** 2023-03-22

**Authors:** Michaela Theilmann, Ntombifuthi Ginindza, John Myeni, Sijabulile Dlamini, Bongekile Thobekile Cindzi, Dumezweni Dlamini, Thobile L. Dlamini, Maike Greve, Harsh Vivek Harkare, Mbuso Hleta, Philile Khumalo, Lutz M. Kolbe, Simon Lewin, Lisa-Rufaro Marowa, Sakhile Masuku, Dumsile Mavuso, Marjan Molemans, Nyasatu Ntshalintshali, Nomathemba Nxumalo, Brianna Osetinsky, Christopher Pell, Ria Reis, Fortunate Shabalala, Bongumusa R. Simelane, Lisa Stehr, Fabrizio Tediosi, Frank van Leth, Jan-Walter De Neve, Till Bärnighausen, Pascal Geldsetzer

**Affiliations:** 1 Heidelberg Institute of Global Health, Heidelberg University, Im Neuenheimer Feld 130.3, 69120 Heidelberg, Germany; 2 Assistant Professorship of Behavioral Science for Disease Prevention and Health Care, Technical University of Munich, Munich, Germany; 3 Ministry of Health in Eswatini, Ministry of Justice & Constitutional Affairs Building, Mhlambanyatsi Road, Mbabane, Eswatini; 4 Clinton Health Access Initiative, Mbhilibhi House, Plot 170, Corner Tsekwane/Mbhilibhi Street, Mbabane, Eswatini; 5 Diabetes Eswatini, Manzini, Eswatini; 6 Eswatini Business Health and Wellness, Malagwane Hill, Mbabane, Eswatini; 7 University of Göttingen, Humboldtallee 3, 37073 Göttingen, Germany; 8 Swiss Tropical and Public Health Institute, Kreuzstrasse 2, 4123 Allschwil, Switzerland; 9 University of Basel, Basel, Switzerland; 10 Division of Health Services, Norwegian Institute of Public Health, Oslo, Norway; 11 Department of Health Sciences Ålesund, Norwegian University of Science and Technology (NTNU), Ålesund, Norway; 12 Health Systems Research Unit, South African Medical Research Council, Cape Town, South Africa; 13 University of Eswatini, M48V+JXG, Mbabane, Eswatini; 14 Amsterdam Institute for Global Health and Development, Paasheuvelweg 25, 1105 BP Amsterdam, Netherlands; 15 Amsterdam Institute for Social Science Research, Amsterdam, Netherlands; 16 Amsterdam Public Health Research Institute, Amsterdam, Netherlands; 17 Amsterdam UMC, location University of Amsterdam, Department of Global Health, Amsterdam, The Netherlands; 18 Dept. of Public Health & Primary Care, Leiden University Medical Center, Albinusdreef 2, 2333 ZA Leiden, The Netherlands; 19 School of Child and Adolescent Health, Children’s Institute, University of Cape Town, Cape Town, South Africa; 20 Department of Health Sciences, Vrije Universiteit, De Boelelaan 1105, 1081 HV Amsterdam, The Netherlands; 21 Harvard Center for Population and Development Studies, Cambridge, USA; 22 Africa Health Research Institute, KwaZulu-Natal, 3935 South Africa; 23 Division of Primary Care and Population Health, Stanford University, 291 Campus Drive, Stanford, CA 94305-5101 USA; 24 Chan Zuckerberg Biohub, San Francisco, CA 94158 USA

**Keywords:** Diabetes, Hypertension, WHO-PEN, Health service decentralization, Community health worker, Differentiated service delivery, Community outreach, Eswatini, Cardiovascular disease, Non-communicable disease

## Abstract

**Background:**

Diabetes and hypertension are increasingly important population health challenges in Eswatini. Prior to this project, healthcare for these conditions was primarily provided through physician-led teams at tertiary care facilities and accessed by only a small fraction of people living with diabetes or hypertension. This trial tests and evaluates two community-based healthcare service models implemented at the national level, which involve health care personnel at primary care facilities and utilize the country’s public sector community health worker cadre (the rural health motivators [RHMs]) to help generate demand for care.

**Methods:**

This study is a cluster-randomized controlled trial with two treatment arms and one control arm. The unit of randomization is a primary healthcare facility along with all RHMs (and their corresponding service areas) assigned to the facility. A total of 84 primary healthcare facilities were randomized in a 1:1:1 ratio to the three study arms. The first treatment arm implements differentiated service delivery (DSD) models at the clinic and community levels with the objective of improving treatment uptake and adherence among clients with diabetes or hypertension. In the second treatment arm, community distribution points (CDPs), which previously targeted clients living with human immunodeficiency virus, extend their services to clients with diabetes or hypertension by allowing them to pick up medications and obtain routine nurse-led follow-up visits in their community rather than at the healthcare facility. In both treatment arms, RHMs visit households regularly, screen clients at risk, provide personalized counseling, and refer clients to either primary care clinics or the nearest CDP. In the control arm, primary care clinics provide diabetes and hypertension care services but without the involvement of RHMs and the implementation of DSD models or CDPs. The primary endpoints are mean glycated hemoglobin (HbA1c) and systolic blood pressure among adults aged 40 years and older living with diabetes or hypertension, respectively. These endpoints will be assessed through a household survey in the RHM service areas. In addition to the health impact evaluation, we will conduct studies on cost-effectiveness, syndemics, and the intervention’s implementation processes.

**Discussion:**

This study has the ambition to assist the Eswatini government in selecting the most effective delivery model for diabetes and hypertension care. The evidence generated with this national-level cluster-randomized controlled trial may also prove useful to policy makers in the wider Sub-Saharan African region.

**Trial registration:**

NCT04183413. Trial registration date: December 3, 2019

**Supplementary Information:**

The online version contains supplementary material available at 10.1186/s13063-023-07096-4.

## Background

### Diabetes and hypertension in Eswatini

Diabetes and hypertension are a rapidly growing public health problem in the Kingdom of Eswatini. Approximately a quarter of the population aged 15–69 have hypertension and almost one fifth live with diabetes or prediabetes [1]. Globally, diabetes and hypertension are among the leading drivers of disability, being responsible for the majority of strokes, kidney failure, blindness, and lower limb amputation [2]. Population aging will likely further increase these numbers over the coming years [3].

Before the start of this study in 2019, the Eswatini Ministry of Health identified three cross-cutting barriers that prevented people living with diabetes or hypertension from benefiting from prompt, sustained access to appropriate healthcare. First, there was a low detection rate because at-risk clients were not systematically screened for diabetes and hypertension. Eighty to ninety percent of people living with diabetes or hypertension were not aware of having these conditions [1]. Second, access to care for diabetes and hypertension was poor. All diabetes and hypertension care was provided by physician-led teams in 13 tertiary care facilities, which were concentrated in urban areas. Less than 10% of the population lived within 2 h of travel time to the nearest public hospital [4]. Third, the lack of standardized treatment guidelines for diabetes and hypertension meant that the quality of disease management was often low and varied significantly between facilities and regions.

### Study objectives

WHO-PEN@Scale is a nationwide cluster-randomized controlled trial with the objective of comprehensively evaluating two diabetes and hypertension health service provision models at the community and household levels. First, we will estimate the causal impact of these models on diabetes and hypertension control at the population level. Second, comprehensive implementation and process evaluations will identify implementation challenges and enablers, including the acceptability of the interventions among healthcare workers. Third, we will endeavor to identify syndemic processes involving diabetes, hypertension, and other diseases such as human immunodeficiency virus (HIV). Fourth, we will conduct detailed cost analyses both from the the government and client perspective. While all these research components may yield valuable results on their own, we believe that their interaction and complementarity will allow us to draw a comprehensive and overarching picture of the government’s health care reform. Here we detail the study protocol for the first and third objectives (impact assessment and syndemics analysis), whereas study protocols for the other objectives (implementation and acceptability studies, cost analysis) will be reported elsewhere.

## Methods / design

**Fig. 1:**
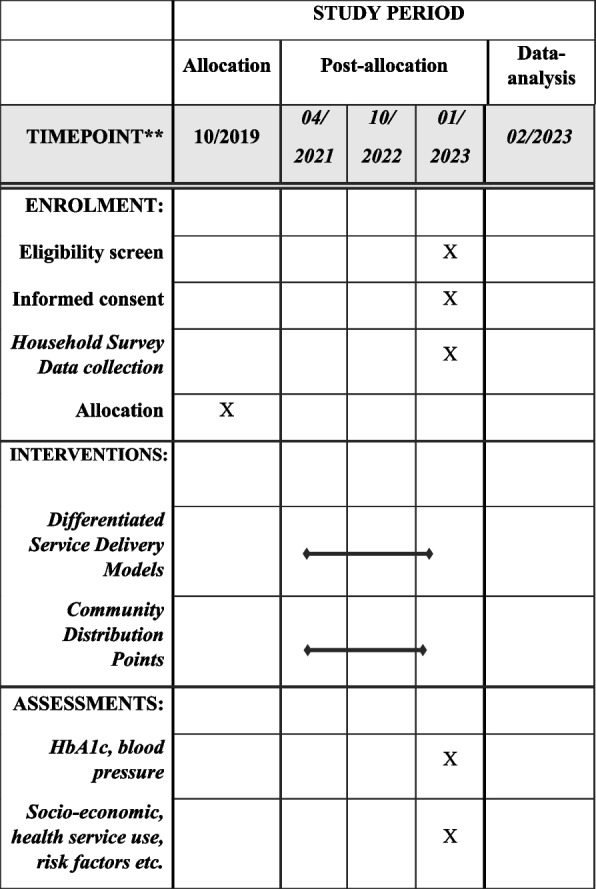
SPIRIT + figure. Example template of recommended content for the schedule of enrolment, interventions, and assessments*

We used the SPIRIT reporting guidelines in the development of this study protocol (Fig. 1) [5].

### Study setting

The Kingdom of Eswatini is a country in Southern Africa with a population of approximately 1.2 million [6]. Although only 11% of the adult population aged 45 to 69 years reported currently smoking, and 18% having drunk alcohol in the past 30 days, over 60% of adults were overweight or obese in 2014 [1]. Over one in two adults in this age range had raised blood pressure or took hypertension medication and among these less than 10% had achieved control of their blood pressure (defined as a systolic blood pressure less than 140 mmHg and diastolic blood pressure less than 90 mmHg). Among all adults aged 15 to 69 years with hypertension, 68% reported to have ever had their blood pressure measured, 37% to have ever been diagnosed with hypertension, 21% to be taking hypertension medication, and 7% had a controlled blood pressure [7]. Almost a tenth of adults aged 45 to 69 years had impaired fasting glycemia and one in four adults had raised blood glucose [1]. Eswatini has an HIV prevalence of 28% among adults aged 15–49, which is one of the highest prevalence estimates in the world [8]. In 2019, the Eswatini government allocated 7% of its budget to health care expenditure [9].

These figures illustrate the vast unmet need for diabetes and hypertension care, which, at the start of this project, was primarily provided by physicians in hospitals (Table 1). To successfully address the high health burden associated with diabetes and hypertension, lower levels of care, such as nurses in primary care clinics and community health workers in communities, could represent a highly valuable resource.

**Table 1:** Health system data

**Population**		1,160,000
**Health facilities**	*Tertiary care level*	
	Hospitals	4
	National Referral Hospitals	3
	Regional Hospital	7
	Public Health Units	8
	*Primary care level*	
	Primary care clinic (public)	118
**Health workforce**		
	Physicians	126
	Nurses	1505
	Community health workers (Rural Health Motivators)	> 3000

Sources: Eswatini Ministry of Health: Human resources for health strategic plan (2019-2023), unpublished and UN population estimates [6]

### WHO-PEN

The World Health Organization (WHO) Package of Essential Non-Communicable Disease (NCD) Interventions (WHO-PEN) is a collection of cost-effective interventions at the primary care level in low-resource settings. It includes guidelines on the screening, diagnosis, and treatment (medical as well as lifestyle counseling) of several NCDs including diabetes and hypertension. The goal of WHO-PEN is to address the unmet need for high-quality NCD care in low- and middle-income countries (LMICs) by increasing service coverage. In the case of our study, we aim to improve access to care through the extension of services offered as well as shifting care from tertiary facilities to health personnel at primary care clinics. Eswatini’s public sector cadre of community health workers, who regularly visit households, could further support individuals in obtaining and adhering to care. The WHO-PEN@Scale project aims to evaluate the scale-up of these changes in health service provision.

### Study arms

1. Standard of care

WHO-PEN@Scale consists of two treatment arms and one control arm. The standard of care (SoC) arm is the control arm and consists of diabetes and hypertension care as it is currently provided across the country. Until 2020, care for uncomplicated and complicated cases was primarily provided at tertiary care facilities by physician-led teams. There was no systematic screening of at-risk clients, but the screening was mainly conducted when clients presented at hospitals with other severe conditions or during antenatal care at primary care facilities. Treatment for diabetes and hypertension was exclusively initiated at tertiary care facilities, and drug refills were also mainly obtained there. No standardized treatment nor lifestyle counseling guidelines for diabetes and hypertension care were available.

At the end of 2019, the Eswatini Ministry of Health started the decentralization of health services according to the WHO-PEN guidelines in the clinics that we randomly allocated to the two treatment arms. Health care personnel at primary care facilities were trained on the screening and treatment of diabetes and hypertension and facilities were equipped with blood glucose and digital blood pressure measurement devices. Nurses started providing health services for diabetes and hypertension according to the developed Standard Operating Procedures guidelines (SOPs, see below for more details). When the COVID-19 pandemic began in early 2020, the Eswatini government initiated an “emergency decentralization” to decongest tertiary care facilities and to protect people living with diabetes and hypertension, who are at high risk of severe outcomes from COVID-19 [10]. The emergency decentralization encompassed a nationwide scale-up of the WHO-PEN intervention originally envisioned to be implemented only in the two treatment arms of the WHO-PEN@Scale project. As such, care for diabetes and hypertension was shifted from physicians in tertiary facilities to nurses in all public primary care clinics across the country in April 2020. Thus, what had initially been planned to be the health service decentralization to be evaluated under the WHO-PEN@Scale study, became the new standard of care.

In all public primary care clinics across Eswatini, nurses were trained on systematic screening, treatment initiation, lifestyle counseling, and follow-up guided by the SOPs (Eswatini Ministry of Health: Clinic level non-communicable diseases case management desk guide, unpublished). Nurses were instructed to screen all clients for diabetes and hypertension if they have a past or family history of cardiovascular disease, diabetes, or hypertension; are aged 40 years or older; or have risk factors such as overweight, tobacco use, lack of exercise, or an unhealthy diet. Uncomplicated cases are to be initiated, treated, and followed up by the nurses in the primary care clinics whereas complicated cases (“complex clients”, see Table 2 for the definition) are referred to tertiary care facilities.

**Table 2:** Definition of complex clients

**Hypertension**^a^	**Diabetes**^a^
Age < 40	Age < 40
Severe hypertension (≥ 180/110 mmHg)	Severe hypertension (≥ 180/110 mmHg)
Multiple severe risk factors	Multiple severe risk factors
Multiple comorbidities	Multiple comorbidities
Elderly clients	Elderly clients
Pregnant women	Pregnant women
Symptoms of cardiovascular disease	Symptoms of cardiovascular disease
Kidney disease	Kidney disease
Visual problems (retinopathy)	Visual problems (retinopathy)
Suspected type 1 diabetes	Suspected type 1 diabetes
Suspected secondary hypertension	Suspected secondary hypertension
	Recurrent hypoglycemia
	Diabetic ketoacidosis
	Hyperosmolar hyperglycemic state

^a^A client is classified as “complex” if any one of the criteria listed in the table applies

Diabetes and hypertension care follow a stepwise standardized procedure that guides nurses through care depending on the client’s disease status (Tables 3 and 4). Prescriptions are given to the client on a monthly basis. The SOPs also include a detailed step-by-step guide on how to structure counseling sessions, behavioral risk factor modification advice messages, and details on drugs and their correct dose. These SOPs were developed during a successful decentralization pilot in the Lubombo region (one out of four regions in Eswatini), conducted between 2014 and 2016, and adapted for the WHO-PEN@Scale project based on the pilot’s results [11].

(2) Treatment arm 1: Differentiated service delivery models

**Table 3:** Stepwise management of hypertension

Entry point	Management
**Step 1: Community clinic level**	
Mild hypertension:	Lifestyle modification if client is committed
BP 140–159/90–99 mmHg	
	If not controlled after 1–3 months, go to step 2
	If multiple risk factors present, go to step 2
	For complex clients, refer to physician-led service
**Step 2: Community clinic level**	
Failure at step 1 OR	Lifestyle modification
Moderate hypertension:	+
	Hydrochlorothiazide^a^ 12.5 to 25 mg daily until target BP reached
BP 160–179/100–109 mmHg	
**Step 3: Initiation at hospital level. Follow up at clinic level when stable**	
Failure at step 2 OR	Lifestyle modification
Severe hypertension:^b^	+
	Hydrochlorothiazide 25 mg daily
BP ≥ 180/110 mmHg	+
	ACE inhibitor (e.g., Captopril) OR
	Calcium channel blocker (e.g., Nifedipine SR)
**Step 4: Initiation at hospital level. Follow up at clinic level when stable**	
Failure at step 3	Lifestyle modification
	+
	Hydrochlorothiazide 25 mg daily
	+
	ACE inhibitor (e.g., Captopril)
	+
	Calcium channel blocker (e.g., Nifedipine SR)
**Step 5: Hospital level**	
Failure at step 4	Clients who have failed at step 4 should be managed by physician-led services and managed according to the physician’s best judgment

*Abbreviations*: *BP* blood pressure

^*a^Hydrochlorothiazide can cause elevated blood sugar. When initiating in diabetic or pre-diabetic clients, monitor blood glucose at least twice per week for the first 2 weeks

^**b^For clients not already on treatment, if BP ≥ 180/110, give a stat dose of Captopril 12.5 mg by the nurse before referring to the doctor-led service immediately

**Table 4:** Stepwise management of type 2 diabetes

Entry point	Management
**Step 1: Community clinic level**	
Prediabetes:	Inform client they may develop diabetes in future
FBG 5.6–6.9 mmol/l	Advise lifestyle modification
RBG 7–11 mmol/l	
	Check blood glucose every 6 months
**Step 2: Community clinic level**	
Diabetes:	Lifestyle modification if client is committed
FBG 7–10 mmol/l	If not controlled after 1–3 months go to step 3
RBG ≥ 11.1–17 mmol/l	
	If FBG > 10 mmol/l or RBG > 17 go to step 3
	For complex clients refer to doctor-led service
**Step 3: Initiation at hospital level. Follow up at clinic level when stable**	
Failure at step 2 OR	Lifestyle modification
	+
FBG > 10 mmol/l	Metformin
RBG > 17 mmol/l	
**Step 4: Initiation at hospital level. Follow up at clinic level when stable**	
Failure at step 3	Lifestyle modification
	+
	Metformin
	+
	Sulphonylurea
**Step 5: Hospital level**	
Failure at step 4	Consider insulin
	Follow up at hospital

*Abbreviations*: *FBG* fasting blood glucose, *RBG* random blood glucose

The first treatment arm, the differentiated service delivery (DSD) arm, adapts models developed initially for HIV care. The objective of the DSD models is to improve access and retention to HIV care and reduce the burden placed on the health system [12]. In Eswatini, these models have been implemented since 2016 and are viewed by the government as crucial in the HIV response [13]. In the DSD arm, three mutually exclusive models are introduced for diabetes and hypertension care: (i) the facility-based fast-track model, (ii) the facility-based treatment club model, and (iii) the community-level advisory groups. The eligibility criteria for the models are detailed in Table 5. Clients can choose whether they want to be enrolled in a DSD model and if so, in which.

**Table 5:** Eligibility criteria for differentiated service delivery models

**Age**	≥ 18 years
Months on medication	≥ 12 months
Blood pressure/blood glucose	Blood pressure < 140/90 mmHg and/or HbA1c < 6.5% or fasting blood glucose < 7.0 mmol/l
Prior clinic visits for diabetes/hypertension care	≥ Two clinic visits for diabetes/hypertension care
Additional criteria if comorbid HIV	≥ 12 months on ART, undetectable viral load or CD4 cell count > 500 cells/mm^3^, no TB, ≥ two clinic visits for ART, not pregnant nor breastfeeding

*Abbreviations*: *ART* antiretroviral therapy, *HbA1c* hemoglobin A1C, *HIV* human immunodeficiency virus, *TB* tuberculosis

In the facility-based fast-track model, clients are given 3-month prescriptions and an appointment for their medication collection (Eswatini Ministry of Health: Standard operating procedures for NCD Fast-Track Model, unpublished). Both reduce the travel and waiting time a client has to spend to obtain diabetes and hypertension care. When a client arrives at the clinic, they do not have to queue with the other clients but rather is received by the health care personnel according to a pre-scheduled appointment. If no health concerns arise, the client collects their medication from a designated fast-track dispensing point at the facility. Because six-monthly visits and thorough health check-ups are required for clients with diabetes or hypertension, each fast-track visit is followed by a normal clinic visit 3 months later during which standard of care treatment guidelines are followed. After these visits, the client is given a 3-month drug prescription and a date for the next fast-track appointment. This model mainly targets the working population living and/or working near a facility.

The facility-based treatment club (FTC) DSD model involves groups of at most 20 clients with diabetes and/or hypertension (Eswatini Ministry of Health: Standard operating procedures for FTCs, unpublished). During bi-monthly treatment club meetings, the facilitator (usually the nurse) provides general health counseling on diabetes, hypertension, nutrition, exercise, HIV, and cervical and prostate cancer screening. Furthermore, the blood pressure, blood glucose, and weight of all participants are measured and clients receive medication for the next 2 months. Every 6 months, the clients receive a full health check-up at the clinic after the club meeting. If a client is unwell during any of the meetings, they are referred either to the clinic or a tertiary care facility for an immediate check-up. This model mainly targets clients living near a facility.

The third model, community advisory groups (CAGs), are groups of a maximum of six clients with diabetes and/or hypertension who meet monthly in their respective community (Eswatini Ministry of Health: Standard operating procedures for Community-based Adherence Groups, unpublished). Groups are composed of members of an existing social network, such as family members, friends, or colleagues. Groups are equipped with a digital blood pressure monitor and a point-of-care glucometer to monitor their blood pressure/glucose during each meeting. Members are trained on the use of these devices, screening each other for tuberculosis, and referral procedures. Team members take turns in collecting the medication for the entire group from the clinic. This ensures that clients receive the recommended health check-up at least every 6 months and obtain their medication without having to visit the clinic each month. Each group is assigned a nurse that can be contacted in case of challenges and questions. This model primarily targets clients living in remote areas.

(3) Treatment arm 2: Community distribution points

The second treatment arm, the community distribution point (CDP) arm, leverages existing community outreach sessions that offer HIV care and now additionally offer services for clients with diabetes and/or hypertension. CDPs are sites that are set up temporarily within a community once a month in central locations, such as community centers or schools. Each CDP is linked to one clinic, which means that CDPs are organized through this clinic. A team consisting of a nurse and additional health personnel offer diabetes and hypertension screening, treatment initiation, adherence support, and counseling according to the standardized treatment guidelines described above.

#### Rural Health Motivators—the government-led community health worker cadre

Evidence from high-income countries shows that the involvement of community-based health workers to provide support to people living with chronic health conditions can improve linkage to, and retention in, care through health counseling and treatment adherence support at the community or household level [14]. It may also allow other cadres, such as nurses and physicians, to focus on care for people with more complex problems as tasks that do not require extensive medical training, such as providing health information or following up with clients who missed appointments, can be carried out by community health workers (CHWs) [13]. CHWs, therefore, have the potential to play a critical role in scaling up client-centered care for diabetes and hypertension in Eswatini and other LMICs. However, the extent to which CHW programs for chronic health conditions in LMICs can achieve this goal, and which delivery models are likely to be most effective, feasible, and acceptable, need to be systematically evaluated. As of now, comprehensive and rigorous evidence on the effectiveness of CHW involvement in health care provision for diabetes and hypertension in LMICs is scarce.

Both treatment arms of the WHO-PEN@Scale study involve the government-led and government-funded community health worker cadre, the Rural Health Motivators (RHMs), in addition to the service delivery models described above. The RHM program is a community-based health care volunteer program, which was established in 1976 by the Eswatini Ministry of Health to facilitate the extension of health promotion services to the communities through interpersonal communication (Eswatini Ministry of Health: Community based health services: annual program report 2018, unpublished). There are over 3000 RHMs across the country. They are non-salaried and non-specialist and offer services focused on basic health promotion messaging for sexual and reproductive health, nutrition, and child health [15]. To ensure participation and ownership of the community, RHMs are chosen at the community level and trained by the Ministry of Health. 53% of RHMs is over the age of 55 and 94% is female. Additional details on the RHMs are available elsewhere [16, 15].

In WHO-PEN@Scale, RHMs are involved with the following tasks: (i) basic screening of clients at risk of diabetes and/or hypertension, (ii) extensive, personalized lifestyle counseling, (iii) referral of clients at risk of diabetes and/or hypertension, (iv) monitoring treatment adherence and health status of clients with diabetes and/or hypertension, and (v) demand creation for services offered in the respective treatment arm. RHMs screen clients based on their age, sex, and measured waist circumference. They are equipped with standardized guidelines on screening as well as health messaging in the form of a flip chart specifically developed for them. In the DSD arm, RHMs will refer clients to clinics, and in the CDP arm to the CDPs. Urgent cases will immediately be referred to primary care clinics or tertiary care facilities.

### Theory of change

**Fig. 2:**
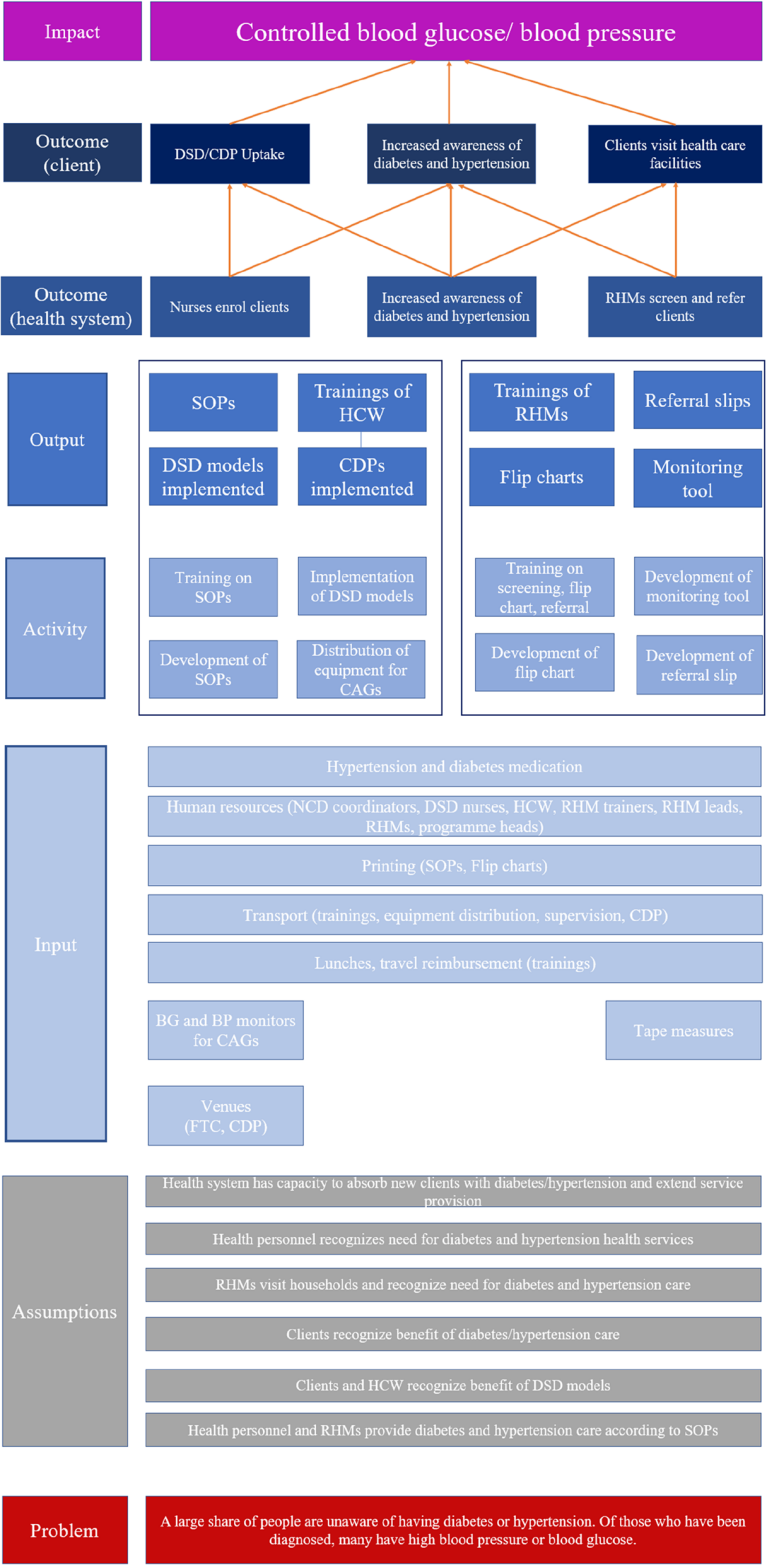
Theory of change. Abbreviations: BG = blood glucose, BP = blood pressure, CAG = community advisory group, CDP = community distribution point, DSD = differentiated service delivery, FTC = facility-based treatment club, HCW = health care worker, RHM = Rural Health Motivator, SOPs = standard operating procedures

Figure 2 details the theory of change for the WHO-PEN@Scale project. In this section, we describe this theory in more detail.

#### Problem

A large share of people living with diabetes or hypertension have not been diagnosed. Furthermore, blood glucose and blood pressure control are often not achieved even among those who are aware of their condition.

#### Assumptions

The WHO-PEN@Scale study relies on several assumptions. First of all, it is assumed that the health system in Eswatini has the capacity to absorb new clients who are linked to care because of the interventions. This includes the assumption that the health personnel has the capacity to extend the set of services offered to clients with diabetes and hypertension. Second, it is required that the needs for and benefits of diabetes and hypertension care are recognized across all levels. This includes the health personnel at the facilities, who are assumed to recognize that they can play an active role and contribute considerably to the improvement of diabetes and hypertension prevention and control by extending health services offered at the primary care clinic level. In addition, it needs to be assumed that the RHMs visit a given number of households and recognize that their clients are either at risk of diabetes or hypertension or in need of better care. Finally, clients also need to recognize that diabetes and hypertension have severe health consequences in the long run and that they can benefit from care even if, at a given moment, they feel healthy. Third, for the DSD arm, it is assumed that clients and health care workers see the benefit of the DSD models. Although it might lead to a higher work load at first, once established, these models will ease the burden on healthcare workers by saving time and resources. Clients might need to incorporate the DSD model into their life routine and adhere to scheduled meetings, for example. Despite these upfront costs, it is assumed that clients understand that in the long term they will benefit from the change in the service provision model. The last assumption is that health care workers and RHMs provide health services according to the standard operating procedures developed by the Ministry of Health.

#### Input

The WHO-PEN@Scale project requires several inputs. If an increasing number of people living with diabetes or hypertension are linked to care, there will be a larger demand for diabetes and hypertension medication. For the implementation of the interventions, additional human resources are required. For example, an NCD coordinator in each region or nurses preparing and implementing the DSD models and the CDPs. Furthermore, existing health personnel needs to be involved in the activities such as the health care workers and the different levels of the RHM program. For the implementation and training on the models themselves, resources for printing, transport, and lunches are required. The CAGs need to be equipped with point-of-care blood glucose and blood pressure measurement devices. The facility-based treatment club needs a meeting venue, ideally at the clinic. The implementation of the CDPs will require additional transport and a location where they can be set up. The RHMs need to be equipped with tape measures to measure waist circumference and flip charts for health education and lifestyle counseling.

#### Activities and output for the DSD models and CDPs

First, the SOPs need to be developed. Then, the main activities involve the training of the health care personnel on these SOPs, the enrolment of clients in the different DSD models, and the distribution of equipment to the CAGs. The expected outputs are the finalized standard operating procedures, the trainings of healthcare workers, and a number of DSD models as well as CDPs implemented.

#### Activities and output at the RHM level

For the RHMs, the flip chart, which is used to guide them during the household visit, and a monitoring tool need to be developed. Then, the RHMs need to be trained in the use of these instruments. The expected outputs are the RHM trainings and the finalized documents (flip charts, monitoring tool).

#### Outcomes

These inputs, activities, and outputs will result in several outcomes. At the health system level, awareness of diabetes and hypertension will have increased among health care workers and RHMs. Nurses will enroll clients in the DSD models or advise them to go to CDPs. RHMs will screen and refer clients at risk of diabetes and hypertension. At the client level, this will result in increased awareness and the uptake of DSD models and services provided at CDPs as well as clients seeking health care at primary care facilities following the RHM’s referral.

#### Impact

Finally, the implementation of WHO-Pen@Scale will result in better control of blood glucose and blood pressure among the population living with diabetes and/or hypertension.

### Trial duration

The funding for WHO-PEN@Scale started on January 1, 2019, which is also the study start. Recruitment of clients for the DSD and CDP arms started in November 2021. The WHO-PEN@Scale study will be completed on March 31, 2023.

### Randomization

The unit of randomization is a primary healthcare clinic along with all RHMs (and their corresponding service area) that are, as per the public health system structure, assigned to that clinic. There are 118 public primary healthcare facilities in Eswatini. The 97 clinics connected to the Client Management Information System (CMIS) at trial start (in January 2019) were eligible for inclusion in the study. The CMIS is the electronic health registry used by health personnel, which documents clients’ disease trajectory, health-seeking behavior, medication prescription, and health status. Of these 97 clinics, nine had to be excluded because a major non-governmental organization (NGO) was planning to implement different health service decentralization models for NCDs there and four (one per region) were excluded because they were determined to be pilot clinics by the Ministry of Health. The remaining 84 were evenly randomized across study arms.

We conducted a stratified randomization. Clinics were randomly allocated to the three study arms stratified by region (with there being four regions in Eswatini), rural/urban location of the clinic, and education (share of the population living in the clinic’s 5-km catchment area with completed secondary education or higher). The randomization was conducted by the Heidelberg Institute of Medical Biometry, which is not involved in the study and thus an independent entity. During the preparation for implementation, it became clear that seven clinics were unable to implement the interventions and needed to be replaced with clinics without CMIS. Randomization of the replacements was stratified by region only as there were no matching clinics based on rural–urban location or educational profile of the surrounding population. The random sampling of replacement clinics was undertaken by study staff at the Heidelberg Institute of Global Health. The code and seed used were the same as in the first round of randomization.

### Endpoints

The primary outcomes are (i) mean glycated hemoglobin (HbA1c) among adults aged 40 years and older with diabetes and (ii) mean systolic blood pressure among adults aged 40 years and older with hypertension. For definitions of diabetes and hypertension, see Table 6.

**Table 6:** Definition of population included in outcome analysis

Prediabetes	HbA1c ≥ 5.7% and ≤ 6.4%
Diabetes	HbA1c > 6.4% or Self-reported previous diabetes diagnosis
Hypertension	Systolic blood pressure ≥ 140 mmHg or Diastolic blood pressure ≥ 90 mmHg or Self-reported previous hypertension diagnosis

The secondary outcomes are:

1. Mean HbA1c among adults aged 40 years and older with diabetes or prediabetes
2. Proportion of adults aged 40 years and older with diabetes who have an HbA1c less than 7.0% (glycemic control)
3. Proportion of adults aged 40 years and older with diabetes who report to have ever been tested for diabetes
4. Proportion of adults aged 40 years and older with diabetes or prediabetes who report to have ever been tested for diabetes
5. Proportion of adults aged 40 years and older with diabetes who report to have been diagnosed with diabetes prior to the household survey
6. Proportion of adults aged 40 years and older with diabetes or prediabetes who report to have been diagnosed with diabetes or prediabetes prior to the household survey
7. Proportion of adults aged 40 years and older with diabetes who report to be taking medication for their diabetes
8. Mean systolic blood pressure among adults aged 40 years and older with diabetes
9. Mean diastolic blood pressure among adults aged 40 years and older with diabetes
10. Mean systolic blood pressure among adults aged 40 years and older with diabetes or prediabetes
11. Mean diastolic blood pressure among adults aged 40 years and older with diabetes or prediabetes
12. Mean diastolic blood pressure among adults aged 40 years and older with hypertension
13. Proportion of adults aged 40 years and older with hypertension who have a systolic blood pressure < 140 mmHg and a diastolic blood pressure < 90 mmHg (hypertension control)
14. Proportion of adults aged 40 years and older with hypertension who report to have ever had their blood pressure measured
15. Proportion of adults aged 40 years and older with hypertension who report to have been diagnosed with hypertension prior to the household survey
16. Proportion of adults aged 40 years and older with hypertension who report to be taking blood-pressure-lowering medication
17. Proportion of adults aged 40 years and older with diabetes, prediabetes, or hypertension who report to be a current smoker
18. Proportion of adults aged 40 years and older with diabetes, prediabetes, or hypertension who report to be drinking alcohol daily.
19. Mean number of minutes in a typical week spent doing moderate- or vigorous-intensity exercise among adults aged 40 years and older with diabetes, prediabetes, or hypertension
20. Proportion of adults aged 40 years and older with diabetes, prediabetes, or hypertension who correctly responded to each individual question on diabetes- and hypertension-related knowledge.

The primary and secondary outcomes will be assessed via a household survey conducted in the areas served by the RHMs that are assigned to each study clinic.

#### Sampling strategy for the household survey

In Eswatini’s public sector health system, each RHM is assigned an area within their community. We refer to these areas as RHM service areas. Within each service area, all households are served by only one RHM. RHM service areas do not overlap (that is, it is never the case that two RHMs serve all or part of the same area). For clinics with more than ten assigned RHMs, we will randomly select ten RHMs with their corresponding service area. The households in each RHM service area will be sampled based on a “random walk” procedure. The starting point for the random walk is the RHM’s homestead, which can be located anywhere in their service area. When having arrived at the RHM’s homestead, the survey team will obtain information from the RHM on the number of households in, and the geographical boundaries of, the service area. Then, the team will randomly generate a number (using a random number generator on a tablet) from one to four to determine in which direction they will move (North, East, South, or West). All households in this direction will be included in the survey. If the survey team reaches the RHM service area boundary before completing the required number of interviews, they return to the RHM homestead and generate a new random number that defines the next direction in which the survey team will walk. Whether or not the RHM provided the survey team with accurate information on the service area boundaries will be verified by the survey team by also visiting the first household beyond the border of the service area to enquire from the household head which RHM serves their household. For clinics with less than ten RHMs, all RHM service areas, and all households within these areas, will be visited by the survey team for inclusion in the survey.

#### Inclusion and exclusion criteria for the household survey

All non-pregnant household members aged 40 years or older will be invited to participate in the survey. The first part of the survey, which has as its main purpose the screening of all adults aged 40 years or older for prediabetes, diabetes, and hypertension, consists of (i) a short interviewer-administered questionnaire (see Additional file 1) covering previous diagnoses and current treatment of diabetes and hypertension, (ii) blood pressure measurements, and (iii) fasting blood glucose and HbA1c measurements. For the impact assessment, those identified as having prediabetes, diabetes, or hypertension based on this screening questionnaire and following the definitions in Table 6 will additionally be included in the survey for a more detailed assessment of self-reported characteristics as well as body measurements (see Additional file 1). For the analysis of syndemic processes, a random subsample of 600 participants aged 40 years and older who do not have prediabetes, diabetes, or hypertension based on the screening questionnaire results and definitions in Table 6 will also be included in these more extensive assessments. Apart from pregnancy, the only exclusion criterion is an inability to provide written informed consent.

#### Return visits

Upon arrival at the household, the interviewer will compile a list of all household members aged 30 years and older. If one or more eligible household members are not at home and do not return while the survey team is still at the household, the interviewer schedules a second visit either on the same or on another day. If the household member is still not available on the second visit, a third visit is scheduled. If the household member is not available again, the household member is marked as “not at home”. If a homestead is occupied but no member is present, the survey team will visit on two additional days at different times. If no household member is met during these visits, the household is marked as “not at home”.

#### Survey instrument modules

As described above, the objective of the household screening questionnaire is to identify all non-pregnant household members aged 40 years and older who have prediabetes, diabetes, and/or hypertension. The survey team will visit the household, schedule a visit for the interview, and ask household members aged 40 years and older to not eat or drink anything in the 12 h before the interview. On the day of the interview, blood pressure will be measured. If the systolic blood pressure is 140 mmHg or higher or the diastolic blood pressure is 90 mmHg or higher, a second measurement will be taken for confirmation and the average calculated. Then, fasting blood glucose will be measured. For individuals with a fasting blood glucose level of 5.6 mmol/L or higher, HbA1c will be measured for confirmation of prediabetes/diabetes. Fasting blood glucose and HbA1c measurements will be taken with point-of-care devices. The extensive interviewer-administered questionnaire (administered to all those identified as having prediabetes, diabetes, or hypertension in the screening module and the random subsample of 600 participants for the syndemics analysis) will cover (a) socio-demographic and economic characteristics such as marital status, education, and religion, (b) history of diabetes and hypertension and health-seeking behavior for each of the two conditions, (c) visits and services provided by RHMs, (d) health care utilization and related expenditure, (e) treatment adherence and knowledge on diabetes and hypertension, (f) behavioral cardiovascular disease risk factors such as tobacco and alcohol use, and physical activity, and (g) anxiety and depression. There will be the following additional body measurements: (a) height (using a tape measure against a wall) and weight (using a scale, which is zeroed prior to each day of study procedures) to calculate body mass index (BMI), (b) a waist circumference (using tape measures), and (c) a rapid HIV test.

The household-level survey module will be administered to the head of the household, or, if not present, another adult household member with sufficient knowledge of their household characteristics, and cover (i) access to water and sanitation, (ii) household expenditure, (iii) ownership of assets and livestock, (iv) housing characteristics, and (v) non-health-related household expenditures.

### Power calculations

The number of participants to be included in the household survey was determined through a power calculation. This power calculation accounts for clustering at the level of the unit of randomization (i.e., at the level of a healthcare facility with its assigned RHM service areas). Our power calculation assumes that the intra-cluster correlation coefficient does not exceed 0.04 for the blood pressure outcome and 0.08 for the diabetes outcome. These estimates are based on the intra-cluster correlation coefficient for primary sampling units from the 2012 South African National Health and Nutrition Examination Survey (SANHANES) [17], which is a nationally representative household survey that measured both blood pressure and HbA1c among adults aged 40 years and older. We expect this estimate to be conservative as we sample several areas for each cluster. Based on our sampling strategy for the household survey, we also assume a coefficient of variation of cluster sizes of 0.4. The minimum effect size that we want to be able to detect is an absolute difference in mean HbA1c of 0.8% (primary diabetes endpoint) and an absolute difference in mean systolic blood pressure of 5.0 mmHg (primary hypertension endpoint). Lastly, we assume a mean HbA1c among adults (aged ≥ 40 years) with diabetes of 8.2% with a standard deviation of 2.3%, and a mean systolic blood pressure among adults (aged ≥ 40 years) with hypertension of 153 mmHg with a standard deviation of 22.5 mmHg. These numbers were again taken from the 2012 SANHANES [17]. We aim for a minimum of 80% statistical power. Under these assumptions, we require 224 participants per study arm for the diabetes endpoint, and 560 participants per study arm for the hypertension endpoint. These power calculations have been performed using the clustersampsi package in Stata version 15 [18].

### Statistical analysis

#### Impact of WHO-PEN@Scale on population health

To determine whether the WHO-PEN@Scale health service intervention is more effective than the standard of care, we will pool data from the two treatment arms and compare them to the standard of care. In a second step, we will compare data between the two treatment arms to determine which health service decentralization strategy is more effective as well as compare each treatment arm individually to the standard of care. We will estimate ordinary least squares regression models to compare mean HbA1c among adults with diabetes and mean systolic blood pressure among adults with hypertension between the study arms. All regression models will regress the outcome onto a binary indicator for the intervention as well as each stratum, and adjust standard errors for clustering at the level of the unit of randomization (the primary healthcare facility and its assigned RHM areas). In secondary analyses, we will also include participants’ socio-demographic characteristics as covariates, which is not expected to substantially affect the point estimates but may well reduce the variance. We will use a significance level of *p* < 0.05 for all analyses. Because there is disagreement in the research literature as to whether a multi-arm trial like WHO-PEN@Scale would require an adjustment for multiple hypothesis testing [19], we will use a *p*-value < 0.05 to indicate significance in these comparisons (i.e., we will not adjust for multiple hypothesis testing). We will also conduct subgroup analyses for males vs. females, 10-year age groups, education categories, household wealth quintiles, and rural vs. urban household residency. We are not planning to impute any missing data.

#### Synergistic interactions between various diseases

To assess any synergistic interactions between diabetes, hypertension, HIV, and other conditions [20] and to provide insight for the tailoring of diabetes and hypertension care for people with such comorbidities, we will conduct supplementary mixed-methods research, drawing from epidemiology, medical anthropology, and political science. First, based on data from the household survey, we will identify potential synergistic interactions. Subsequently, using qualitative research methods and qualitative comparative analysis, we will examine structural factors along the life course, such as gender, poverty, forced migration, stigma, and unequal social/economic relationships, to assess how they influence the resultant morbidity related to diabetes, hypertension, HIV, and other chronic conditions, and their co-occurrence.

(i) Quantitative assessment of any syndemics

The household survey will provide in-depth information on participants with prediabetes, diabetes, and/or hypertension. In addition, we will survey a random sample of household members with neither prediabetes, diabetes, nor hypertension, administering to them the same questionnaire as to the study sample. This strategy will yield representative data on the co-occurrence of diabetes, hypertension, HIV, and other pathologies, including depression, at the population level. The survey will also measure relevant structural factors at the individual and household levels, including socio-economic status. We will then use structural equation modelling (SEM) to obtain variance and co-variance estimates between the variables and latent constructs that they might describe [21]. Assessing a multitude of SEM models will provide insight into the optimal representation of the interplay between the variables, and as such identify the most appropriate framework that concurs with the obtained data.

(ii) In-depth life-history interviews

After characterizing the syndemic in terms of identifying any exacerbation of morbidity, i.e., emergent morbidity from co-occurrence of diabetes and HIV, we will conduct life histories with a subsample of respondents in this population group to explore the relevant structural factors [22]. A diversity-sampling approach will be taken to ensure that male and female respondents without diabetes and hypertension, living in rural and urban communities, over varied ages will be recruited. The total sample will also be guided by the point of theoretical saturation, whereby no more novel information is elicited [23]. Life-history interviews will examine contextual factors that are likely to interact with and compound the morbidity burden linked to NCDs, HIV, and other conditions. We will also examine factors that potentially contribute to the co-occurrence of these diseases. These will include violence, poverty, stigma, and unequal social/economic relationships. The interviews will encompass specific events and periods during the life course. With the consent of respondents, interviews will be audio-recorded, transcribed, and translated for qualitative content analysis.

(iii) Qualitative comparative analysis

Typically, qualitative comparative analysis (QCA) is used by scholars engaged in the qualitative and intensive study of macro social phenomena, particularly in sociology and political sciences [24]. It is, furthermore, a useful approach for the analysis of multiple in-depth case studies at any scale. This approach provides tools to untangle complex combinations of explanatory factors related to particular outcomes, when studying a relatively small number of cases (from around 5 to 50) and when traditional statistical methods are not appropriate. The approach also enables qualitative and quantitative data to be analyzed together using a systematic approach [25]. Applying QCA, analysts are able to identify more than one pathway that are potentially causal to an outcome, whether factors are potentially causal only if in conjunction with other conditions, and whether pathways that lead to an outcome differ from those that fail to achieve the outcome.

We will use the participants recruited for the in-depth life-history interview as cases for QCA. Data for analysis will be drawn from the thematic qualitative analysis of these interviews and from the household survey. Under the syndemic framework, we hypothesize that a series of (social and health-related) conditions contribute to the co-occurrence of HIV and NCDs and/or exacerbated morbidity caused by co-occurrence and interaction of these conditions. We will therefore be able to combine qualitative and quantitative data to investigate conditions that are potentially causal for an outcome, for example, the presence of emergent (or excess) morbidity from the co-occurrence and interaction of HIV and diabetes, or conditions for co-occurrence. Such conditions might include violence, poverty, stigma, and unequal social/economic relationships. We will pay particular attention to the gendered impact of these factors. Using these conditions, a “truth table” will be constructed. This will help to identify the conditions that are necessary and/or sufficient for specific outcomes. The analysis will also include consideration for the temporal distribution of conditions across the life course [26].

## Oversight and monitoring

The Heidelberg Institute of Global Health study team is the coordinating center of the WHO-PEN@Scale study. It coordinates and oversees all research activities and ensures that they are in line with the study protocol. It presides the Project Management Committee (PMC), which is comprised of one representative of each of the nine consortium partners, and acts as the supervisory body of the study as well as the ultimate decision-making body of the consortium. The PMC meets on a monthly basis and ad hoc meetings are scheduled if required. The Scientific Steering Committee is the advisory board and includes five members with relevant expertise including in research methods and/or cardiovascular disease care in low- and middle-income settings. Four of these members also comprise the Ethics Advisory Board, which is updated semi-annually.

The Eswatini Ministry of Health coordinates the implementation of the study arms, which includes the recruitment of clients for the DSD models and CDPs. They ensure that the study is conducted according to the Eswatini Health and Human Research Review Board (EHHRRB). As explained below, the EHHRRB receives a progress report of the study and reviews whether the study complies with all ethical guidelines. During the design phases of the study arms, the Project Implementation Technical Team (PITT) was leveraged. The PITT comprises representatives of all government departments that are involved in the NCD health service decentralization, NGOs working in health service provision for chronic diseases, and all Eswatini-based consortium partners and met on a weekly basis. The PITT contributed considerably in forging the study design to bring a maximum benefit to the Eswatini population. Furthermore, the on-the-ground experience from all PITT members and their diverse backgrounds ensured that the study design was appropriate in the Eswatini context and the heterogeneity of needs and behaviors across population groups. The Clinton Health Access Initiative (CHAI) coordinates all data collection activities on the ground and reports to the PMC in a meeting on a monthly basis as well as to the Heidelberg Institute of Global Health in weekly meetings.

## Trial status and implementation timeline

In 2019, the study arms were initially designed, standard operating procedures developed, and components of the treatment arms piloted and adapted. Training of the 1280 RHMs in both treatment arms started in April 2021, with the majority of trainings conducted between August and November 2021. Training of health care workers at the DSD clinics started in September 2021 and was concluded in January 2022. Once trained, nurses started organizing the clinic-based DSD models. The first models were launched in November 2021. While DSD models are extended on a rolling basis to meet the increased demand for diabetes and hypertension care, implementation was considered to have been completed once each DSD arm clinic offered at least one DSD model, which was achieved in April 2022. Healthcare workers in the CDP arm were trained between January and March 2022. Once the trainings were concluded, healthcare workers started to offer diabetes and hypertension services during CDP sessions. Thus, implementation for the CDP arm was considered to have been completed in March 2022. The household survey is planned to be conducted between October 2022 and January 2023.

### Dissemination

The results will be communicated directly to the Eswatini Ministry of Health in the form of policy briefs, presentations, and extensive reports. They will furthermore be reported to the European Commission in an extensive report. The dissemination on the ground will be led by the CHAI, Diabetes Eswatini, and Eswatini Business Health and Wellness, which will hold stakeholder meetings to increase the reach of the results. Furthermore, all results will be published in peer-reviewed journals. Authorship will be defined according to the rules of the International Committee of Medical Journal Editors and, for non-medical journals, the publication agreement set up and signed by all involved institutions.

## Discussion

The feasibility pilot conducted in the Lubombo region in 2016 showed that including primary healthcare facilities in the provision of healthcare for diabetes and hypertension improved population health [11]. The encouraging results led the Eswatini Ministry of Health to the conclusion that a health service decentralization to even lower levels, i.e., the community and household levels, can bring additional benefits. Although in the standard of care tasks have already been shifted from physicians in tertiary care facilities to nurses in primary care clinics, the two treatment arms include service provision models that have the ambition of being tailored to the needs of the population living with diabetes or hypertension. We expect that the involvement of RHMs in both treatment arms will create additional demand for diabetes and hypertension health services and reach individuals that do not regularly visit healthcare facilities.

This study will contribute further evidence on the effectiveness of task-shifting and the inclusion of community health workers in healthcare provision for diabetes and hypertension. It will be crucial in informing health care provision for diabetes and hypertension in Eswatini and form the basis for a health system reform on a national scale. Furthermore, the findings may also prove valuable to other governments in sub-Saharan Africa when reforming their health care systems in order to meet the growing need for diabetes, hypertension, and other chronical disease care.

The impact evaluation described in this protocol will be accompanied by several additional analyses, such as the acceptability, implementation, and cost-effectiveness studies. We believe that the complementarity and comprehensiveness of all these studies will yield highly relevant insights for the Eswatini government as well as the governments of other sub-Saharan African countries. We will not only evaluate if the implemented interventions are effective but also generate further insights as to why they do or do not work. This should allow for an in-depth understanding of the underlying causal processes as well as the acceptability of the interventions by clients and health workers.

## Supplementary Information

**Additional file 1.** 

## Data Availability

The deidentified full dataset from our household survey along with all code for data management and analysis will be posted in a publicly accessible data repository upon publication of the findings for the primary endpoints.

## Abbreviations

ART: Antiretroviral therapy
BMI: Body mass index
BG: Blood glucose
BP: Blood pressure
CAG: Community Advisory Group
CDP: Community distribution point
CHAI: Clinton Health Access Initiative
CHW: Community health worker
CMIS: Client Management Information System
DSD: Differentiated service delivery
EHHRRB: Eswatini Health and Human Research Review Board
FTC: Facility-based treatment club
HbA1c: Glycated hemoglobin
HCW: Health care worker
HIV: Human immunodeficiency virus
LMIC: Low- and middle-income country
NCD: Non-communicable disease
NGO: Non-governmental organization
PITT: Project Implementation Technical Team
PMC: Project Management Committee
QCA: Qualitative comparative analysis
RHM: Rural Health Motivator
SANHANES: South African National Health and Nutrition Examination Survey
SEM: Structural equation modelling
SoC: Standard of care
SOP: Standard operating procedures
TB: Tuberculosis
WHO: World Health Organization
