# Supplementary material for: Strengthening primary care for diabetes and hypertension in Eswatini: study protocol for a nationwide cluster-randomized controlled trial
Source: Trials. 2023 Mar 22;24:210. doi: 10.1186/s13063-023-07096-4 (PMC10031170; doi:10.1186/s13063-023-07096-4)
Supplement: Supplementary file 1 — Additional file 1. [file 13063_2023_7096_MOESM1_ESM.pdf]

Additional file 1

Strengthening primary care for diabetes and hypertension in Eswatini: study protocol for a nationwide cluster-randomized controlled trial

WHO-PEN@Scale HH level

Field	Question	Answer
Section 1: Survey information		
hh_si12 *(required)*	si12: Enumerator ID<br>*Please enter your initials and the tablet number (e.g. JD50)*	
hh_gps_auto *(required)*	Automatically recorded location<br>*GPS coordinates can only be collected when outside.*	
hh_hhid *(required)*	Unique household identifier<br>*Please enter HHID in the following format: DD-MM-Data Collector ID-Household Number, e.g., 07-10-JD50-01"*	
hh_hhidcheck *(required)*	Please re-enter the unique household identifier<br>*Please enter HHID in the following format: DD-MM-Data Collector ID-Household Number, e.g., 07-10-JD50-01"*	
generated_note_name_11 *(required)*	The household IDs do not match. Please check and enter correctly<br>*Question relevant when: ${hh_hhid} != ${hh_hhidcheck}*	
hh_si13 *(required)*	si13: Will you collect the household-level information?	1 Yes<br>2 No
hh_si13xx *(required)*	si13xx: Why are you not going to collect the household level information?<br>*Question relevant when: ${hh_si13} = '2'*	
Household level information<br>*Group relevant when: ${hh_si13} = '1'*		
generated_note_name_19	Instruction: The final module will collect information at the household level. Look for the most knowledgable household member who will be in the best position to answer questions on household characteristics and expenses.	
Household level information > Section HH1: Household characteristics		
hc1 *(required)*	Read: "*Now I would like to ask you some questions about your household*".<br><br>hc1: What is the main source of drinking water?	1 Piped into dwelling<br>2 Piped into yard/plot<br>3 Piped to neighbour<br>4 Public tap/standpipe<br>5 Tubewell/Borehole<br>6 Protected well<br>7 Unprotected well<br>8 Rainwater<br>9 Tanker truck<br>10 Cart with small tank<br>11 Surface water (River/dam/lake/pond…)<br>12 Bottled water<br>98 Other, specify
hc1x *(required)*	hc1x: Specify other main source of drinking water<br>*Question relevant when: ${hc1} ='98'*	
hc2 *(required)*	hc2: What kind of toilet facility do members of your household usually use	1 Flush to piped sewer system<br>2 Flush to septic tank<br>3 Flush to pit (latrine)<br>4 Flush to somewhere else<br>5 Flush to unknown place / Not sure / DK where<br>6 Ventilated Improved Pit latrine (VIP)<br>7 Pit latrine with slab<br>8 Pit latrine without slab / Open pit<br>9 Bucket<br>10 No facility, Bush, Field<br>98 Other, specify
hc2x *(required)*	hc2x: Specify other toilet facility<br>*Question relevant when: ${hc2} ='98'*	
hc3 *(required)*	hc3: Do you share this toilet facility with other households?<br>*Question relevant when: ${hc2} !='10'*	1 Yes<br>2 No<br>77 Don't know<br>88 Refused
hc4 *(required)*	hc4: What type of cooking fuel does your household mainly use for cooking?	1 Electricity<br>2 Liquefied Petroleum Gas (LPG)<br>3 Natural gas<br>4 Biogas<br>5 Kerosene<br>6 Coal / Lignite<br>7 Charcoal<br>8 Wood<br>9 Straw/Shrubs/Grass<br>10 Animal dung (Bulongwe)

Field	Question	Answer
		11 Agricultural crop residue<br>12 No food cooked in household<br>98 Other, specify
hc4x *(required)*	hc4x: Specify other type of cooking fuel<br>*Question relevant when: ${hc4} ='98'*	
Household level information > Section HH1: Household characteristics > Livestock		
generated_note_name_29	hc7: How many of the following livestock does this household own?<br>*Livestock shared with other households but not owned by your household is not to be included*	
hc7a *(required)*	Cattle / milk cows / bulls	
hc7b *(required)*	Horse / donkey / mules	
hc7c *(required)*	Goats	
hc7d *(required)*	Sheep	
hc7e *(required)*	Chickens / poultry	
hc7f *(required)*	Pigs	
hc7g *(required)*	Other livestock types	1 Yes<br>2 No<br>77 Don't know<br>88 Refused
hc7h *(required)*	hc7h: How many other livestock types?<br>*Question relevant when: ${hc7g} ='1'*	
Household level information > Section HH1: Household characteristics > Other animal types (1)<br>*Group relevant when: ${hc7h} >'0'*		(Repeated group)
animalsxname *(required)*	Which other livestock does this household own?	
animalsxquant *(required)*	How many [animalsxname] does this household own?	
hc8 *(required)*	hc8: Does any member of this household own any agricultural land?	1 Yes<br>2 No<br>77 Don't know<br>88 Refused
hc9 *(required)*	hc9: How many (football) fields of agricultural land do members of this household own?<br>*1 football field = 0.7 hectares*<br>*Question relevant when: ${hc8} ='1'*	
Household level information > Section HH1: Household characteristics > Assets		
generated_note_name_48	hc10: Does your household, or any member of the household, have:<br>*Read out each item.*	
hc10	Options	1 Yes<br>2 No<br>77 Don't know<br>88 Refused
hc10a *(required)*	Electricity	1 Yes<br>2 No<br>77 Don't know<br>88 Refused
hc10b *(required)*	Radio	1 Yes<br>2 No<br>77 Don't know<br>88 Refused
hc10c *(required)*	Television	1 Yes<br>2 No<br>77 Don't know<br>88 Refused
hc10d *(required)*	Telephone (Landline or mobile)	1 Yes<br>2 No<br>77 Don't know<br>88 Refused
hc10e *(required)*	Computer	1 Yes<br>2 No<br>77 Don't know<br>88 Refused
hc10f *(required)*	Refrigerator	1 Yes<br>2 No<br>77 Don't know<br>88 Refused
hc10g *(required)*	Watch	1 Yes<br>2 No<br>77 Don't know<br>88 Refused
hc10h *(required)*	Bicycle	1 Yes<br>2 No

Field	Question	Answer
		77 Don't know<br>88 Refused
hc10i *(required)*	Motorcycle/Scooter	1 Yes<br>2 No<br>77 Don't know<br>88 Refused
hc10j *(required)*	Car or Van	1 Yes<br>2 No<br>77 Don't know<br>88 Refused
hc10k *(required)*	Tractor	1 Yes<br>2 No<br>77 Don't know<br>88 Refused
hc10l *(required)*	Animal Drawn Cart	1 Yes<br>2 No<br>77 Don't know<br>88 Refused
hc10m *(required)*	Sleeping Mat	1 Yes<br>2 No<br>77 Don't know<br>88 Refused
hc10n *(required)*	Bed	1 Yes<br>2 No<br>77 Don't know<br>88 Refused
hc10o *(required)*	Pot	1 Yes<br>2 No<br>77 Don't know<br>88 Refused
hc10p *(required)*	Hoe	1 Yes<br>2 No<br>77 Don't know<br>88 Refused
hc11 *(required)*	hc11: Observe the main material of the floor of the dwelling. Record observation. If not observable, ask the respondent.	1 Earth/ Sand<br>2 Dung<br>3 Wood planks<br>4 Palm/Bamboo<br>5 Parquet or polished wood<br>7 Ceramic tiles<br>8 Cement<br>9 Carpet<br>77 Don't know<br>88 Refused
hc12 *(required)*	hc12: Observe the main material of the roof of the dwelling. Record observation. If not observable, ask the respondent.	1 No Roof<br>2 Thatch/Palm leaf/ Grass<br>3 Sod<br>4 Rustic mat<br>5 Palm/Bamboo<br>6 Wood planks<br>7 Cardboard<br>8 Corrugated iron<br>9 Wood<br>10 Calamine/Cement fibre<br>11 Ceramic tiles<br>12 Cement/ Concrete<br>13 Asbestos<br>77 Don't know<br>88 Refused
hc13 *(required)*	hc13: Observe the main material of the exterior walls of the dwelling. Record observation. If not observable, ask the respondent.	1 No walls<br>2 Cane/Palm/Trunks<br>3 Stick & mud<br>4 Grass<br>5 Bamboo with mud<br>6 Stone with mud<br>8 Plywood (Off cuts)<br>9 Cardboard/ Carton

Field	Question	Answer
		10 Reused wood<br>11 Cement, cement blocks<br>12 Stone with lime/cement<br>13 Bricks<br>15 Mud blocks<br>16 Wood planks/shingles (new)<br>77 Don't know<br>88 Refused
Household level information > Section HH2: Socio-economic information		
se1 *(required)*	se1: How much is your monthly household expenditure on perishable and non-perishable food? (in Lilangeni/Rand)<br>*Ask for the person who usually does the grocery shopping for the household. Enter 777777 if the respondent did not know and 888888 if she refused to answer.*	
Household level information > Section HH2: Socio-economic information > Food		
se2 *(required)*	se2: How many people in your household eat from the food every week?<br>*Enter 777777 if the respondent did not know and 888888 if she refused to answer.*	
se4 *(required)*	se4: How much does your household spend on housing including utilities such as electricity and water every month? (in Lilangeni/Rand)	
se5 *(required)*	se5: How much does your household spend on non-food and non-housing purchases every month? (include education expenditure here) (in Lilangeni/Rand)<br>*For example, travel and transportation, entertainment, cell phone, bills, and education. Enter 777777 if the respondent did not know and 888888 if she refused to answer.*	
se6	se6: How many adults (age 18 years and older) household members does this household have?	

WHO-PEN@Scale Questionnaire

Field	Question	Answer
Section 1: Survey information		
i_si12 *(required)*	si12: Enumerator ID<br>*Please enter your initials and the tablet number (e.g. JD50)*	
i_gps_auto *(required)*	Automatically recorded location<br>*GPS coordinates can only be collected when outside.*	
i_hhid *(required)*	Unique household identifier<br>*Please enter HHID in the following format: DD-MM-Data Collector ID-Household Number, e.g., 07-10-JD50-01"*	
i_hhidcheck *(required)*	Please re-enter the unique household identifier<br>*Please enter HHID in the following format: DD-MM-Data Collector ID-Household Number, e.g., 07-10-JD50-01"*	
generated_note_name_9 *(required)*	The household IDs do not match. Please check and enter correctly<br>*Question relevant when: ${i_hhid} != ${i_hhidcheck}*	
i_ss2 *(required)*	What is the household member's name/initials?<br>*Make sure that the name/initials are the same as in the household roster!*	
i_si13 *(required)*	si13: Will you screen and interview [i_ss2]?	1 Yes<br>2 No
i_si13xx *(required)*	si13xx: Why are you not going to screen and interview [i_ss2]?<br>*Question relevant when: ${i_si13} = '2'*	
Individual level information<br>*Group relevant when: ${i_si13} ='1'*		
ps0 *(required)*	ps0: Can you please tell/show us your national ID number?<br>*Insert 88888 if no ID number is available.*	
Individual level information > Section 4: Consent for individual interview		
screeningconsentinfo	**Information Sheet for Introducing the Participant to the Study**<br><br>*Study Title:*<br>"WHO-PEN at Scale": Strengthening primary healthcare delivery for diabetes and hypertension<br><br>*Study Team: *<br>Amsterdam Institute for Global Health and Development, The Netherlands<br>Clinton Health Access Initiative, Eswatini<br>Diabetes Eswatini<br>Eswatini Business and Health & Wellness, Eswatini<br>Heidelberg University Hospital, Germany<br>Swiss Tropical and Public Health Institute, Switzerland<br>University of Eswatini, Eswatini<br>University of Goettingen, Germany<br><br>Dear Sir or Madam,<br><br>thank you very much for your consideration to participate in this study. You have been randomly selected to be part of this survey and this is why we would like to interview you.<br><br>*Background:*<br>Diabetes and hypertension contribute considerably to premature deaths as well as disability in Eswatini. The study team supports the Ministry of Health to improve their health services for diabetes and high blood pressure in order to encounter this growing health threat.<br><br>*Objectives:*<br>The aim of this study is to determine the extent of the unmet need for care for diabetes and high blood pressure in Eswatini.<br><br>*Procedure:*<br>We estimate that this interview will last about 40 minutes.<br>You will have a drop of blood taken from the tip of your finger to be tested for sugar. This may cause some mild pain. Furthermore, we would like to take a blood pressure measurement for which a cuff is placed around your upper arm. You may feel some uncomfortable tightening around your arm for a few seconds. You will be informed about the results of all tests. In case you have a high blood sugar or high blood pressure, you will additionally be offered the following assessments:<br><br>• a more detailed interviewer-administered questionnaire<br>• another blood test that uses again a drop of blood from your finger<br>• weight and height measurements<br>• waist and hip circumference measurement	

Field	Question	Answer
	• rapid HIV test<br><br>*Data privacy:*<br>The information you provide is totally confidential and will not be disclosed to anyone. It will only be used for research purposes. Your name and other personal information will be removed, and only a code will be used to connect your answers without identifying you. You might be contacted by the survey team again in the future in case the team requires any clarification of your answers. Data will be published online but it will not contain any personal information such as your names or address. no third parties will ever have access to your personal information. We plan to store the data for 10 years.<br><br>*Risks and benefits:*<br>There are only minor risks when taking a blood drop, such as mild pain or feeling uncomfortable. However, you might benefit from learning about your health status regarding the diseases mentioned above and their risk factors such as Body Mass Index.<br><br>*Participation:*<br>Participation in this study is voluntary. You have the right to refuse that we interview you or that we ask you certain questions. You will incur no consequences should you decide not to participate in this study or to withdraw at any time. You can withdraw your participation by expressing this desire to me. Furthermore, even after the interview has been concluded, you can request that your data and all other material be destroyed. For this, please get in touch with Ntombi Ginindza (either via Email: ntombiginindza@yahoo.com or by calling 76134679) or Similo Simelane (Email: ssimelane@clintonhealthaccess or by calling 76374770)<br><br>The Eswatini Ethics Committee requires me to obtain your consent to participate in this discussion in writing. I have summarized what I just talked about on this information sheet. Please indicate your consent to participate by signing the consent form once you do not have any further questions and feel comfortable, and signal to me that you are ready to start.	
screeningconsent *(required)*	For participant:<br>Do you give consent to continue with the survey?	1 Yes<br>2 No
screeningconsentsign *(required)*	Participant signature<br>*Question relevant when: ${screeningconsent} = '1'*	
Individual level information > Section 5: Socio-economic information<br>*Group relevant when: ${screeningconsent} = '1'*		
ss7 *(required)*	ss7: Record [i_ss2]'s sex<br>*Please observe and record accordingly.*	1 Male<br>2 Female
generated_note_name_30	READ:<br><br>First, I would like to confirm your age and whether you are currently pregnant.<br>*Question relevant when: ${ss7} ='2'*	
generated_note_name_31	READ:<br><br>First, I would like to confirm your age.<br>*Question relevant when: ${ss7} ='1'*	
ss8	ss8: What is your date of birth?	
ss9 *(required)*	ss9: How old are you?<br>*If the respondent does not know, enter 777.*<br>*Question relevant when: ${ss8} =''*	
ss10 *(required)*	ss10: Are you pregnant?<br>*Question relevant when: ${ss7} ='2'*	1 Yes<br>2 No<br>3 Don't know
note_pregnant	The respondent reported to be pregnant and is thus not eligible for the screening. The interview with this respondent will be ended now. Please proceed with the screening of the next eligible household member (if there is one).<br>*Question relevant when: ${ss10} = '1'*	
Individual level information > Section 6.1: History of raised blood pressure<br>*Group relevant when: ${elig_age2} =1 and ${ss10} != '1'*		
hbp1 *(required)*	READ:<br><br>Now, I will ask you some questions on raised blood pressure.<br><br>hbp1: Have you ever had your blood pressure measured by a doctor or other health worker?<br>*Ask the participant to only consider measurements done by a doctor or health worker.*	1 Yes<br>2 No<br>77 Don't know<br>88 Refused
hbp2 *(required)*	hbp2: Have you ever been told by a doctor or other health worker that you have raised blood pressure or hypertension?<br>*Question relevant when: ${hbp1} = '1'*	1 Yes<br>2 No<br>77 Don't know<br>88 Refused

Field	Question	Answer
hbp3 *(required)*	hbp3: In the past two weeks, have you taken any drugs (medication) for raised blood pressure prescribed by a doctor or other health worker?<br>*Ask the participant to only consider drugs for raised blood pressure prescribed by a doctor or other health worker.*<br>*Question relevant when: ${hbp2} = '1'*	1 Yes<br>2 No<br>77 Don't know<br>88 Refused
Individual level information > Section 6.2: History of raised blood sugar<br>*Group relevant when: ${elig_age2} =1 and ${ss10} != '1'*		
hd1 *(required)*	READ:<br><br>Now, I will ask you some questions on raised blood sugar.<br><br>hd1: Have you ever had your blood sugar measured by a doctor or other health worker?<br>*Ask the participant to only consider measurements done by a doctor or health worker.*	1 Yes<br>2 No<br>77 Don't know<br>88 Refused
hd2 *(required)*	hd2: Have you ever been told by a doctor or other health worker that you have raised blood sugar or diabetes?<br>*Question relevant when: ${hd1} = '1'*	1 Yes<br>2 No<br>77 Don't know<br>88 Refused
hd3 *(required)*	hd3: In the past two weeks, have you taken any drugs (medication) for diabetes prescribed by a doctor or other health worker?<br>*This refers to oral medication only. Ask the participant to only consider drugs for raised blood glucose prescribed by a doctor or other health worker.*<br>*Question relevant when: ${hd2} = '1'*	1 Yes<br>2 No<br>77 Don't know<br>88 Refused
hd4 *(required)*	hd4: Are you currently taking insulin for diabetes prescribed by a doctor or other health worker?<br>*Ask the participant to only consider insulin that was prescribed by a doctor or other health worker*<br>*Question relevant when: ${hd2} = '1'*	1 Yes<br>2 No<br>77 Don't know<br>88 Refused
hd6 *(required)*	hd6: Have you ever been told by a doctor or other health professional that you have any of the following: prediabetes, impaired fasting glucose, impaired glucose tolerance, borderline diabetes or that your blood sugar is higher than normal but not high enough to be called diabetes or sugar diabetes?<br>*Question relevant when: ${hd2} = '2'*	1 Yes<br>2 No<br>77 Don't know<br>88 Refused
Individual level information > Section 7: Phyiscal measurements<br>*Group relevant when: ${elig_age2} =1 and ${ss10} != '1'*		
Individual level information > Section 7: Phyiscal measurements > Section 7.1: Blood pressure measurement<br>*Group relevant when: ${elig_age2} =1 and ${ss10} != '1'*		
m0 *(required)*	READ:<br><br>First, I would like to measure your blood pressure. For this, I need to place this cuff around your upper arm.<br><br>m0: Participant agreed to have blood pressure measured	1 Yes<br>2 No
m3s *(required)*	m3s: Reading 1: systolic blood pressure<br>*Enter 888 if the respondent refused*<br>*Question relevant when: ${m0} ='1'*	
m3d *(required)*	m3d: Reading 1: diastolic blood pressure<br>*Enter 888 if the respondent refused*<br>*Question relevant when: ${m0} ='1'*	
m4s *(required)*	m4s: Reading 2: systolic blood pressure<br>*Enter 888 if the respondent refused*<br>*Question relevant when: ${m3s} >=140 or ${m3d} >=90*	
m4d *(required)*	m4d: Reading 2: diastolic blood pressure<br>*Enter 888 if the respondent refused*<br>*Question relevant when: ${m3s} >=140 or ${m3d} >=90*	
generated_note_name_63	READ:<br><br>Your blood pressure is [m5s] / [m5d]mmHG.<br><br>You have high blood pressure. Please go to the a clinic for a formal check-up. It is very important to treat high blood pressure to avoid health complications such as a stroke.<br>*Question relevant when: (${m5s} >= 140 or ${m5d} >= 90) and ${hbp3} !='1'*	
generated_note_name_64	READ:<br><br>Your blood pressure is [m5s] / [m5d]mmHG.<br><br>You have high blood pressure despite taking the medication. Please go to a clinic for a check-up and ask the nurse of physician what you can do to lower your blood pressure.<br>*Question relevant when: (${m5s} >= 140 or ${m5d} >= 90) and ${hbp3} ='1'*	
generated_note_name_65	READ:<br><br>Your blood pressure is [m5s] / [m5d]mmHG.<br><br>You have a normal blood pressure. This is good! It makes sense to regularly check your blood pressure to ensure that it continues to be low.<br>*Question relevant when: ${m5s} < 140 and ${m5s} >= 90 and ${m5d} < 90 and ${m5d} >= 60*	

Field	Question	Answer
generated_note_name_66	READ:<br><br>Your blood pressure is [m5s] / [m5d]mmHG.<br><br>This is very low. Please go to the a clinic for a formal check-up.<br>*Question relevant when: ${m5s} < 90 or ${m5d} < 60*	
Individual level information > Section 7: Phyiscal measurements > Section 7.2: Blood glucose measurement<br>*Group relevant when: ${elig_age2} =1 and ${ss10} != '1'*		
m6 *(required)*	READ:<br><br>Now, I would like to measure your blood sugar level. For this, I will need to prick your finger. This does not hurt but only feels a little uncomfortable.<br><br>m6: Participant agreed to have blood glucose measured	1 Yes<br>2 No
fast *(required)*	fast: During the past 12 hours have you had anything to eat or drink other than water?<br>*Question relevant when: ${m6} ='1'*	1 Yes<br>2 No
fbg *(required)*	fbg: Fasting blood glucose reading<br>*Enter 888 if refused*<br>*Question relevant when: ${fast} ='2'*	
fbgelev	READ:<br><br>You have an elevated fasting blood glucose level of [fbg]mmol/L.<br><br>We will conduct a second but different test to confirm the result<br>*Question relevant when: ${fbg} >=5.56 and ${fbg} <888*	
fbgnormal	READ:<br><br>You have an normal fasting blood glucose level of [fbg]mmol/L.<br><br>This is good!<br>*Question relevant when: ${fbg} <5.56*	
m9 *(required)*	m9: HbA1c test result<br>*Enter 888 if refused*<br>*Question relevant when: (${fbg} >=5.56 or ${fast} ='1' or ${hd2} = '1' or ${hd6} = '1') and ${m6} ='1'*	
generated_note_name_77	READ:<br><br>Your blood glucose level is [m9]%.<br><br>You have a elevated blood sugar level. Please go to the a clinic for a formal check-up. It is very important to prevent your blood glucose level from rising further to avoid health complications such as a stroke. The healthcare personnel can give you advice on how to take care of your blood glucose level.<br>*Question relevant when: ${m9} >= 5.7 and ${m9} < 6.5 and ${hd3} !=1 and ${hd4} !=1*	
generated_note_name_78	READ:<br><br>Your blood glucose level is [m9]%.<br><br>You have a high blood sugar level. Please go to the a clinic for a formal check-up. It is very important to treat high blood glucose to avoid health complications such as a stroke.<br>*Question relevant when: ${m9} >= 6.5 and ${m9} < 888 and ${hd3} !=1 and ${hd4} !=1*	
generated_note_name_79	READ:<br><br>Your blood glucose level is [m9]%.<br><br>You have a high blood sugar level despite taking the medication. Please go to a clinic for a check-up and ask the nurse of physician what you can do to lower your blood glucose level.<br>*Question relevant when: ${m9} >= 6.5 and ${m9} < 888 and (${hd3} =1 or ${hd4} =1)*	
generated_note_name_80	READ:<br><br>Your blood glucose level is [m9]%.<br><br>You have a normal blood glucose level. This is good!<br>*Question relevant when: ((${m9} < 5.7 and ${m9} > 3.9 and ${hd3} !=1 and ${hd4} !=1) or (${m9} < 6.5 and ${m9} > 3.9 and (${hd3} =1 or ${hd4} =1)))*	
generated_note_name_81	READ:<br><br>Your blood glucose level is [m9]%.<br><br>You have a very low blood sugar level. You immediately have to drink a sugary drink (fruit juice, soda with sugar) or eat something sweet (honey, sugary candy). You also need to visit a health care clinic for a check-up.<br>*Question relevant when: ${m9} <= 3.9*	
Individual level information > Section 7: Phyiscal measurements > Section 7.3: Height, weight, and waist circumference measurements		

Field	Question	Answer
m14 *(required)*	m14: Height (in cm)<br>*Please enter one decimal digit. If refused, enter 888.8.*	
m16 *(required)*	m16: Weight (in kg)<br>*Please enter one decimal digit. If too large for scale 666.6. If refused, enter 888.8.*	
m19 *(required)*	m19: Waist circumference (in cm)<br>*Please enter one decimal digit. If refused, enter 888.8.*	
elig_ncd_note	This member is eligible for the extended assessment because they have high blood pressure and/or high blood sugar: **[i_ss2]**<br>*Question relevant when: ${elig_htn} =1 or ${elig_dm} =1*	
elig_syn_note	This member is eligible for the extended assessment although they do not have high blood pressure or blood sugar: **[i_ss2]**.<br><br>We will now start the random selection process. If the household members scores 0.75 or more, she will be invited to participate in the full assessment.<br>*Question relevant when: ${elig_syn} =1*	
syn_yes	The score is 0.7150232127993401.<br><br>The respondent is eligible to participate in the syndemics interview (full assessment with respondent who do not have diabetes or hypertension.<br>*Question relevant when: ${syn_sel} =1*	
syn_no	The score is 0.7150232127993401.<br><br>The respondent is not eligible to participate in the full assessment. The interview will be ended now.<br>*Question relevant when: ${syn_sel} =0*	
Extended Individual Interview<br>*Group relevant when: ${elig_htn} =1 or ${elig_dm} =1 or ${syn_sel} =1*		
year *(required)*	year	
month *(required)*	month	
day *(required)*	day	
ext_consent *(required)*	READ:<br><br>You have been selected to participate in the extended assessment. The assessment consists of questions around your health status and health seeking behaviour and you will be offered additional health checks.<br><br>Do you give consent to continue with the extended interview?	1 Yes<br>2 No
Extended Individual Interview > Section 8: Socio-economic information<br>*Group relevant when: ${ext_consent} ='1'*		
ps1 *(required)*	READ:<br><br>Thank you for agreeing to participate in the survey. First, I would like to ask you some general questions.<br><br>ps1: What is your marital status?	1 Married<br>2 Living together<br>3 Divorced<br>4 Separated<br>5 Widowed<br>6 Never married<br>88 Refused
ps3 *(required)*	ps3: What is your origin?<br>*Origin refers to the place of birth.*	1 Swazi<br>2 Non Swazi<br>88 Refused
ps4n *(required)*	ps4: Over the past 12 months, have you stayed in this community for more than 3 months?	1 Yes<br>2 No<br>77 Don't know<br>88 Refused
ps7 *(required)*	ps7: What is the highest level of school you completed?<br>*Question relevant when: ${ps4n} ='1'*	1 None<br>2 Primary<br>3 Secondary<br>4 High School<br>5 Tertiary<br>77 Don't know<br>88 Refused
ps9 *(required)*	ps9: What was the main work activity you were performing over the past 12 months?<br>*Question relevant when: ${ps4n} ='1'*	1 Government employee<br>2 Non-government employee<br>3 Self-employed<br>4 Non-paid<br>5 Student<br>6 Homemaker/housewife<br>7 Retired<br>8 Unemployed (able to work)<br>9 Unemployed (unable to work)<br>99 Refused
Extended Individual Interview > Section 9: History of raised blood pressure		

Field	Question	Answer
Group relevant when: ${ext_consent} ='1' and ${ps4n} =1		
hbp5n *(required)*	READ:<br><br>I will now ask you some questions on health care services for raised blood pressure.<br><br>hbp5: You said before that you have been told by a doctor or healthcare worker that you have high blood pressure/hypertension. Have you been told for the first time in the past 12 months?<br>*Question relevant when: ${hbp2} = '1'*	1 Yes<br>2 No<br>77 Don't know<br>88 Refused
hbp6 *(required)*	hbp6: What is the name of the hospital or clinic where you were told first that you have raised blood pressure or hypertension?<br>*Question relevant when: ${hbp5n} = '1'*	1 Mondi Forest Clinic<br>2 Bulembu Clinic (Havelock)<br>3 Swazico Med (Clinic and Mobile Clinic)<br>4 Maguga Clinic<br>5 Mshingishingini Nazarene Clinic<br>6 Medisun Clinic<br>7 Mangedla Clinic<br>8 Mbabane Public Health Unit<br>9 Ekuphileni Clinic<br>10 S&P Health Care Centre<br>11 Jikani Lambu Medical Center<br>12 Satellite Clinic<br>13 Hhukwini Clinic<br>14 Millsite Clinic<br>15 Mhlambanyatsi Clinic 2<br>16 Ezulwini Clinic (Pharmacy)<br>17 Salvation Army Clinic (Mbabane)<br>18 Siphocosini Clinic<br>19 Ngwenya Wellness Centre<br>20 Nkaba Clinic<br>21 Children's Clinic (Dr Rukundo)<br>22 Ntfonjeni Clinic<br>23 Baphiwe Healthcare and wellness Clinic<br>24 Regina Mundi Clinic / Mondi clinic<br>25 Psychiatric Clinic<br>26 Piggs' Peak Public Health Unit<br>27 Malandzela Nazarene Clinic<br>28 Ekuphileni Medical Clinic - Dr S.P.N Shongwe<br>29 Ngonini Estate Clinic<br>30 Vusweni Clinic<br>31 Ngowane Clinic<br>32 Sigangeni Clinic<br>33 UNISWA Mbabane Campus<br>34 Ekufikeni clinic<br>35 Giving Life Clinic<br>36 Pigg's Peak Nazarene Clinic<br>37 National Baptist Mission Clinic<br>38 Mdzimba UEDF Clinic<br>39 Hhelehhele 11 Clinic<br>40 Herefords Community Clinic<br>41 Mbabane Government Hospital<br>42 The Clinic Group<br>43 Motshane Community Clinic

Field	Question	Answer		
			44	Diabeties Clinic
			45	Mangweni Clinic
			46	Mbabane Correctional Services clinic
			47	Nkoyoyo UEDF Clinic
			48	Ndzingeni Nazarene Clinic
			49	SCU Health Centre
			50	Family Life Clinic (Mbabane)
			51	Nyonyane Clinic
			52	Dvokolwako Health Centre
			53	Family Care Clinic
			54	Childrens Clinic
			55	Emkhuzweni Health Center
			56	Horo Clinic
			57	Bhalekane Nazarene Clinic
			58	University of Limkokwing Clinic
			59	Nkoyoyo Clinic
			60	Dr Eboyens & Partners Clinic
			61	St. Mary's Clinic
			62	Ensingweni Clinic (formerly outreach)
			63	Manzana Clinic (Special Health Care Unit)
			64	Maphalaleni Clinic
			65	Ndvwabangeni Nazarene Clinic
			66	Occupational Theraphy Clinic Mbabane Gov Hospital
			67	Carers Corner Clinic
			68	Mbabane City Council Clinic
			69	Amicall Ngwenya
			70	Nsingizini UEDF Clinic
			71	The Clinic (Mbabane)
			72	Mbuluzi Salvation Army Clinic
			73	Siyanaka Medical Centre
			74	Mbasheni Clinic
			75	Piggs Peak Correctional Services Clinic
			76	Lobamba Clinic
			77	Ntintiza Clinic
			78	Dr Stephens Clinic
			79	Clicks clinics (The Gables outlet)
			80	Pigg's Peak Government Hospital
			81	SOS Children's Village Clinic (Mbabane)
			82	Nkabave Clinic
			83	Clicks clinics (Swazi Plaza Outlet)
			84	Correctional Clinic (Bhalekane)
			85	Bulandzeni Clinic
			86	Mahwalala Red Cross Clinic
			87	Ngwenya Port Health Clinic
			88	Sitsatsaweni Nazerene Clinic

Field	Question	Answer		
			89	Cabrini Ministries Health Care
			90	Mhlume Medical Services
			91	Mambane Clinic
			92	Lubombo Referral
			93	Siphofaneni Clinic
			94	Big Bend Prison Clinic
			95	C.M.C.D Ravenna Clinic
			96	St. Phillip's Clinic
			97	Ubombo Sugar Hospital
			98	Tikhuba Clinic
			99	Lubuli Clinic
			100	Khuphuka Clinic
			101	Manyeveni Nazarene Clinic
			102	Sigcaweni Nazarene Clinic
			103	Siteki Public Health Unit
			104	Mpolonjeni Clinic
			105	Ngwavuma USDF
			106	Flame Clinic (CLOSED)
			107	Matata Clinic
			108	Ikwezi Joy Clinic
			109	C.G.I Clinic
			110	Nkalashane Community Clinic
			111	Mkhaya Clinic-Siteki
			112	Gilgal Clinic
			113	Gucuka Clinic (formerly outreach site)
			114	Tabankulu Estates Clinic
			115	Ngwavuma UEDF Clinic
			116	Mbalenhle Clinic
			117	Tshaneni Clinic
			118	Hlane Clinic
			119	Ebenezer Clinic
			120	Lomahasha Clinic
			121	Good Shepherd Public Health Center
			122	KM III Clinic
			123	New Thulwane Clinic
			124	Mpaka Railway Clinic
			125	Tambuti Estate Clinic
			126	Vuvulane Clinic
			127	SOS Clinic (Ekutfokomeni clinic)
			128	Maloma Colliery Clinic
			129	Sitobela Rural Health Center
			130	UTECH Clinic
			131	Mill Clinic
			132	Sinceni Clinic
			133	Shewula Nazarene Clinic
			134	Anchor Clinic
			135	Tsambokulu Clinic
			136	Dr Martins Clinic
			137	Kudvumisa Foundation
			138	Ndzevane Clinic
			139	Nkonjwa Clinic
			140	Malindza Refugee Camp Clinic
			141	Siphofaneni Private Clinic
			142	Siteki Nazarene Clinic
			143	Mlindazwe UEDF Clinic
			144	Good Shepherd Hospital
			145	Bholi Clinic
			146	Ngomane Clinic
			147	Matsetsa Private Clinic

Field	Question	Answer		
			148	Sikhuphe Airport Clinic
			149	Sibovu Clinic (Mahlangatsha)
			150	Sitsembinkosi Clinic
			151	Mkhulamini Clinic
			152	Teba Clinic-Manzini
			153	Women And Men Health Care Clinic
			154	Clinic 2000 (Dr Mbelu)
			155	Ngonini (OSSU) Clinic
			156	Bhekinkosi Nazarene Clinic
			157	Mahlangatsha Inkhundla
			158	Cana Mission Clinic
			159	Ka-Zondwako Clinic
			160	Family Life Association Clinic (Manzini)
			161	Criminal Lunatic Clinic
			162	Manzini Private Clinic (Imphilo)
			163	Clicks Clinic (Manzini Bhunu Mall)
			164	Kabhudla Clinic
			165	Mawelawela Women Correctional Services Clinic
			166	Leo Garments Clinic
			167	YKK Clinic
			168	National Textile Clinic
			169	Sicalo Health Clinic
			170	Magubheleni Clinic
			171	Nsingizini USDF
			172	RSP Clinic
			173	Mankayane Hospital
			174	Mankayane Public Health Unit
			175	Ancher Clinic
			176	Raleigh Fitkin Memorial Hospital
			177	Luyengo Clinic
			178	Mona Healthlife Clinic
			179	Wellness Center Clinic
			180	Lomgelatshane Clinic (Sidvokodvo)
			181	Kabulin Copporels PTY (LTD) Clinic
			182	Etetsembisweni Clinic
			183	Musi Clinic
			184	Swazican Clinic
			185	Mangcongco Clinic
			186	Mobile Clinic(PPP)Matsapha Town Council
			187	SWAPOL Clinic
			188	Ncabaneni Clinic
			189	Hillside Clinic
			190	Mkhaya Clinic
			191	Phocweni Clinic (UEDF)
			192	Gcina UEDF Clinic
			193	Malkerns Family Life Association
			194	LTD Clinic
			195	Women and Children Hospital
			196	Bulunga Nazarene Clinic
			197	New Village Nazarene Clinic
			198	Mbuluzi UEDF Clinic

Field	Question	Answer

	199	Mankayane Correctional Services Clinic
	200	Lemlandvo Clinic
	201	St. Juliana's Clinic
	202	Heart For Africa-Elrofi Clinic
	203	Mliba Nazarene Clinic
	204	The Luke Commission
	205	Phiwinhlanhla Clinic
	206	Philani Clinic (Manzini)
	207	Union Washing (LTD) Clinic
	208	Litsemba Letfu Men's Clinic
	209	Proton Investment Clinic
	210	Lushikishini Clinic
	211	Emoyeni Clinic
	212	Phumelele Clinic
	213	Sigombeni Red Cross Clinic
	214	Kwaluseni University Clinic
	215	Mother Care Clinic
	216	Sibonginkosi Clinic
	217	Malkerns Juvenile Industrial School Clinic
	218	Sappi Health Centre
	219	Nhlambeni Clinic
	220	Dwalile Clinic
	221	Garrison UEDF Clinic
	222	Giant Clothing Clinic
	223	Philani Clinic (Matsapha)
	224	Ekudzeni Thole Clinic
	225	Mdzimba Clinic USDF
	226	Gcina Bethany Clinic
	227	Texray Clinic
	228	Bethany Clinic
	229	Ngculwini Nazarene Clinic
	230	Bhudla Clinic
	231	Malkerns Clinic USDF
	232	Simply Aid Medical Services
	233	Nonhlanhla Clinic
	234	Lulama Health Clinic
	235	Manzini Government Hospital
	236	NAMPAK Clinic
	237	Mathangeni Church of Christ Clinic
	238	Lamvelase Clinic (Zombodze)
	239	Siphiwo Clinic (formerly Sicelwini)
	240	Psychiatric Hospital (National)
	241	Sigcineni Clinic
	242	RSP VCT
	243	King Sobhuza II Health Unit
	244	Ekuthuleni Clinic
	245	Manzini Health Care (Dr Mathunjwa)
	246	Manzini Town Council
	247	St. Florence Clinic
	248	Kwaluseni Clinic
	249	Shamar Family (shammah) center Clinic
	250	Luve Clinic

Field	Question	Answer		
			251	Nkhabave clinic
			252	Matsapha Unitrans Swaziland Wellness Clinic
			253	Siphosemphilo Clinic (Diabetes)
			254	Ziong Tian Clinic
			255	Bhunya Mill Clinic
			256	Mpuluzi Clinic
			257	Sikhuphe Airport Clinic
			258	Engculwini Clinic
			259	Bhahwini Clinic
			260	Temantungwa Clinic
			261	Women & Men Healthcare Clinic
			262	Lunyengo Student Clinic
			263	Mkhiwa Clinic - Manzini
			264	Mahlanya Clinic Dr L Shongwe
			265	Gebeni Clinic
			266	Dr S Hynd - Manzini Medical Center
			267	Sidvokodvo Railway Clinic
			268	Mliba Nazarene Clinic
			269	St. Theresa's Clinic
			270	Criminal Lunatic Assylum Clinic
			271	KaGogo Mamba Clinic
			272	Mbikwakhe Clinic
			273	Mafutseni Nazerene Clinic
			274	Maloyi Clinic
			275	Homeopathy & Physio Clinic
			276	Manzana Clinic (Special Health Care Unit)
			277	TASC Manzini
			278	Correctional College Staff Clinic
			279	Ngonini Royal Clinic (Special Health Care Unit)
			280	Mhlambanyatsi Clinic
			281	Hlathikhulu Police Wellness Clinic
			282	Baylor Clinic - RFM
			283	Matsanjeni Public Health Unit
			284	Lavumisa Clinic
			285	Nhletsheni Clinic
			286	Casualty Department Hlatikulu Hospital
			287	Dwaleni Clinic
			288	Mbangweni UEDF Clinic
			289	New Haven Clinic
			290	Lavumisa Wellness Clinic
			291	Zheng Yong
			292	Mhlosheni Clinic
			293	Matsanjeni Health Center
			294	Gege Clinic
			295	Hlatikhulu Hospital
			296	Nhlangano Public Health Unit
			297	JCI (Mphelandzaba) Clinic
			298	Our Lady of Sorrows Clinic
			299	Mahlandle Clinic
			300	FTM Clinic
			301	Jericho Clinic
			302	Mkhitsini Clinic
			303	Hluti Clinic

Field	Question	Answer
		304 SOS Clinic (Nhlangano)<br>305 Zombodze Clinic (Shiselweni)<br>306 Phunga Clinic<br>307 Nsalitje Clinic<br>308 Kaphunga Nazarene Clinic<br>309 KaMfishane (KaNdlovu) Clinic<br>310 Nhlangano Health Center<br>311 Nkwene Clinic<br>312 Moti Clinic<br>313 Silele Red Cross Clinic<br>314 Mashobeni Clinic<br>315 Nhlangano Correctional Clinic<br>316 Philani Clinic (Nhlangano)<br>317 Nhlangano H.C Wellness Clinic<br>318 Magubheleni Clinic<br>319 Mgazini Clinic<br>320 Hlatikhulu Public Health Unit<br>321 Bethany Clinic<br>322 Hlathikhulu Wellness Clinic<br>323 Ntshanini Clinic<br>324 Tfokotani Clinic<br>325 Nhlangunjani Clinic<br>326 Luyengo Students Clinic<br>327 Lubombo Police Regional Clinic<br>328 Mbabane Male Wellness Clinic<br>329 Mananga Clinic<br>330 Hhohho Regional Police Clinic<br>331 Ezindwendweni Clinic<br>333 Ezulwini Private Hospital<br>334 South Africa<br>99998 Other, specify
hbp6x *(required)*	hbp6x: Specify name of clinic/hospital of diagnosis<br>*Question relevant when: ${hbp6} = '99998'*	
hbp7 *(required)*	hbp7: Have you ever taken any drugs (medication) for raised blood pressure/hypertension prescribed by a doctor or other health worker?<br>*Question relevant when: ${hbp3} = '2'*	1 Yes<br>2 No<br>77 Don't know<br>88 Refused
hbp8 *(required)*	You said that you are currently taking medication for high blood pressure/hypertension or have done so in the past.<br><br>hbp8: What is the name of hospital/clinic where you initiated high blood pressure/hypertension drug treatment?<br>*Question relevant when: ${hbp3} = '1' or ${hbp7} = '1'*	1 Mondi Forest Clinic<br>2 Bulembu Clinic (Havelock)<br>3 Swazico Med (Clinic and Mobile Clinic)<br>4 Maguga Clinic<br>5 Mshingishingini Nazarene Clinic<br>6 Medisun Clinic<br>7 Mangedla Clinic<br>8 Mbabane Public Health Unit<br>9 Ekuphileni Clinic<br>10 S&P Health Care Centre<br>11 Jikani Lambu Medical Center<br>12 Satellite Clinic<br>13 Hhukwini Clinic<br>14 Millsite Clinic<br>15 Mhlambanyatsi Clinic 2<br>16 Ezulwini Clinic (Pharmacy)

Field	Question	Answer
		17 Salvation Army Clinic (Mbabane)
		18 Siphocosini Clinic
		19 Ngwenya Wellness Centre
		20 Nkaba Clinic
		21 Children's Clinic (Dr Rukundo)
		22 Ntfonjeni Clinic
		23 Baphiwe Healthcare and wellness Clinic
		24 Regina Mundi Clinic / Mondi clinic
		25 Psychiatric Clinic
		26 Piggs' Peak Public Health Unit
		27 Malandzela Nazarene Clinic
		28 Ekuphileni Medical Clinic - Dr S.P.N Shongwe
		29 Ngonini Estate Clinic
		30 Vusweni Clinic
		31 Ngowane Clinic
		32 Sigangeni Clinic
		33 UNISWA Mbabane Campus
		34 Ekufikeni clinic
		35 Giving Life Clinic
		36 Pigg's Peak Nazarene Clinic
		37 National Baptist Mission Clinic
		38 Mdzimba UEDF Clinic
		39 Hhelehhele 11 Clinic
		40 Herefords Community Clinic
		41 Mbabane Government Hospital
		42 The Clinic Group
		43 Motshane Community Clinic
		44 Diabeties Clinic
		45 Mangweni Clinic
		46 Mbabane Correctional Services clinic
		47 Nkoyoyo UEDF Clinic
		48 Ndzingeni Nazarene Clinic
		49 SCU Health Centre
		50 Family Life Clinic (Mbabane)
		51 Nyonyane Clinic
		52 Dvokolwako Health Centre
		53 Family Care Clinic
		54 Childrens Clinic
		55 Emkhuzweni Health Center
		56 Horo Clinic
		57 Bhalekane Nazarene Clinic
		58 University of Limkokwing Clinic
		59 Nkoyoyo Clinic
		60 Dr Eboyens & Partners Clinic
		61 St. Mary's Clinic
		62 Ensingweni Clinic (formerly outreach)

Field	Question	Answer		
			63	Manzana Clinic (Special Health Care Unit)
			64	Maphalaleni Clinic
			65	Ndvwabangeni Nazarene Clinic
			66	Occupational Theraphy Clinic Mbabane Gov Hospital
			67	Carers Corner Clinic
			68	Mbabane City Council Clinic
			69	Amicall Ngwenya
			70	Nsingizini UEDF Clinic
			71	The Clinic (Mbabane)
			72	Mbuluzi Salvation Army Clinic
			73	Siyanaka Medical Centre
			74	Mbasheni Clinic
			75	Piggs Peak Correctional Services Clinic
			76	Lobamba Clinic
			77	Ntintiza Clinic
			78	Dr Stephens Clinic
			79	Clicks clinics (The Gables outlet)
			80	Pigg's Peak Government Hospital
			81	SOS Children's Village Clinic (Mbabane)
			82	Nkabave Clinic
			83	Clicks clinics (Swazi Plaza Outlet)
			84	Correctional Clinic (Bhalekane)
			85	Bulandzeni Clinic
			86	Mahwalala Red Cross Clinic
			87	Ngwenya Port Health Clinic
			88	Sitsatsaweni Nazerene Clinic
			89	Cabrini Ministries Health Care
			90	Mhlume Medical Services
			91	Mambane Clinic
			92	Lubombo Referral
			93	Siphofaneni Clinic
			94	Big Bend Prison Clinic
			95	C.M.C.D Ravenna Clinic
			96	St. Phillip's Clinic
			97	Ubombo Sugar Hospital
			98	Tikhuba Clinic
			99	Lubuli Clinic
			100	Khuphuka Clinic
			101	Manyeveni Nazarene Clinic
			102	Sigcaweni Nazarene Clinic
			103	Siteki Public Health Unit
			104	Mpolonjeni Clinic
			105	Ngwavuma USDF
			106	Flame Clinic (CLOSED)
			107	Matata Clinic
			108	Ikwezi Joy Clinic
			109	C.G.I Clinic
			110	Nkalashane Community Clinic
			111	Mkhaya Clinic-Siteki
			112	Gilgal Clinic

Field	Question	Answer		
			113	Gucuka Clinic (formerly outreach site)
			114	Tabankulu Estates Clinic
			115	Ngwavuma UEDF Clinic
			116	Mbalenhle Clinic
			117	Tshaneni Clinic
			118	Hlane Clinic
			119	Ebenezer Clinic
			120	Lomahasha Clinic
			121	Good Shepherd Public Health Center
			122	KM III Clinic
			123	New Thulwane Clinic
			124	Mpaka Railway Clinic
			125	Tambuti Estate Clinic
			126	Vuvulane Clinic
			127	SOS Clinic (Ekutfokomeni clinic)
			128	Maloma Colliery Clinic
			129	Sitobela Rural Health Center
			130	UTECH Clinic
			131	Mill Clinic
			132	Sinceni Clinic
			133	Shewula Nazarene Clinic
			134	Anchor Clinic
			135	Tsambokulu Clinic
			136	Dr Martins Clinic
			137	Kudvumisa Foundation
			138	Ndzevane Clinic
			139	Nkonjwa Clinic
			140	Malindza Refugee Camp Clinic
			141	Siphofaneni Private Clinic
			142	Siteki Nazarene Clinic
			143	Mlindazwe UEDF Clinic
			144	Good Shepherd Hospital
			145	Bholi Clinic
			146	Ngomane Clinic
			147	Matsetsa Private Clinic
			148	Sikhuphe Airport Clinic
			149	Sibovu Clinic (Mahlangatsha)
			150	Sitsembinkosi Clinic
			151	Mkhulamini Clinic
			152	Teba Clinic-Manzini
			153	Women And Men Health Care Clinic
			154	Clinic 2000 (Dr Mbelu)
			155	Ngonini (OSSU) Clinic
			156	Bhekinkosi Nazarene Clinic
			157	Mahlangatsha Inkhundla
			158	Cana Mission Clinic
			159	Ka-Zondwako Clinic
			160	Family Life Association Clinic (Manzini)
			161	Criminal Lunatic Clinic
			162	Manzini Private Clinic (Imphilo)
			163	Clicks Clinic (Manzini Bhunu Mall)
			164	Kabhudla Clinic
			165	Mawelawela Women Correctional Services Clinic
			166	Leo Garments Clinic
			167	YKK Clinic

Field	Question	Answer

	168	National Textile Clinic
	169	Sicalo Health Clinic
	170	Magubheleni Clinic
	171	Nsingizini USDF
	172	RSP Clinic
	173	Mankayane Hospital
	174	Mankayane Public Health Unit
	175	Ancher Clinic
	176	Raleigh Fitkin Memorial Hospital
	177	Luyengo Clinic
	178	Mona Healthlife Clinic
	179	Wellness Center Clinic
	180	Lomgelatshane Clinic (Sidvokodvo)
	181	Kabulin Copporels PTY (LTD) Clinic
	182	Etetsembisweni Clinic
	183	Musi Clinic
	184	Swazican Clinic
	185	Mangcongco Clinic
	186	Mobile Clinic(PPP)Matsapha Town Council
	187	SWAPOL Clinic
	188	Ncabaneni Clinic
	189	Hillside Clinic
	190	Mkhaya Clinic
	191	Phocweni Clinic (UEDF)
	192	Gcina UEDF Clinic
	193	Malkerns Family Life Association
	194	LTD Clinic
	195	Women and Children Hospital
	196	Bulunga Nazarene Clinic
	197	New Village Nazarene Clinic
	198	Mbuluzi UEDF Clinic
	199	Mankayane Correctional Services Clinic
	200	Lemlandvo Clinic
	201	St. Juliana's Clinic
	202	Heart For Africa-Elrofi Clinic
	203	Mliba Nazarene Clinic
	204	The Luke Commission
	205	Phiwinhlanhla Clinic
	206	Philani Clinic (Manzini)
	207	Union Washing (LTD) Clinic
	208	Litsemba Letfu Men's Clinic
	209	Proton Investment Clinic
	210	Lushikishini Clinic
	211	Emoyeni Clinic
	212	Phumelele Clinic
	213	Sigombeni Red Cross Clinic
	214	Kwaluseni University Clinic
	215	Mother Care Clinic
	216	Sibonginkosi Clinic
	217	Malkerns Juvenile Industrial School Clinic
	218	Sappi Health Centre
	219	Nhlambeni Clinic

Field	Question	Answer		
			220	Dwalile Clinic
			221	Garrison UEDF Clinic
			222	Giant Clothing Clinic
			223	Philani Clinic (Matsapha)
			224	Ekudzeni Thole Clinic
			225	Mdzimba Clinic USDF
			226	Gcina Bethany Clinic
			227	Texray Clinic
			228	Bethany Clinic
			229	Ngculwini Nazarene Clinic
			230	Bhudla Clinic
			231	Malkerns Clinic USDF
			232	Simply Aid Medical Services
			233	Nonhlanhla Clinic
			234	Lulama Health Clinic
			235	Manzini Government Hospital
			236	NAMPAK Clinic
			237	Mathangeni Church of Christ Clinic
			238	Lamvelase Clinic (Zombodze)
			239	Siphiwo Clinic (formerly Sicelwini)
			240	Psychiatric Hospital (National)
			241	Sigcineni Clinic
			242	RSP VCT
			243	King Sobhuza II Health Unit
			244	Ekuthuleni Clinic
			245	Manzini Health Care (Dr Mathunjwa)
			246	Manzini Town Council
			247	St. Florence Clinic
			248	Kwaluseni Clinic
			249	Shamar Family (shammah) center Clinic
			250	Luve Clinic
			251	Nkhabave clinic
			252	Matsapha Unitrans Swaziland Wellness Clinic
			253	Siphosemphilo Clinic (Diabetes)
			254	Ziong Tian Clinic
			255	Bhunya Mill Clinic
			256	Mpuluzi Clinic
			257	Sikhuphe Airport Clinic
			258	Engculwini Clinic
			259	Bhahwini Clinic
			260	Temantungwa Clinic
			261	Women & Men Healthcare Clinic
			262	Lunyengo Student Clinic
			263	Mkhiwa Clinic - Manzini
			264	Mahlanya Clinic Dr L Shongwe
			265	Gebeni Clinic
			266	Dr S Hynd - Manzini Medical Center
			267	Sidvokodvo Railway Clinic
			268	Mliba Nazarene Clinic
			269	St. Theresa's Clinic
			270	Criminal Lunatic Assylum Clinic
			271	KaGogo Mamba Clinic
			272	Mbikwakhe Clinic

Field	Question	Answer

	273	Mafutseni Nazerene Clinic
	274	Maloyi Clinic
	275	Homeopathy & Physio Clinic
	276	Manzana Clinic (Special Health Care Unit)
	277	TASC Manzini
	278	Correctional College Staff Clinic
	279	Ngonini Royal Clinic (Special Health Care Unit)
	280	Mhlambanyatsi Clinic
	281	Hlathikhulu Police Wellness Clinic
	282	Baylor Clinic - RFM
	283	Matsanjeni Public Health Unit
	284	Lavumisa Clinic
	285	Nhletsheni Clinic
	286	Casualty Department Hlatikulu Hospital
	287	Dwaleni Clinic
	288	Mbangweni UEDF Clinic
	289	New Haven Clinic
	290	Lavumisa Wellness Clinic
	291	Zheng Yong
	292	Mhlosheni Clinic
	293	Matsanjeni Health Center
	294	Gege Clinic
	295	Hlatikhulu Hospital
	296	Nhlangano Public Health Unit
	297	JCI (Mphelandzaba) Clinic
	298	Our Lady of Sorrows Clinic
	299	Mahlandle Clinic
	300	FTM Clinic
	301	Jericho Clinic
	302	Mkhitsini Clinic
	303	Hluti Clinic
	304	SOS Clinic (Nhlangano)
	305	Zombodze Clinic (Shiselweni)
	306	Phunga Clinic
	307	Nsalitje Clinic
	308	Kaphunga Nazarene Clinic
	309	KaMfishane (KaNdlovu) Clinic
	310	Nhlangano Health Center
	311	Nkwene Clinic
	312	Moti Clinic
	313	Silele Red Cross Clinic
	314	Mashobeni Clinic
	315	Nhlangano Correctional Clinic
	316	Philani Clinic (Nhlangano)
	317	Nhlangano H.C Wellness Clinic
	318	Magubheleni Clinic
	319	Mgazini Clinic
	320	Hlatikhulu Public Health Unit
	321	Bethany Clinic
	322	Hlathikhulu Wellness Clinic
	323	Ntshanini Clinic
	324	Tfokotani Clinic

Field	Question	Answer
		325 Nhlangunjani Clinic<br>326 Luyengo Students Clinic<br>327 Lubombo Police Regional Clinic<br>328 Mbabane Male Wellness Clinic<br>329 Mananga Clinic<br>330 Hhohho Regional Police Clinic<br>331 Ezindwendweni Clinic<br>333 Ezulwini Private Hospital<br>334 South Africa<br>99998 Other, specify
hbp8x *(required)*	hbp8x: Specify name of clinic/hosptial of drug treatment initiation<br>*Question relevant when: ${hbp8} = '99998'*	
hbp9 *(required)*	hbp9: Do you currently seek care for raised blood pressure/hypertension and if yes what is the name of the clinic/hospital you seek care at?<br>*If not in care search for "not in care".*<br>*Question relevant when: ${hbp2} = '1'*	1 Mondi Forest Clinic<br>2 Bulembu Clinic (Havelock)<br>3 Swazico Med (Clinic and Mobile Clinic)<br>4 Maguga Clinic<br>5 Mshingishingini Nazarene Clinic<br>6 Medisun Clinic<br>7 Mangedla Clinic<br>8 Mbabane Public Health Unit<br>9 Ekuphileni Clinic<br>10 S&P Health Care Centre<br>11 Jikani Lambu Medical Center<br>12 Satellite Clinic<br>13 Hhukwini Clinic<br>14 Millsite Clinic<br>15 Mhlambanyatsi Clinic 2<br>16 Ezulwini Clinic (Pharmacy)<br>17 Salvation Army Clinic (Mbabane)<br>18 Siphocosini Clinic<br>19 Ngwenya Wellness Centre<br>20 Nkaba Clinic<br>21 Children's Clinic (Dr Rukundo)<br>22 Ntfonjeni Clinic<br>23 Baphiwe Healthcare and wellness Clinic<br>24 Regina Mundi Clinic / Mondi clinic<br>25 Psychiatric Clinic<br>26 Piggs' Peak Public Health Unit<br>27 Malandzela Nazarene Clinic<br>28 Ekuphileni Medical Clinic - Dr S.P.N Shongwe<br>29 Ngonini Estate Clinic<br>30 Vusweni Clinic<br>31 Ngowane Clinic<br>32 Sigangeni Clinic<br>33 UNISWA Mbabane Campus<br>34 Ekufikeni clinic<br>35 Giving Life Clinic<br>36 Pigg's Peak Nazarene Clinic<br>37 National Baptist Mission Clinic<br>38 Mdzimba UEDF Clinic

Field	Question	Answer

	39	Hhelehhele 11 Clinic
	40	Herefords Community Clinic
	41	Mbabane Government Hospital
	42	The Clinic Group
	43	Motshane Community Clinic
	44	Diabeties Clinic
	45	Mangweni Clinic
	46	Mbabane Correctional Services clinic
	47	Nkoyoyo UEDF Clinic
	48	Ndzingeni Nazarene Clinic
	49	SCU Health Centre
	50	Family Life Clinic (Mbabane)
	51	Nyonyane Clinic
	52	Dvokolwako Health Centre
	53	Family Care Clinic
	54	Childrens Clinic
	55	Emkhuzweni Health Center
	56	Horo Clinic
	57	Bhalekane Nazarene Clinic
	58	University of Limkokwing Clinic
	59	Nkoyoyo Clinic
	60	Dr Eboyens & Partners Clinic
	61	St. Mary's Clinic
	62	Ensingweni Clinic (formerly outreach)
	63	Manzana Clinic (Special Health Care Unit)
	64	Maphalaleni Clinic
	65	Ndvwabangeni Nazarene Clinic
	66	Occupational Theraphy Clinic Mbabane Gov Hospital
	67	Carers Corner Clinic
	68	Mbabane City Council Clinic
	69	Amicall Ngwenya
	70	Nsingizini UEDF Clinic
	71	The Clinic (Mbabane)
	72	Mbuluzi Salvation Army Clinic
	73	Siyanaka Medical Centre
	74	Mbasheni Clinic
	75	Piggs Peak Correctional Services Clinic
	76	Lobamba Clinic
	77	Ntintiza Clinic
	78	Dr Stephens Clinic
	79	Clicks clinics (The Gables outlet)
	80	Pigg's Peak Government Hospital
	81	SOS Children's Village Clinic (Mbabane)
	82	Nkabave Clinic
	83	Clicks clinics (Swazi Plaza Outlet)

Field	Question	Answer		
			84	Correctional Clinic (Bhalekane)
			85	Bulandzeni Clinic
			86	Mahwalala Red Cross Clinic
			87	Ngwenya Port Health Clinic
			88	Sitsatsaweni Nazerene Clinic
			89	Cabrini Ministries Health Care
			90	Mhlume Medical Services
			91	Mambane Clinic
			92	Lubombo Referral
			93	Siphofaneni Clinic
			94	Big Bend Prison Clinic
			95	C.M.C.D Ravenna Clinic
			96	St. Phillip's Clinic
			97	Ubombo Sugar Hospital
			98	Tikhuba Clinic
			99	Lubuli Clinic
			100	Khuphuka Clinic
			101	Manyeveni Nazarene Clinic
			102	Sigcaweni Nazarene Clinic
			103	Siteki Public Health Unit
			104	Mpolonjeni Clinic
			105	Ngwavuma USDF
			106	Flame Clinic (CLOSED)
			107	Matata Clinic
			108	Ikwezi Joy Clinic
			109	C.G.I Clinic
			110	Nkalashane Community Clinic
			111	Mkhaya Clinic-Siteki
			112	Gilgal Clinic
			113	Gucuka Clinic (formerly outreach site)
			114	Tabankulu Estates Clinic
			115	Ngwavuma UEDF Clinic
			116	Mbalenhle Clinic
			117	Tshaneni Clinic
			118	Hlane Clinic
			119	Ebenezer Clinic
			120	Lomahasha Clinic
			121	Good Shepherd Public Health Center
			122	KM III Clinic
			123	New Thulwane Clinic
			124	Mpaka Railway Clinic
			125	Tambuti Estate Clinic
			126	Vuvulane Clinic
			127	SOS Clinic (Ekutfokomeni clinic)
			128	Maloma Colliery Clinic
			129	Sitobela Rural Health Center
			130	UTECH Clinic
			131	Mill Clinic
			132	Sinceni Clinic
			133	Shewula Nazarene Clinic
			134	Anchor Clinic
			135	Tsambokulu Clinic
			136	Dr Martins Clinic
			137	Kudvumisa Foundation
			138	Ndzevane Clinic
			139	Nkonjwa Clinic

Field	Question	Answer		
			140	Malindza Refugee Camp Clinic
			141	Siphofaneni Private Clinic
			142	Siteki Nazarene Clinic
			143	Mlindazwe UEDF Clinic
			144	Good Shepherd Hospital
			145	Bholi Clinic
			146	Ngomane Clinic
			147	Matsetsa Private Clinic
			148	Sikhuphe Airport Clinic
			149	Sibovu Clinic (Mahlangatsha)
			150	Sitsembinkosi Clinic
			151	Mkhulamini Clinic
			152	Teba Clinic-Manzini
			153	Women And Men Health Care Clinic
			154	Clinic 2000 (Dr Mbelu)
			155	Ngonini (OSSU) Clinic
			156	Bhekinkosi Nazarene Clinic
			157	Mahlangatsha Inkhundla
			158	Cana Mission Clinic
			159	Ka-Zondwako Clinic
			160	Family Life Association Clinic (Manzini)
			161	Criminal Lunatic Clinic
			162	Manzini Private Clinic (Imphilo)
			163	Clicks Clinic (Manzini Bhunu Mall)
			164	Kabhudla Clinic
			165	Mawelawela Women Correctional Services Clinic
			166	Leo Garments Clinic
			167	YKK Clinic
			168	National Textile Clinic
			169	Sicalo Health Clinic
			170	Magubheleni Clinic
			171	Nsingizini USDF
			172	RSP Clinic
			173	Mankayane Hospital
			174	Mankayane Public Health Unit
			175	Ancher Clinic
			176	Raleigh Fitkin Memorial Hospital
			177	Luyengo Clinic
			178	Mona Healthlife Clinic
			179	Wellness Center Clinic
			180	Lomgelatshane Clinic (Sidvokodvo)
			181	Kabulin Copporels PTY (LTD) Clinic
			182	Etetsembisweni Clinic
			183	Musi Clinic
			184	Swazican Clinic
			185	Mangcongco Clinic
			186	Mobile Clinic(PPP)Matsapha Town Council
			187	SWAPOL Clinic
			188	Ncabaneni Clinic
			189	Hillside Clinic
			190	Mkhaya Clinic
			191	Phocweni Clinic (UEDF)
			192	Gcina UEDF Clinic

Field	Question	Answer

	193	Malkerns Family Life Association
	194	LTD Clinic
	195	Women and Children Hospital
	196	Bulunga Nazarene Clinic
	197	New Village Nazarene Clinic
	198	Mbuluzi UEDF Clinic
	199	Mankayane Correctional Services Clinic
	200	Lemlandvo Clinic
	201	St. Juliana's Clinic
	202	Heart For Africa-Elrofi Clinic
	203	Mliba Nazarene Clinic
	204	The Luke Commission
	205	Phiwinhlanhla Clinic
	206	Philani Clinic (Manzini)
	207	Union Washing (LTD) Clinic
	208	Litsemba Letfu Men's Clinic
	209	Proton Investment Clinic
	210	Lushikishini Clinic
	211	Emoyeni Clinic
	212	Phumelele Clinic
	213	Sigombeni Red Cross Clinic
	214	Kwaluseni University Clinic
	215	Mother Care Clinic
	216	Sibonginkosi Clinic
	217	Malkerns Juvenile Industrial School Clinic
	218	Sappi Health Centre
	219	Nhlambeni Clinic
	220	Dwalile Clinic
	221	Garrison UEDF Clinic
	222	Giant Clothing Clinic
	223	Philani Clinic (Matsapha)
	224	Ekudzeni Thole Clinic
	225	Mdzimba Clinic USDF
	226	Gcina Bethany Clinic
	227	Texray Clinic
	228	Bethany Clinic
	229	Ngculwini Nazarene Clinic
	230	Bhudla Clinic
	231	Malkerns Clinic USDF
	232	Simply Aid Medical Services
	233	Nonhlanhla Clinic
	234	Lulama Health Clinic
	235	Manzini Government Hospital
	236	NAMPAK Clinic
	237	Mathangeni Church of Christ Clinic
	238	Lamvelase Clinic (Zombodze)
	239	Siphiwo Clinic (formerly Sicelwini)
	240	Psychiatric Hospital (National)
	241	Sigcineni Clinic
	242	RSP VCT
	243	King Sobhuza II Health Unit

Field	Question	Answer

	244	Ekuthuleni Clinic
	245	Manzini Health Care (Dr Mathunjwa)
	246	Manzini Town Council
	247	St. Florence Clinic
	248	Kwaluseni Clinic
	249	Shamar Family (shammah) center Clinic
	250	Luve Clinic
	251	Nkhabave clinic
	252	Matsapha Unitrans Swaziland Wellness Clinic
	253	Siphosemphilo Clinic (Diabetes)
	254	Ziong Tian Clinic
	255	Bhunya Mill Clinic
	256	Mpuluzi Clinic
	257	Sikhuphe Airport Clinic
	258	Engculwini Clinic
	259	Bhahwini Clinic
	260	Temantungwa Clinic
	261	Women & Men Healthcare Clinic
	262	Lunyengo Student Clinic
	263	Mkhiwa Clinic - Manzini
	264	Mahlanya Clinic Dr L Shongwe
	265	Gebeni Clinic
	266	Dr S Hynd - Manzini Medical Center
	267	Sidvokodvo Railway Clinic
	268	Mliba Nazarene Clinic
	269	St. Theresa's Clinic
	270	Criminal Lunatic Assylum Clinic
	271	KaGogo Mamba Clinic
	272	Mbikwakhe Clinic
	273	Mafutseni Nazerene Clinic
	274	Maloyi Clinic
	275	Homeopathy & Physio Clinic
	276	Manzana Clinic (Special Health Care Unit)
	277	TASC Manzini
	278	Correctional College Staff Clinic
	279	Ngonini Royal Clinic (Special Health Care Unit)
	280	Mhlambanyatsi Clinic
	281	Hlathikhulu Police Wellness Clinic
	282	Baylor Clinic - RFM
	283	Matsanjeni Public Health Unit
	284	Lavumisa Clinic
	285	Nhletsheni Clinic
	286	Casualty Department Hlatikulu Hospital
	287	Dwaleni Clinic
	288	Mbangweni UEDF Clinic
	289	New Haven Clinic
	290	Lavumisa Wellness Clinic
	291	Zheng Yong
	292	Mhlosheni Clinic
	293	Matsanjeni Health Center
	294	Gege Clinic
	295	Hlatikhulu Hospital

Field	Question	Answer
		296 Nhlangano Public Health Unit<br>297 JCI (Mphelandzaba) Clinic<br>298 Our Lady of Sorrows Clinic<br>299 Mahlandle Clinic<br>300 FTM Clinic<br>301 Jericho Clinic<br>302 Mkhitsini Clinic<br>303 Hluti Clinic<br>304 SOS Clinic (Nhlangano)<br>305 Zombodze Clinic (Shiselweni)<br>306 Phunga Clinic<br>307 Nsalitje Clinic<br>308 Kaphunga Nazarene Clinic<br>309 KaMfishane (KaNdlovu) Clinic<br>310 Nhlangano Health Center<br>311 Nkwene Clinic<br>312 Moti Clinic<br>313 Silele Red Cross Clinic<br>314 Mashobeni Clinic<br>315 Nhlangano Correctional Clinic<br>316 Philani Clinic (Nhlangano)<br>317 Nhlangano H.C Wellness Clinic<br>318 Magubheleni Clinic<br>319 Mgazini Clinic<br>320 Hlatikhulu Public Health Unit<br>321 Bethany Clinic<br>322 Hlathikhulu Wellness Clinic<br>323 Ntshanini Clinic<br>324 Tfokotani Clinic<br>325 Nhlangunjani Clinic<br>326 Luyengo Students Clinic<br>327 Lubombo Police Regional Clinic<br>328 Mbabane Male Wellness Clinic<br>329 Mananga Clinic<br>330 Hhohho Regional Police Clinic<br>331 Ezindwendweni Clinic<br>333 Ezulwini Private Hospital<br>334 South Africa<br>99998 Other, specify<br>99999 Not in care
hbp9x *(required)*	hbp9x: Specify name of clinic/hosptial of current care<br>*Question relevant when: ${hbp9} = '99998'*	
hbp10 *(required)*	hbp10: Why are you going to [hbp9_name] now and not to [hbp8_name], i.e. the clinic/hospital where you were initiated?<br>*Do not read out the answer options. Select all that apply.*<br>*Question relevant when: ${hbp8_name} != ${hbp9}_name and ${hbp9} != '99999' and ${hbp9} != ''*	1 New facility is closer to home<br>2 New facility is closer to work<br>3 More services offered<br>4 Better quality of care<br>5 Cheaper services<br>6 The facility was recommended by others (family members, friends, co-workers)<br>7 Advertisements in community<br>8 I moved to a different community in the meantime<br>9 Drugs are more often available at new clinic

Field	Question	Answer
		98 Other, specify
hbp10x *(required)*	hbp10x: Specify other reasons for switching clinic<br>*Question relevant when: selected(${hbp10} , '98')*	
Extended Individual Interview > Section 9: History of raised blood pressure > hclinicfirstvisit<br>*Group relevant when: ${hbp9} != '99999' and ${hbp9} != ''*		
generated_note_name_137	hbp11: When did you first go to [hbp9_name] to get care for raised blood pressure/hypertension?<br>*Insert either month and year of the date (first two fields) or one of the following: years, months, or weeks since first visit. If the respondent does not know, enter 77 in weeks and 88 if the respondent refused to answer.*	
hbp11m	Month of first visit to [hbp9_name] for hypertension care	
hbp11y	Year of first visit to [hbp9_name] for hypertension care<br>*Question relevant when: ${hbp11m} !=''*	
hbp11ys	Years since first visit to [hbp9_name] for hypertension care<br>*Question relevant when: ${hbp11m} ='' and ${hbp11y} =''*	
hbp11ms	Months since first visit to [hbp9_name] for hypertension care<br>*Question relevant when: ${hbp11m} ='' and ${hbp11y} ='' and ${hbp11ys} =''*	
hbp11ws *(required)*	Weeks since first visit to [hbp9_name] for hypertension care<br>*Question relevant when: ${hbp11m} ='' and ${hbp11y} ='' and ${hbp11ys} ='' and ${hbp11ms} =''*	
generated_note_name_144 *(required)*	The date you entered is in the future. Please revise.<br>*Question relevant when: ${hbp11y} = 2022 and ${hbp11m} > ${month}*	
generated_note_name_145 *(required)*	If you enter a year, you also need to enter a month.<br>*Question relevant when: ${hbp11y} != '' and ${hbp11m} =''*	
Extended Individual Interview > Section 9: History of raised blood pressure > bpadv<br>*Group relevant when: ${hbp9} != '99999' and ${hbp9} != ''*		
generated_note_name_148	hbp12: Are you currently receiving any of the following advice for high blood pressure by a doctor or other health worker?	
hbp12	Options	1 Yes<br>2 No<br>77 Don't know<br>88 Refused
hbp12a *(required)*	Quit using tobacco or don't start	1 Yes<br>2 No<br>77 Don't know<br>88 Refused
hbp12b *(required)*	Reduce salt in your diet	1 Yes<br>2 No<br>77 Don't know<br>88 Refused
hbp12c *(required)*	Reduce fat in your diet	1 Yes<br>2 No<br>77 Don't know<br>88 Refused
hbp12d *(required)*	Start or do more physical activity	1 Yes<br>2 No<br>77 Don't know<br>88 Refused
hbp12e *(required)*	Maintain a healthy body weight or lose weight	1 Yes<br>2 No<br>77 Don't know<br>88 Refused
hbp12f *(required)*	Reduce sugary beverages in your diet	1 Yes<br>2 No<br>77 Don't know<br>88 Refused
hbp13 *(required)*	hbp13: In the past 12 months, did you miss any or stop going to the follow up visits for high blood pressure / hypertension care and if yes why?<br>*Question relevant when: ${hbp9} != '99999' and ${hbp9} != ''*	1 Need to work<br>2 Needed to take care of family members<br>3 Too far away from home<br>4 No money to pay for transport<br>5 No money to pay for health care services<br>6 Waiting times are too long<br>7 Low quality of services<br>8 Bad treatment by health care workers<br>9 Feeling uncomfortable during consultation<br>10 No need to go because I felt good

Field	Question	Answer
		11 I forgot about the appointment<br>12 The consultation did not help me to feel better<br>13 I went to a traditional healer instead<br>14 There are no drugs available at the facility<br>15 Did not miss a follow-up visit<br>98 Other, specify
hbp13x *(required)*	hbp13x: Specify other reasons for not going to follow-up visits<br>*Question relevant when: selected(${hbp13} , '98')*	
hbp14 *(required)*	hbp14: What were the reasons for stopping to go to the follow up visits for high blood pressure / hypertension care?<br>*Do not read out the answer options. Select all that apply.*<br>*Question relevant when: ${hbp9} = '99999'*	1 Need to work<br>2 Needed to take care of family members<br>3 Too far away from home<br>4 No money to pay for transport<br>5 No money to pay for health care services<br>6 Waiting times are too long<br>7 Low quality of services<br>8 Bad treatment by health care workers<br>9 Feeling uncomfortable during consultation<br>10 No need to go because I felt good<br>11 I forgot about the appointment<br>12 The consultation did not help me to feel better<br>13 I went to a traditional healer instead<br>14 There are no drugs available at the facility<br>15 My blood pressure went back to normal<br>98 Other, specify
hbp14x *(required)*	hbp14x: Specify other reasons for not going to follow-up visits<br>*Question relevant when: selected(${hbp14} , '98')*	
Extended Individual Interview > Section 10: Participation in DSD models and CDP visits for hypertension care<br>*Group relevant when: ${hbp2} ='1' and ${ext_consent} ='1' and ${ps4n} =1*		
hftc1 *(required)*	READ:<br><br>I will now ask you about different models of delivering care for raised blood pressure or hypertension.<br><br>hftc1: In the past 12 months, have you attended a group meeting based at the facility to obtain care for raised blood pressure or hypertension (Facility Treatment Clubs)?	1 Yes<br>2 No<br>77 Don't know<br>88 Refused
Extended Individual Interview > Section 10: Participation in DSD models and CDP visits for hypertension care > Section 10.1: Facility-based treatment clubs<br>*Group relevant when: ${hftc1} ='1'*		
hftc3 *(required)*	hftc3: Over the past 12 months, how many times did you go to such a group meeting?<br>*If the respondent does not know, enter 77 and 88 if the respondent refused to answer.*	
hftc7 *(required)*	hftc7: In the past 12 months, did you miss any group meetings and if yes why?<br>*Do not read out the answer options. Select all that apply.*	1 I did not have time<br>2 I could not afford transport<br>3 I still had medication<br>4 I did not want to go<br>5 I forgot<br>6 I did not know about the meetings<br>7 I knew no medication was available<br>8 Did not miss a meeting<br>98 Other, specify
hftc7x *(required)*	hftc7x: Specify other reasons for missing FTC meetings<br>*Question relevant when: selected(${hftc7} , '98')*	
hcag1 *(required)*	hcag1: In the past 12 months, have you attended a group meeting based in your community or a community nearby where you take turns in collecting the medication for raised blood pressure or hypertension for all group members and/or self-monitor your blood pressure?	1 Yes<br>2 No<br>77 Don't know<br>88 Refused
Extended Individual Interview > Section 10: Participation in DSD models and CDP visits for hypertension care > Section 10.2: Community advisory groups<br>*Group relevant when: ${hcag1} ='1'*		

Field	Question	Answer
hcag3 *(required)*	hcag3: Over the past 12 months, how many times did you meet with your group?<br>*If the respondent does not know, enter 77 and 88 if the respondent refused to answer.*	
hcag11 *(required)*	hcag11: In the past 12 months, did you miss any group meetings and if yes why?<br>*Do not read out the answer options. Select all that apply.*	1 I did not have time<br>2 I could not afford transport<br>3 I still had medication<br>4 I did not want to go<br>5 I forgot<br>6 I did not know about the meetings<br>7 I knew no medication was available<br>8 Did not miss a meeting<br>98 Other, specify
hcag11x *(required)*	hcag11x: Specify other reasons for missing community group meetings<br>*Question relevant when: selected(${hcag11} , '98')*	
hft1 *(required)*	hft1: In the past 12 months, have you participated in the fast-track model to collect your blood pressure or hypertension medication? The fast track model means that you arrive at the facility and don't have to queue with other clients. Instead, you get preferential treatment and get to see the nurse faster.	1 Yes<br>2 No<br>77 Don't know<br>88 Refused
Extended Individual Interview > Section 10: Participation in DSD models and CDP visits for hypertension care > Section 10.3: Fast-track model<br>*Group relevant when: ${hft1} ='1'*		
hft3 *(required)*	hft3: Over the past 12 months, how often did you go to the facility for care for raised blood pressure or hypertension with preferential treatment?<br>*If the respondent does not know, enter 77 and 88 if the respondent refused to answer.*	
hcdp1 *(required)*	hcdp1: Over the past 12 months, did you visit an outreach session in your community or a community nearby for raised blood pressure or hypertension care, including medication prescription or drug collection?<br>*Question relevant when: ${hbp1} ='1' and ${ext_consent} ='1' and ${ps4n} =1*	1 Yes<br>2 No<br>77 Don't know<br>88 Refused
Extended Individual Interview > Section 10.4: Community Distribution Point<br>*Group relevant when: ${hcdp1} ='1'*		
hcdp3 *(required)*	hcdp3: Over the past 12 months, was your blood pressure measured at an outreach session in your community or a community nearby, where health personnel from clinics provided different health services?	1 Yes<br>2 No<br>77 Don't know<br>88 Refused
hcdp4 *(required)*	hcdp4: Over the past 12 months, did the health personnel at the outreach session refer you to the clinic or hospital for a formal check-up or treatment initiation because of raised blood pressure or hypertension? And if yes, was it for a check-up or treatment initiation or both?<br>*Select all that apply.*	1 Yes, formal check up<br>2 Yes, treatment initiation<br>3 No<br>77 Don't know<br>99 Refused
hcdp6 *(required)*	hcdp6: Over the past 12 months, how many times did you collect your medication for raised blood pressure or hypertension from one of the outreach sessions?<br>*If the respondent does not know, enter 77 and 88 if the respondent refused to answer.*	
Extended Individual Interview > Section 11: Current hypertension medication<br>*Group relevant when: ${hbp3} ='1' and ${ext_consent} ='1' and ${ps4n} =1*		
chm1 *(required)*	READ:<br><br>Now, I will ask you some question about the medication for raised blood pressure or hypertension that you are taking.<br><br>chm1: There are many people who do not take the prescribed dose of their high blood pressure / hypertension medication every day. Has this also happened to you in the past 2 weeks?	1 Yes<br>2 No<br>77 Don't know<br>88 Refused
chm2 *(required)*	chm2: Why did you miss one or more doses over the past 2 weeks?<br>*Question relevant when: ${chm1} ='1'*	1 Drugs were not available at all<br>2 Drugs were available but not for free<br>3 It is hard to remember all the doses / I forget taking them.<br>4 It is hard to pay for this drug<br>5 It is hard to get my refill on time<br>6 I still get unwanted side effects from this drug<br>7 I worry about the long term effects of this drug<br>8 This drug causes other concerns or problems<br>9 I don't feel sick or I don't think I need a drug<br>98 Other, specify
chm2x *(required)*	chm2x: Other reason for missing hypertension dose	

Field	Question	Answer
	Question relevant when: selected(${chm2} , '98')	
chm3 *(required)*	chm3: Of the past 12 months, for how many months did you obtain hypertension drugs?<br>*If the respondent does not know, enter 77 and 88 if the respondent refused to answer.*	
Extended Individual Interview > htrad<br>*Group relevant when: ${hbp2} ='1' and ${ext_consent} ='1' and ${ps4n} =1*		
chm5 *(required)*	chm5: Have you ever seen a traditional healer for raised blood pressure / hypertension?	1 Yes<br>2 No<br>77 Don't know<br>88 Refused
chm6 *(required)*	chm6: Are you currently taking any herbal or traditional remedy for your raised blood pressure / hypertension?	1 Yes<br>2 No<br>77 Don't know<br>88 Refused
Extended Individual Interview > Section 12: History of raised blood sugar<br>*Group relevant when: ${ext_consent} ='1' and ${ps4n} =1*		
hd7bn *(required)*	READ:<br><br>I will now ask you some questions on health care services for raised blood sugar.<br><br>hd7b: You said before that you have been told by a doctor or healthcare worker that you have elevated sugar/diabetes. Have you been told for the first time in the past 12 months?<br>*Question relevant when: ${hd6} = '1'*	1 Yes<br>2 No<br>77 Don't know<br>88 Refused
hd7an *(required)*	READ:<br><br>I will now ask you some questions on health care services for raised blood sugar.<br><br>hd7a: You said before that you have been told by a doctor or healthcare worker that you have high blood sugar/diabetes. Have you been told for the first time in the past 12 months?<br>*Question relevant when: ${hd2} = '1'*	1 Yes<br>2 No<br>77 Don't know<br>88 Refused
hd8a *(required)*	hd8a: What is the name of the hospital or clinic where you told first that you have raised blood sugar or diabetes?<br>*Question relevant when: ${hd7an} = '1'*	1 Mondi Forest Clinic<br>2 Bulembu Clinic (Havelock)<br>3 Swazico Med (Clinic and Mobile Clinic)<br>4 Maguga Clinic<br>5 Mshingishingini Nazarene Clinic<br>6 Medisun Clinic<br>7 Mangedla Clinic<br>8 Mbabane Public Health Unit<br>9 Ekuphileni Clinic<br>10 S&P Health Care Centre<br>11 Jikani Lambu Medical Center<br>12 Satellite Clinic<br>13 Hhukwini Clinic<br>14 Millsite Clinic<br>15 Mhlambanyatsi Clinic 2<br>16 Ezulwini Clinic (Pharmacy)<br>17 Salvation Army Clinic (Mbabane)<br>18 Siphocosini Clinic<br>19 Ngwenya Wellness Centre<br>20 Nkaba Clinic<br>21 Children's Clinic (Dr Rukundo)<br>22 Ntfonjeni Clinic<br>23 Baphiwe Healthcare and wellness Clinic<br>24 Regina Mundi Clinic / Mondi clinic<br>25 Psychiatric Clinic<br>26 Piggs' Peak Public Health Unit<br>27 Malandzela Nazarene Clinic

Field	Question	Answer		
			28	Ekuphileni Medical Clinic - Dr S.P.N Shongwe
			29	Ngonini Estate Clinic
			30	Vusweni Clinic
			31	Ngowane Clinic
			32	Sigangeni Clinic
			33	UNISWA Mbabane Campus
			34	Ekufikeni clinic
			35	Giving Life Clinic
			36	Pigg's Peak Nazarene Clinic
			37	National Baptist Mission Clinic
			38	Mdzimba UEDF Clinic
			39	Hhelehhele 11 Clinic
			40	Herefords Community Clinic
			41	Mbabane Government Hospital
			42	The Clinic Group
			43	Motshane Community Clinic
			44	Diabeties Clinic
			45	Mangweni Clinic
			46	Mbabane Correctional Services clinic
			47	Nkoyoyo UEDF Clinic
			48	Ndzingeni Nazarene Clinic
			49	SCU Health Centre
			50	Family Life Clinic (Mbabane)
			51	Nyonyane Clinic
			52	Dvokolwako Health Centre
			53	Family Care Clinic
			54	Childrens Clinic
			55	Emkhuzweni Health Center
			56	Horo Clinic
			57	Bhalekane Nazarene Clinic
			58	University of Limkokwing Clinic
			59	Nkoyoyo Clinic
			60	Dr Eboyens & Partners Clinic
			61	St. Mary's Clinic
			62	Ensingweni Clinic (formerly outreach)
			63	Manzana Clinic (Special Health Care Unit)
			64	Maphalaleni Clinic
			65	Ndvwabangeni Nazarene Clinic
			66	Occupational Theraphy Clinic Mbabane Gov Hospital
			67	Carers Corner Clinic
			68	Mbabane City Council Clinic
			69	Amicall Ngwenya
			70	Nsingizini UEDF Clinic
			71	The Clinic (Mbabane)
			72	Mbuluzi Salvation Army Clinic
			73	Siyanaka Medical Centre
			74	Mbasheni Clinic

Field	Question	Answer

	75	Piggs Peak Correctional Services Clinic
	76	Lobamba Clinic
	77	Ntintiza Clinic
	78	Dr Stephens Clinic
	79	Clicks clinics (The Gables outlet)
	80	Pigg's Peak Government Hospital
	81	SOS Children's Village Clinic (Mbabane)
	82	Nkabave Clinic
	83	Clicks clinics (Swazi Plaza Outlet)
	84	Correctional Clinic (Bhalekane)
	85	Bulandzeni Clinic
	86	Mahwalala Red Cross Clinic
	87	Ngwenya Port Health Clinic
	88	Sitsatsaweni Nazerene Clinic
	89	Cabrini Ministries Health Care
	90	Mhlume Medical Services
	91	Mambane Clinic
	92	Lubombo Referral
	93	Siphofaneni Clinic
	94	Big Bend Prison Clinic
	95	C.M.C.D Ravenna Clinic
	96	St. Phillip's Clinic
	97	Ubombo Sugar Hospital
	98	Tikhuba Clinic
	99	Lubuli Clinic
	100	Khuphuka Clinic
	101	Manyeveni Nazarene Clinic
	102	Sigcaweni Nazarene Clinic
	103	Siteki Public Health Unit
	104	Mpolonjeni Clinic
	105	Ngwavuma USDF
	106	Flame Clinic (CLOSED)
	107	Matata Clinic
	108	Ikwezi Joy Clinic
	109	C.G.I Clinic
	110	Nkalashane Community Clinic
	111	Mkhaya Clinic-Siteki
	112	Gilgal Clinic
	113	Gucuka Clinic (formerly outreach site)
	114	Tabankulu Estates Clinic
	115	Ngwavuma UEDF Clinic
	116	Mbalenhle Clinic
	117	Tshaneni Clinic
	118	Hlane Clinic
	119	Ebenezer Clinic
	120	Lomahasha Clinic
	121	Good Shepherd Public Health Center
	122	KM III Clinic
	123	New Thulwane Clinic
	124	Mpaka Railway Clinic
	125	Tambuti Estate Clinic
	126	Vuvulane Clinic

Field	Question	Answer		
			127	SOS Clinic (Ekutfokomeni clinic)
			128	Maloma Colliery Clinic
			129	Sitobela Rural Health Center
			130	UTECH Clinic
			131	Mill Clinic
			132	Sinceni Clinic
			133	Shewula Nazarene Clinic
			134	Anchor Clinic
			135	Tsambokulu Clinic
			136	Dr Martins Clinic
			137	Kudvumisa Foundation
			138	Ndzevane Clinic
			139	Nkonjwa Clinic
			140	Malindza Refugee Camp Clinic
			141	Siphofaneni Private Clinic
			142	Siteki Nazarene Clinic
			143	Mlindazwe UEDF Clinic
			144	Good Shepherd Hospital
			145	Bholi Clinic
			146	Ngomane Clinic
			147	Matsetsa Private Clinic
			148	Sikhuphe Airport Clinic
			149	Sibovu Clinic (Mahlangatsha)
			150	Sitsembinkosi Clinic
			151	Mkhulamini Clinic
			152	Teba Clinic-Manzini
			153	Women And Men Health Care Clinic
			154	Clinic 2000 (Dr Mbelu)
			155	Ngonini (OSSU) Clinic
			156	Bhekinkosi Nazarene Clinic
			157	Mahlangatsha Inkhundla
			158	Cana Mission Clinic
			159	Ka-Zondwako Clinic
			160	Family Life Association Clinic (Manzini)
			161	Criminal Lunatic Clinic
			162	Manzini Private Clinic (Imphilo)
			163	Clicks Clinic (Manzini Bhunu Mall)
			164	Kabhudla Clinic
			165	Mawelawela Women Correctional Services Clinic
			166	Leo Garments Clinic
			167	YKK Clinic
			168	National Textile Clinic
			169	Sicalo Health Clinic
			170	Magubheleni Clinic
			171	Nsingizini USDF
			172	RSP Clinic
			173	Mankayane Hospital
			174	Mankayane Public Health Unit
			175	Ancher Clinic
			176	Raleigh Fitkin Memorial Hospital
			177	Luyengo Clinic
			178	Mona Healthlife Clinic
			179	Wellness Center Clinic
			180	Lomgelatshane Clinic (Sidvokodvo)

Field	Question	Answer
		181 Kabulin Copporels PTY (LTD) Clinic<br>182 Etetsembisweni Clinic<br>183 Musi Clinic<br>184 Swazican Clinic<br>185 Mangcongco Clinic<br>186 Mobile Clinic(PPP)Matsapha Town Council<br>187 SWAPOL Clinic<br>188 Ncabaneni Clinic<br>189 Hillside Clinic<br>190 Mkhaya Clinic<br>191 Phocweni Clinic (UEDF)<br>192 Gcina UEDF Clinic<br>193 Malkerns Family Life Association<br>194 LTD Clinic<br>195 Women and Children Hospital<br>196 Bulunga Nazarene Clinic<br>197 New Village Nazarene Clinic<br>198 Mbuluzi UEDF Clinic<br>199 Mankayane Correctional Services Clinic<br>200 Lemlandvo Clinic<br>201 St. Juliana's Clinic<br>202 Heart For Africa-Elrofi Clinic<br>203 Mliba Nazarene Clinic<br>204 The Luke Commission<br>205 Phiwinhlanhla Clinic<br>206 Philani Clinic (Manzini)<br>207 Union Washing (LTD) Clinic<br>208 Litsemba Letfu Men's Clinic<br>209 Proton Investment Clinic<br>210 Lushikishini Clinic<br>211 Emoyeni Clinic<br>212 Phumelele Clinic<br>213 Sigombeni Red Cross Clinic<br>214 Kwaluseni University Clinic<br>215 Mother Care Clinic<br>216 Sibonginkosi Clinic<br>217 Malkerns Juvenile Industrial School Clinic<br>218 Sappi Health Centre<br>219 Nhlambeni Clinic<br>220 Dwalile Clinic<br>221 Garrison UEDF Clinic<br>222 Giant Clothing Clinic<br>223 Philani Clinic (Matsapha)<br>224 Ekudzeni Thole Clinic<br>225 Mdzimba Clinic USDF<br>226 Gcina Bethany Clinic<br>227 Texray Clinic<br>228 Bethany Clinic<br>229 Ngculwini Nazarene Clinic<br>230 Bhudla Clinic<br>231 Malkerns Clinic USDF<br>232 Simply Aid Medical Services<br>233 Nonhlanhla Clinic<br>234 Lulama Health Clinic

Field	Question	Answer		
			235	Manzini Government Hospital
			236	NAMPAK Clinic
			237	Mathangeni Church of Christ Clinic
			238	Lamvelase Clinic (Zombodze)
			239	Siphiwo Clinic (formerly Sicelwini)
			240	Psychiatric Hospital (National)
			241	Sigcineni Clinic
			242	RSP VCT
			243	King Sobhuza II Health Unit
			244	Ekuthuleni Clinic
			245	Manzini Health Care (Dr Mathunjwa)
			246	Manzini Town Council
			247	St. Florence Clinic
			248	Kwaluseni Clinic
			249	Shamar Family (shammah) center Clinic
			250	Luve Clinic
			251	Nkhabave clinic
			252	Matsapha Unitrans Swaziland Wellness Clinic
			253	Siphosemphilo Clinic (Diabetes)
			254	Ziong Tian Clinic
			255	Bhunya Mill Clinic
			256	Mpuluzi Clinic
			257	Sikhuphe Airport Clinic
			258	Engculwini Clinic
			259	Bhahwini Clinic
			260	Temantungwa Clinic
			261	Women & Men Healthcare Clinic
			262	Lunyengo Student Clinic
			263	Mkhiwa Clinic - Manzini
			264	Mahlanya Clinic Dr L Shongwe
			265	Gebeni Clinic
			266	Dr S Hynd - Manzini Medical Center
			267	Sidvokodvo Railway Clinic
			268	Mliba Nazarene Clinic
			269	St. Theresa's Clinic
			270	Criminal Lunatic Assylum Clinic
			271	KaGogo Mamba Clinic
			272	Mbikwakhe Clinic
			273	Mafutseni Nazerene Clinic
			274	Maloyi Clinic
			275	Homeopathy & Physio Clinic
			276	Manzana Clinic (Special Health Care Unit)
			277	TASC Manzini
			278	Correctional College Staff Clinic
			279	Ngonini Royal Clinic (Special Health Care Unit)
			280	Mhlambanyatsi Clinic
			281	Hlathikhulu Police Wellness Clinic
			282	Baylor Clinic - RFM
			283	Matsanjeni Public Health Unit

Field	Question	Answer	
		284	Lavumisa Clinic
		285	Nhletsheni Clinic
		286	Casualty Department Hlatikulu Hospital
		287	Dwaleni Clinic
		288	Mbangweni UEDF Clinic
		289	New Haven Clinic
		290	Lavumisa Wellness Clinic
		291	Zheng Yong
		292	Mhlosheni Clinic
		293	Matsanjeni Health Center
		294	Gege Clinic
		295	Hlatikhulu Hospital
		296	Nhlangano Public Health Unit
		297	JCI (Mphelandzaba) Clinic
		298	Our Lady of Sorrows Clinic
		299	Mahlandle Clinic
		300	FTM Clinic
		301	Jericho Clinic
		302	Mkhitsini Clinic
		303	Hluti Clinic
		304	SOS Clinic (Nhlangano)
		305	Zombodze Clinic (Shiselweni)
		306	Phunga Clinic
		307	Nsalitje Clinic
		308	Kaphunga Nazarene Clinic
		309	KaMfishane (KaNdlovu) Clinic
		310	Nhlangano Health Center
		311	Nkwene Clinic
		312	Moti Clinic
		313	Silele Red Cross Clinic
		314	Mashobeni Clinic
		315	Nhlangano Correctional Clinic
		316	Philani Clinic (Nhlangano)
		317	Nhlangano H.C Wellness Clinic
		318	Magubheleni Clinic
		319	Mgazini Clinic
		320	Hlatikhulu Public Health Unit
		321	Bethany Clinic
		322	Hlathikhulu Wellness Clinic
		323	Ntshanini Clinic
		324	Tfokotani Clinic
		325	Nhlangunjani Clinic
		326	Luyengo Students Clinic
		327	Lubombo Police Regional Clinic
		328	Mbabane Male Wellness Clinic
		329	Mananga Clinic
		330	Hhohho Regional Police Clinic
		331	Ezindwendweni Clinic
		333	Ezulwini Private Hospital
		334	South Africa
		99998	Other, specify
hd8b *(required)*	hd8b: What is the name of the hospital or clinic where you told first that you have elevated blood sugar or pre-diabetes? *Question relevant when: ${hd7bn} = '1'*	1	Mondi Forest Clinic
		2	Bulembu Clinic (Havelock)
		3	Swazico Med (Clinic and Mobile Clinic)

Field	Question	Answer
		4 Maguga Clinic<br>5 Mshingishingini Nazarene Clinic<br>6 Medisun Clinic<br>7 Mangedla Clinic<br>8 Mbabane Public Health Unit<br>9 Ekuphileni Clinic<br>10 S&P Health Care Centre<br>11 Jikani Lambu Medical Center<br>12 Satellite Clinic<br>13 Hhukwini Clinic<br>14 Millsite Clinic<br>15 Mhlambanyatsi Clinic 2<br>16 Ezulwini Clinic (Pharmacy)<br>17 Salvation Army Clinic (Mbabane)<br>18 Siphocosini Clinic<br>19 Ngwenya Wellness Centre<br>20 Nkaba Clinic<br>21 Children's Clinic (Dr Rukundo)<br>22 Ntfonjeni Clinic<br>23 Baphiwe Healthcare and wellness Clinic<br>24 Regina Mundi Clinic / Mondi clinic<br>25 Psychiatric Clinic<br>26 Piggs' Peak Public Health Unit<br>27 Malandzela Nazarene Clinic<br>28 Ekuphileni Medical Clinic - Dr S.P.N Shongwe<br>29 Ngonini Estate Clinic<br>30 Vusweni Clinic<br>31 Ngowane Clinic<br>32 Sigangeni Clinic<br>33 UNISWA Mbabane Campus<br>34 Ekufikeni clinic<br>35 Giving Life Clinic<br>36 Pigg's Peak Nazarene Clinic<br>37 National Baptist Mission Clinic<br>38 Mdzimba UEDF Clinic<br>39 Hhelehhele 11 Clinic<br>40 Herefords Community Clinic<br>41 Mbabane Government Hospital<br>42 The Clinic Group<br>43 Motshane Community Clinic<br>44 Diabeties Clinic<br>45 Mangweni Clinic<br>46 Mbabane Correctional Services clinic<br>47 Nkoyoyo UEDF Clinic<br>48 Ndzingeni Nazarene Clinic<br>49 SCU Health Centre<br>50 Family Life Clinic (Mbabane)<br>51 Nyonyane Clinic

Field	Question	Answer		
			52	Dvokolwako Health Centre
			53	Family Care Clinic
			54	Childrens Clinic
			55	Emkhuzweni Health Center
			56	Horo Clinic
			57	Bhalekane Nazarene Clinic
			58	University of Limkokwing Clinic
			59	Nkoyoyo Clinic
			60	Dr Eboyens & Partners Clinic
			61	St. Mary's Clinic
			62	Ensingweni Clinic (formerly outreach)
			63	Manzana Clinic (Special Health Care Unit)
			64	Maphalaleni Clinic
			65	Ndvwabangeni Nazarene Clinic
			66	Occupational Theraphy Clinic Mbabane Gov Hospital
			67	Carers Corner Clinic
			68	Mbabane City Council Clinic
			69	Amicall Ngwenya
			70	Nsingizini UEDF Clinic
			71	The Clinic (Mbabane)
			72	Mbuluzi Salvation Army Clinic
			73	Siyanaka Medical Centre
			74	Mbasheni Clinic
			75	Piggs Peak Correctional Services Clinic
			76	Lobamba Clinic
			77	Ntintiza Clinic
			78	Dr Stephens Clinic
			79	Clicks clinics (The Gables outlet)
			80	Pigg's Peak Government Hospital
			81	SOS Children's Village Clinic (Mbabane)
			82	Nkabave Clinic
			83	Clicks clinics (Swazi Plaza Outlet)
			84	Correctional Clinic (Bhalekane)
			85	Bulandzeni Clinic
			86	Mahwalala Red Cross Clinic
			87	Ngwenya Port Health Clinic
			88	Sitsatsaweni Nazerene Clinic
			89	Cabrini Ministries Health Care
			90	Mhlume Medical Services
			91	Mambane Clinic
			92	Lubombo Referral
			93	Siphofaneni Clinic
			94	Big Bend Prison Clinic
			95	C.M.C.D Ravenna Clinic
			96	St. Phillip's Clinic
			97	Ubombo Sugar Hospital
			98	Tikhuba Clinic

Field	Question	Answer		
			99	Lubuli Clinic
			100	Khuphuka Clinic
			101	Manyeveni Nazarene Clinic
			102	Sigcaweni Nazarene Clinic
			103	Siteki Public Health Unit
			104	Mpolonjeni Clinic
			105	Ngwavuma USDF
			106	Flame Clinic (CLOSED)
			107	Matata Clinic
			108	Ikwezi Joy Clinic
			109	C.G.I Clinic
			110	Nkalashane Community Clinic
			111	Mkhaya Clinic-Siteki
			112	Gilgal Clinic
			113	Gucuka Clinic (formerly outreach site)
			114	Tabankulu Estates Clinic
			115	Ngwavuma UEDF Clinic
			116	Mbalenhle Clinic
			117	Tshaneni Clinic
			118	Hlane Clinic
			119	Ebenezer Clinic
			120	Lomahasha Clinic
			121	Good Shepherd Public Health Center
			122	KM III Clinic
			123	New Thulwane Clinic
			124	Mpaka Railway Clinic
			125	Tambuti Estate Clinic
			126	Vuvulane Clinic
			127	SOS Clinic (Ekutfokomeni clinic)
			128	Maloma Colliery Clinic
			129	Sitobela Rural Health Center
			130	UTECH Clinic
			131	Mill Clinic
			132	Sinceni Clinic
			133	Shewula Nazarene Clinic
			134	Anchor Clinic
			135	Tsambokulu Clinic
			136	Dr Martins Clinic
			137	Kudvumisa Foundation
			138	Ndzevane Clinic
			139	Nkonjwa Clinic
			140	Malindza Refugee Camp Clinic
			141	Siphofaneni Private Clinic
			142	Siteki Nazarene Clinic
			143	Mlindazwe UEDF Clinic
			144	Good Shepherd Hospital
			145	Bholi Clinic
			146	Ngomane Clinic
			147	Matsetsa Private Clinic
			148	Sikhuphe Airport Clinic
			149	Sibovu Clinic (Mahlangatsha)
			150	Sitsembinkosi Clinic
			151	Mkhulamini Clinic
			152	Teba Clinic-Manzini
			153	Women And Men Health Care Clinic
			154	Clinic 2000 (Dr Mbelu)
			155	Ngonini (OSSU) Clinic

Field	Question	Answer

	156	Bhekinkosi Nazarene Clinic
	157	Mahlangatsha Inkhundla
	158	Cana Mission Clinic
	159	Ka-Zondwako Clinic
	160	Family Life Association Clinic (Manzini)
	161	Criminal Lunatic Clinic
	162	Manzini Private Clinic (Imphilo)
	163	Clicks Clinic (Manzini Bhunu Mall)
	164	Kabhudla Clinic
	165	Mawelawela Women Correctional Services Clinic
	166	Leo Garments Clinic
	167	YKK Clinic
	168	National Textile Clinic
	169	Sicalo Health Clinic
	170	Magubheleni Clinic
	171	Nsingizini USDF
	172	RSP Clinic
	173	Mankayane Hospital
	174	Mankayane Public Health Unit
	175	Ancher Clinic
	176	Raleigh Fitkin Memorial Hospital
	177	Luyengo Clinic
	178	Mona Healthlife Clinic
	179	Wellness Center Clinic
	180	Lomgelatshane Clinic (Sidvokodvo)
	181	Kabulin Copporels PTY (LTD) Clinic
	182	Etetsembisweni Clinic
	183	Musi Clinic
	184	Swazican Clinic
	185	Mangcongco Clinic
	186	Mobile Clinic(PPP)Matsapha Town Council
	187	SWAPOL Clinic
	188	Ncabaneni Clinic
	189	Hillside Clinic
	190	Mkhaya Clinic
	191	Phocweni Clinic (UEDF)
	192	Gcina UEDF Clinic
	193	Malkerns Family Life Association
	194	LTD Clinic
	195	Women and Children Hospital
	196	Bulunga Nazarene Clinic
	197	New Village Nazarene Clinic
	198	Mbuluzi UEDF Clinic
	199	Mankayane Correctional Services Clinic
	200	Lemlandvo Clinic
	201	St. Juliana's Clinic
	202	Heart For Africa-Elrofi Clinic
	203	Mliba Nazarene Clinic
	204	The Luke Commission
	205	Phiwinhlanhla Clinic
	206	Philani Clinic (Manzini)

Field	Question	Answer
		207 Union Washing (LTD) Clinic
		208 Litsemba Letfu Men's Clinic
		209 Proton Investment Clinic
		210 Lushikishini Clinic
		211 Emoyeni Clinic
		212 Phumelele Clinic
		213 Sigombeni Red Cross Clinic
		214 Kwaluseni University Clinic
		215 Mother Care Clinic
		216 Sibonginkosi Clinic
		217 Malkerns Juvenile Industrial School Clinic
		218 Sappi Health Centre
		219 Nhlambeni Clinic
		220 Dwalile Clinic
		221 Garrison UEDF Clinic
		222 Giant Clothing Clinic
		223 Philani Clinic (Matsapha)
		224 Ekudzeni Thole Clinic
		225 Mdzimba Clinic USDF
		226 Gcina Bethany Clinic
		227 Texray Clinic
		228 Bethany Clinic
		229 Ngculwini Nazarene Clinic
		230 Bhudla Clinic
		231 Malkerns Clinic USDF
		232 Simply Aid Medical Services
		233 Nonhlanhla Clinic
		234 Lulama Health Clinic
		235 Manzini Government Hospital
		236 NAMPAK Clinic
		237 Mathangeni Church of Christ Clinic
		238 Lamvelase Clinic (Zombodze)
		239 Siphiwo Clinic (formerly Sicelwini)
		240 Psychiatric Hospital (National)
		241 Sigcineni Clinic
		242 RSP VCT
		243 King Sobhuza II Health Unit
		244 Ekuthuleni Clinic
		245 Manzini Health Care (Dr Mathunjwa)
		246 Manzini Town Council
		247 St. Florence Clinic
		248 Kwaluseni Clinic
		249 Shamar Family (shammah) center Clinic
		250 Luve Clinic
		251 Nkhabave clinic
		252 Matsapha Unitrans Swaziland Wellness Clinic
		253 Siphosemphilo Clinic (Diabetes)
		254 Ziong Tian Clinic
		255 Bhunya Mill Clinic
		256 Mpuluzi Clinic
		257 Sikhuphe Airport Clinic
		258 Engculwini Clinic

Field	Question	Answer		
			259	Bhahwini Clinic
			260	Temantungwa Clinic
			261	Women & Men Healthcare Clinic
			262	Lunyengo Student Clinic
			263	Mkhiwa Clinic - Manzini
			264	Mahlanya Clinic Dr L Shongwe
			265	Gebeni Clinic
			266	Dr S Hynd - Manzini Medical Center
			267	Sidvokodvo Railway Clinic
			268	Mliba Nazarene Clinic
			269	St. Theresa's Clinic
			270	Criminal Lunatic Assylum Clinic
			271	KaGogo Mamba Clinic
			272	Mbikwakhe Clinic
			273	Mafutseni Nazerene Clinic
			274	Maloyi Clinic
			275	Homeopathy & Physio Clinic
			276	Manzana Clinic (Special Health Care Unit)
			277	TASC Manzini
			278	Correctional College Staff Clinic
			279	Ngonini Royal Clinic (Special Health Care Unit)
			280	Mhlambanyatsi Clinic
			281	Hlathikhulu Police Wellness Clinic
			282	Baylor Clinic - RFM
			283	Matsanjeni Public Health Unit
			284	Lavumisa Clinic
			285	Nhletsheni Clinic
			286	Casualty Department Hlatikulu Hospital
			287	Dwaleni Clinic
			288	Mbangweni UEDF Clinic
			289	New Haven Clinic
			290	Lavumisa Wellness Clinic
			291	Zheng Yong
			292	Mhlosheni Clinic
			293	Matsanjeni Health Center
			294	Gege Clinic
			295	Hlatikhulu Hospital
			296	Nhlangano Public Health Unit
			297	JCI (Mphelandzaba) Clinic
			298	Our Lady of Sorrows Clinic
			299	Mahlandle Clinic
			300	FTM Clinic
			301	Jericho Clinic
			302	Mkhitsini Clinic
			303	Hluti Clinic
			304	SOS Clinic (Nhlangano)
			305	Zombodze Clinic (Shiselweni)
			306	Phunga Clinic
			307	Nsalitje Clinic
			308	Kaphunga Nazarene Clinic
			309	KaMfishane (KaNdlovu) Clinic
			310	Nhlangano Health Center

Field	Question	Answer
		311 Nkwene Clinic<br>312 Moti Clinic<br>313 Silele Red Cross Clinic<br>314 Mashobeni Clinic<br>315 Nhlangano Correctional Clinic<br>316 Philani Clinic (Nhlangano)<br>317 Nhlangano H.C Wellness Clinic<br>318 Magubheleni Clinic<br>319 Mgazini Clinic<br>320 Hlatikhulu Public Health Unit<br>321 Bethany Clinic<br>322 Hlathikhulu Wellness Clinic<br>323 Ntshanini Clinic<br>324 Tfokotani Clinic<br>325 Nhlangunjani Clinic<br>326 Luyengo Students Clinic<br>327 Lubombo Police Regional Clinic<br>328 Mbabane Male Wellness Clinic<br>329 Mananga Clinic<br>330 Hhohho Regional Police Clinic<br>331 Ezindwendweni Clinic<br>333 Ezulwini Private Hospital<br>334 South Africa<br>99998 Other, specify
hd8x *(required)*	hd8x: Specify name of clinic/hospital of diagnosis<br>*Question relevant when: ${hd8a} = '99998' or ${hd8b} = '99998'*	
hd9 *(required)*	hd9: Have you ever taken any drugs (oral medication or insulin) for raised blood sugar/diabetes prescribed by a doctor or other health worker?<br>*Question relevant when: ${hd3} = '2' and ${hd4} = '2'*	1 Yes<br>2 No<br>77 Don't know<br>88 Refused
hd10 *(required)*	You said that you are currently taking medication for high blood sugar/diabetes or have done so in the past.<br><br>hd10: What is the name of hospital/clinic where you initiated high blood sugar/diabetes drug treatment?<br>*Question relevant when: ${hd3} = '1' or ${hd4} = '1' or ${hd9} = '1'*	1 Mondi Forest Clinic<br>2 Bulembu Clinic (Havelock)<br>3 Swazico Med (Clinic and Mobile Clinic)<br>4 Maguga Clinic<br>5 Mshingishingini Nazarene Clinic<br>6 Medisun Clinic<br>7 Mangedla Clinic<br>8 Mbabane Public Health Unit<br>9 Ekuphileni Clinic<br>10 S&P Health Care Centre<br>11 Jikani Lambu Medical Center<br>12 Satellite Clinic<br>13 Hhukwini Clinic<br>14 Millsite Clinic<br>15 Mhlambanyatsi Clinic 2<br>16 Ezulwini Clinic (Pharmacy)<br>17 Salvation Army Clinic (Mbabane)<br>18 Siphocosini Clinic<br>19 Ngwenya Wellness Centre<br>20 Nkaba Clinic<br>21 Children's Clinic (Dr Rukundo)<br>22 Ntfonjeni Clinic

Field	Question	Answer		
			23	Baphiwe Healthcare and wellness Clinic
			24	Regina Mundi Clinic / Mondi clinic
			25	Psychiatric Clinic
			26	Piggs' Peak Public Health Unit
			27	Malandzela Nazarene Clinic
			28	Ekuphileni Medical Clinic - Dr S.P.N Shongwe
			29	Ngonini Estate Clinic
			30	Vusweni Clinic
			31	Ngowane Clinic
			32	Sigangeni Clinic
			33	UNISWA Mbabane Campus
			34	Ekufikeni clinic
			35	Giving Life Clinic
			36	Pigg's Peak Nazarene Clinic
			37	National Baptist Mission Clinic
			38	Mdzimba UEDF Clinic
			39	Hhelehhele 11 Clinic
			40	Herefords Community Clinic
			41	Mbabane Government Hospital
			42	The Clinic Group
			43	Motshane Community Clinic
			44	Diabeties Clinic
			45	Mangweni Clinic
			46	Mbabane Correctional Services clinic
			47	Nkoyoyo UEDF Clinic
			48	Ndzingeni Nazarene Clinic
			49	SCU Health Centre
			50	Family Life Clinic (Mbabane)
			51	Nyonyane Clinic
			52	Dvokolwako Health Centre
			53	Family Care Clinic
			54	Childrens Clinic
			55	Emkhuzweni Health Center
			56	Horo Clinic
			57	Bhalekane Nazarene Clinic
			58	University of Limkokwing Clinic
			59	Nkoyoyo Clinic
			60	Dr Eboyens & Partners Clinic
			61	St. Mary's Clinic
			62	Ensingweni Clinic (formerly outreach)
			63	Manzana Clinic (Special Health Care Unit)
			64	Maphalaleni Clinic
			65	Ndvwabangeni Nazarene Clinic
			66	Occupational Theraphy Clinic Mbabane Gov Hospital
			67	Carers Corner Clinic

Field	Question	Answer

	68	Mbabane City Council Clinic
	69	Amicall Ngwenya
	70	Nsingizini UEDF Clinic
	71	The Clinic (Mbabane)
	72	Mbuluzi Salvation Army Clinic
	73	Siyanaka Medical Centre
	74	Mbasheni Clinic
	75	Piggs Peak Correctional Services Clinic
	76	Lobamba Clinic
	77	Ntintiza Clinic
	78	Dr Stephens Clinic
	79	Clicks clinics (The Gables outlet)
	80	Pigg's Peak Government Hospital
	81	SOS Children's Village Clinic (Mbabane)
	82	Nkabave Clinic
	83	Clicks clinics (Swazi Plaza Outlet)
	84	Correctional Clinic (Bhalekane)
	85	Bulandzeni Clinic
	86	Mahwalala Red Cross Clinic
	87	Ngwenya Port Health Clinic
	88	Sitsatsaweni Nazerene Clinic
	89	Cabrini Ministries Health Care
	90	Mhlume Medical Services
	91	Mambane Clinic
	92	Lubombo Referral
	93	Siphofaneni Clinic
	94	Big Bend Prison Clinic
	95	C.M.C.D Ravenna Clinic
	96	St. Phillip's Clinic
	97	Ubombo Sugar Hospital
	98	Tikhuba Clinic
	99	Lubuli Clinic
	100	Khuphuka Clinic
	101	Manyeveni Nazarene Clinic
	102	Sigcaweni Nazarene Clinic
	103	Siteki Public Health Unit
	104	Mpolonjeni Clinic
	105	Ngwavuma USDF
	106	Flame Clinic (CLOSED)
	107	Matata Clinic
	108	Ikwezi Joy Clinic
	109	C.G.I Clinic
	110	Nkalashane Community Clinic
	111	Mkhaya Clinic-Siteki
	112	Gilgal Clinic
	113	Gucuka Clinic (formerly outreach site)
	114	Tabankulu Estates Clinic
	115	Ngwavuma UEDF Clinic
	116	Mbalenhle Clinic
	117	Tshaneni Clinic
	118	Hlane Clinic
	119	Ebenezer Clinic

Field	Question	Answer
		120 Lomahasha Clinic<br>121 Good Shepherd Public Health Center<br>122 KM III Clinic<br>123 New Thulwane Clinic<br>124 Mpaka Railway Clinic<br>125 Tambuti Estate Clinic<br>126 Vuvulane Clinic<br>127 SOS Clinic (Ekutfokomeni clinic)<br>128 Maloma Colliery Clinic<br>129 Sitobela Rural Health Center<br>130 UTECH Clinic<br>131 Mill Clinic<br>132 Sinceni Clinic<br>133 Shewula Nazarene Clinic<br>134 Anchor Clinic<br>135 Tsambokulu Clinic<br>136 Dr Martins Clinic<br>137 Kudvumisa Foundation<br>138 Ndzevane Clinic<br>139 Nkonjwa Clinic<br>140 Malindza Refugee Camp Clinic<br>141 Siphofaneni Private Clinic<br>142 Siteki Nazarene Clinic<br>143 Mlindazwe UEDF Clinic<br>144 Good Shepherd Hospital<br>145 Bholi Clinic<br>146 Ngomane Clinic<br>147 Matsetsa Private Clinic<br>148 Sikhuphe Airport Clinic<br>149 Sibovu Clinic (Mahlangatsha)<br>150 Sitsembinkosi Clinic<br>151 Mkhulamini Clinic<br>152 Teba Clinic-Manzini<br>153 Women And Men Health Care Clinic<br>154 Clinic 2000 (Dr Mbelu)<br>155 Ngonini (OSSU) Clinic<br>156 Bhekinkosi Nazarene Clinic<br>157 Mahlangatsha Inkhundla<br>158 Cana Mission Clinic<br>159 Ka-Zondwako Clinic<br>160 Family Life Association Clinic (Manzini)<br>161 Criminal Lunatic Clinic<br>162 Manzini Private Clinic (Imphilo)<br>163 Clicks Clinic (Manzini Bhunu Mall)<br>164 Kabhudla Clinic<br>165 Mawelawela Women Correctional Services Clinic<br>166 Leo Garments Clinic<br>167 YKK Clinic<br>168 National Textile Clinic<br>169 Sicalo Health Clinic<br>170 Magubheleni Clinic<br>171 Nsingizini USDF<br>172 RSP Clinic<br>173 Mankayane Hospital<br>174 Mankayane Public Health Unit

Field	Question	Answer

	175	Ancher Clinic
	176	Raleigh Fitkin Memorial Hospital
	177	Luyengo Clinic
	178	Mona Healthlife Clinic
	179	Wellness Center Clinic
	180	Lomgelatshane Clinic (Sidvokodvo)
	181	Kabulin Copporels PTY (LTD) Clinic
	182	Etetsembisweni Clinic
	183	Musi Clinic
	184	Swazican Clinic
	185	Mangcongco Clinic
	186	Mobile Clinic(PPP)Matsapha Town Council
	187	SWAPOL Clinic
	188	Ncabaneni Clinic
	189	Hillside Clinic
	190	Mkhaya Clinic
	191	Phocweni Clinic (UEDF)
	192	Gcina UEDF Clinic
	193	Malkerns Family Life Association
	194	LTD Clinic
	195	Women and Children Hospital
	196	Bulunga Nazarene Clinic
	197	New Village Nazarene Clinic
	198	Mbuluzi UEDF Clinic
	199	Mankayane Correctional Services Clinic
	200	Lemlandvo Clinic
	201	St. Juliana's Clinic
	202	Heart For Africa-Elrofi Clinic
	203	Mliba Nazarene Clinic
	204	The Luke Commission
	205	Phiwinhlanhla Clinic
	206	Philani Clinic (Manzini)
	207	Union Washing (LTD) Clinic
	208	Litsemba Letfu Men's Clinic
	209	Proton Investment Clinic
	210	Lushikishini Clinic
	211	Emoyeni Clinic
	212	Phumelele Clinic
	213	Sigombeni Red Cross Clinic
	214	Kwaluseni University Clinic
	215	Mother Care Clinic
	216	Sibonginkosi Clinic
	217	Malkerns Juvenile Industrial School Clinic
	218	Sappi Health Centre
	219	Nhlambeni Clinic
	220	Dwalile Clinic
	221	Garrison UEDF Clinic
	222	Giant Clothing Clinic
	223	Philani Clinic (Matsapha)
	224	Ekudzeni Thole Clinic
	225	Mdzimba Clinic USDF
	226	Gcina Bethany Clinic
	227	Texray Clinic

Field	Question	Answer		
			228	Bethany Clinic
			229	Ngculwini Nazarene Clinic
			230	Bhudla Clinic
			231	Malkerns Clinic USDF
			232	Simply Aid Medical Services
			233	Nonhlanhla Clinic
			234	Lulama Health Clinic
			235	Manzini Government Hospital
			236	NAMPAK Clinic
			237	Mathangeni Church of Christ Clinic
			238	Lamvelase Clinic (Zombodze)
			239	Siphiwo Clinic (formerly Sicelwini)
			240	Psychiatric Hospital (National)
			241	Sigcineni Clinic
			242	RSP VCT
			243	King Sobhuza II Health Unit
			244	Ekuthuleni Clinic
			245	Manzini Health Care (Dr Mathunjwa)
			246	Manzini Town Council
			247	St. Florence Clinic
			248	Kwaluseni Clinic
			249	Shamar Family (shammah) center Clinic
			250	Luve Clinic
			251	Nkhabave clinic
			252	Matsapha Unitrans Swaziland Wellness Clinic
			253	Siphosemphilo Clinic (Diabetes)
			254	Ziong Tian Clinic
			255	Bhunya Mill Clinic
			256	Mpuluzi Clinic
			257	Sikhuphe Airport Clinic
			258	Engculwini Clinic
			259	Bhahwini Clinic
			260	Temantungwa Clinic
			261	Women & Men Healthcare Clinic
			262	Lunyengo Student Clinic
			263	Mkhiwa Clinic - Manzini
			264	Mahlanya Clinic Dr L Shongwe
			265	Gebeni Clinic
			266	Dr S Hynd - Manzini Medical Center
			267	Sidvokodvo Railway Clinic
			268	Mliba Nazarene Clinic
			269	St. Theresa's Clinic
			270	Criminal Lunatic Assylum Clinic
			271	KaGogo Mamba Clinic
			272	Mbikwakhe Clinic
			273	Mafutseni Nazerene Clinic
			274	Maloyi Clinic
			275	Homeopathy & Physio Clinic
			276	Manzana Clinic (Special Health Care Unit)
			277	TASC Manzini
			278	Correctional College Staff Clinic

Field	Question	Answer		
			279	Ngonini Royal Clinic (Special Health Care Unit)
			280	Mhlambanyatsi Clinic
			281	Hlathikhulu Police Wellness Clinic
			282	Baylor Clinic - RFM
			283	Matsanjeni Public Health Unit
			284	Lavumisa Clinic
			285	Nhletsheni Clinic
			286	Casualty Department Hlatikulu Hospital
			287	Dwaleni Clinic
			288	Mbangweni UEDF Clinic
			289	New Haven Clinic
			290	Lavumisa Wellness Clinic
			291	Zheng Yong
			292	Mhlosheni Clinic
			293	Matsanjeni Health Center
			294	Gege Clinic
			295	Hlatikhulu Hospital
			296	Nhlangano Public Health Unit
			297	JCI (Mphelandzaba) Clinic
			298	Our Lady of Sorrows Clinic
			299	Mahlandle Clinic
			300	FTM Clinic
			301	Jericho Clinic
			302	Mkhitsini Clinic
			303	Hluti Clinic
			304	SOS Clinic (Nhlangano)
			305	Zombodze Clinic (Shiselweni)
			306	Phunga Clinic
			307	Nsalitje Clinic
			308	Kaphunga Nazarene Clinic
			309	KaMfishane (KaNdlovu) Clinic
			310	Nhlangano Health Center
			311	Nkwene Clinic
			312	Moti Clinic
			313	Silele Red Cross Clinic
			314	Mashobeni Clinic
			315	Nhlangano Correctional Clinic
			316	Philani Clinic (Nhlangano)
			317	Nhlangano H.C Wellness Clinic
			318	Magubheleni Clinic
			319	Mgazini Clinic
			320	Hlatikhulu Public Health Unit
			321	Bethany Clinic
			322	Hlathikhulu Wellness Clinic
			323	Ntshanini Clinic
			324	Tfokotani Clinic
			325	Nhlangunjani Clinic
			326	Luyengo Students Clinic
			327	Lubombo Police Regional Clinic
			328	Mbabane Male Wellness Clinic
			329	Mananga Clinic
			330	Hhohho Regional Police Clinic

Field	Question	Answer	
hd10x *(required)*	hd10x: Specify name of clinic/hosptial of drug treatment initiation<br>*Question relevant when: ${hd10} = '99998'*	331	Ezindwendweni Clinic
		333	Ezulwini Private Hospital
		334	South Africa
		99998	Other, specify
hd11a *(required)*	hd11a: Do you currently seek care for raised blood sugar/diabetes and if yes what is the name of the clinic/hospital you seek care at?<br>*If not in care search for "not in care".*<br>*Question relevant when: ${hd2} = '1'*	1	Mondi Forest Clinic
		2	Bulembu Clinic (Havelock)
		3	Swazico Med (Clinic and Mobile Clinic)
		4	Maguga Clinic
		5	Mshingishingini Nazarene Clinic
		6	Medisun Clinic
		7	Mangedla Clinic
		8	Mbabane Public Health Unit
		9	Ekuphileni Clinic
		10	S&P Health Care Centre
		11	Jikani Lambu Medical Center
		12	Satellite Clinic
		13	Hhukwini Clinic
		14	Millsite Clinic
		15	Mhlambanyatsi Clinic 2
		16	Ezulwini Clinic (Pharmacy)
		17	Salvation Army Clinic (Mbabane)
		18	Siphocosini Clinic
		19	Ngwenya Wellness Centre
		20	Nkaba Clinic
		21	Children's Clinic (Dr Rukundo)
		22	Ntfonjeni Clinic
		23	Baphiwe Healthcare and wellness Clinic
		24	Regina Mundi Clinic / Mondi clinic
		25	Psychiatric Clinic
		26	Piggs' Peak Public Health Unit
		27	Malandzela Nazarene Clinic
		28	Ekuphileni Medical Clinic - Dr S.P.N Shongwe
		29	Ngonini Estate Clinic
		30	Vusweni Clinic
		31	Ngowane Clinic
		32	Sigangeni Clinic
		33	UNISWA Mbabane Campus
		34	Ekufikeni clinic
		35	Giving Life Clinic
		36	Pigg's Peak Nazarene Clinic
		37	National Baptist Mission Clinic
		38	Mdzimba UEDF Clinic
		39	Hhelehhele 11 Clinic
		40	Herefords Community Clinic
		41	Mbabane Government Hospital
		42	The Clinic Group
		43	Motshane Community Clinic
		44	Diabeties Clinic
		45	Mangweni Clinic
		46	Mbabane Correctional Services clinic
		47	Nkoyoyo UEDF Clinic

Field	Question	Answer
		48 Ndzingeni Nazarene Clinic<br>49 SCU Health Centre<br>50 Family Life Clinic (Mbabane)<br>51 Nyonyane Clinic<br>52 Dvokolwako Health Centre<br>53 Family Care Clinic<br>54 Childrens Clinic<br>55 Emkhuzweni Health Center<br>56 Horo Clinic<br>57 Bhalekane Nazarene Clinic<br>58 University of Limkokwing Clinic<br>59 Nkoyoyo Clinic<br>60 Dr Eboyens & Partners Clinic<br>61 St. Mary's Clinic<br>62 Ensingweni Clinic (formerly outreach)<br>63 Manzana Clinic (Special Health Care Unit)<br>64 Maphalaleni Clinic<br>65 Ndvwabangeni Nazarene Clinic<br>66 Occupational Theraphy Clinic Mbabane Gov Hospital<br>67 Carers Corner Clinic<br>68 Mbabane City Council Clinic<br>69 Amicall Ngwenya<br>70 Nsingizini UEDF Clinic<br>71 The Clinic (Mbabane)<br>72 Mbuluzi Salvation Army Clinic<br>73 Siyanaka Medical Centre<br>74 Mbasheni Clinic<br>75 Piggs Peak Correctional Services Clinic<br>76 Lobamba Clinic<br>77 Ntintiza Clinic<br>78 Dr Stephens Clinic<br>79 Clicks clinics (The Gables outlet)<br>80 Pigg's Peak Government Hospital<br>81 SOS Children's Village Clinic (Mbabane)<br>82 Nkabave Clinic<br>83 Clicks clinics (Swazi Plaza Outlet)<br>84 Correctional Clinic (Bhalekane)<br>85 Bulandzeni Clinic<br>86 Mahwalala Red Cross Clinic<br>87 Ngwenya Port Health Clinic<br>88 Sitsatsaweni Nazerene Clinic<br>89 Cabrini Ministries Health Care<br>90 Mhlume Medical Services<br>91 Mambane Clinic<br>92 Lubombo Referral

Field	Question	Answer		
			93	Siphofaneni Clinic
			94	Big Bend Prison Clinic
			95	C.M.C.D Ravenna Clinic
			96	St. Phillip's Clinic
			97	Ubombo Sugar Hospital
			98	Tikhuba Clinic
			99	Lubuli Clinic
			100	Khuphuka Clinic
			101	Manyeveni Nazarene Clinic
			102	Sigcaweni Nazarene Clinic
			103	Siteki Public Health Unit
			104	Mpolonjeni Clinic
			105	Ngwavuma USDF
			106	Flame Clinic (CLOSED)
			107	Matata Clinic
			108	Ikwezi Joy Clinic
			109	C.G.I Clinic
			110	Nkalashane Community Clinic
			111	Mkhaya Clinic-Siteki
			112	Gilgal Clinic
			113	Gucuka Clinic (formerly outreach site)
			114	Tabankulu Estates Clinic
			115	Ngwavuma UEDF Clinic
			116	Mbalenhle Clinic
			117	Tshaneni Clinic
			118	Hlane Clinic
			119	Ebenezer Clinic
			120	Lomahasha Clinic
			121	Good Shepherd Public Health Center
			122	KM III Clinic
			123	New Thulwane Clinic
			124	Mpaka Railway Clinic
			125	Tambuti Estate Clinic
			126	Vuvulane Clinic
			127	SOS Clinic (Ekutfokomeni clinic)
			128	Maloma Colliery Clinic
			129	Sitobela Rural Health Center
			130	UTECH Clinic
			131	Mill Clinic
			132	Sinceni Clinic
			133	Shewula Nazarene Clinic
			134	Anchor Clinic
			135	Tsambokulu Clinic
			136	Dr Martins Clinic
			137	Kudvumisa Foundation
			138	Ndzevane Clinic
			139	Nkonjwa Clinic
			140	Malindza Refugee Camp Clinic
			141	Siphofaneni Private Clinic
			142	Siteki Nazarene Clinic
			143	Mlindazwe UEDF Clinic
			144	Good Shepherd Hospital
			145	Bholi Clinic
			146	Ngomane Clinic
			147	Matsetsa Private Clinic
			148	Sikhuphe Airport Clinic
			149	Sibovu Clinic (Mahlangatsha)
			150	Sitsembinkosi Clinic
			151	Mkhulamini Clinic

Field	Question	Answer		
			152	Teba Clinic-Manzini
			153	Women And Men Health Care Clinic
			154	Clinic 2000 (Dr Mbelu)
			155	Ngonini (OSSU) Clinic
			156	Bhekinkosi Nazarene Clinic
			157	Mahlangatsha Inkhundla
			158	Cana Mission Clinic
			159	Ka-Zondwako Clinic
			160	Family Life Association Clinic (Manzini)
			161	Criminal Lunatic Clinic
			162	Manzini Private Clinic (Imphilo)
			163	Clicks Clinic (Manzini Bhunu Mall)
			164	Kabhudla Clinic
			165	Mawelawela Women Correctional Services Clinic
			166	Leo Garments Clinic
			167	YKK Clinic
			168	National Textile Clinic
			169	Sicalo Health Clinic
			170	Magubheleni Clinic
			171	Nsingizini USDF
			172	RSP Clinic
			173	Mankayane Hospital
			174	Mankayane Public Health Unit
			175	Ancher Clinic
			176	Raleigh Fitkin Memorial Hospital
			177	Luyengo Clinic
			178	Mona Healthlife Clinic
			179	Wellness Center Clinic
			180	Lomgelatshane Clinic (Sidvokodvo)
			181	Kabulin Copporels PTY (LTD) Clinic
			182	Etetsembisweni Clinic
			183	Musi Clinic
			184	Swazican Clinic
			185	Mangcongco Clinic
			186	Mobile Clinic(PPP)Matsapha Town Council
			187	SWAPOL Clinic
			188	Ncabaneni Clinic
			189	Hillside Clinic
			190	Mkhaya Clinic
			191	Phocweni Clinic (UEDF)
			192	Gcina UEDF Clinic
			193	Malkerns Family Life Association
			194	LTD Clinic
			195	Women and Children Hospital
			196	Bulunga Nazarene Clinic
			197	New Village Nazarene Clinic
			198	Mbuluzi UEDF Clinic
			199	Mankayane Correctional Services Clinic
			200	Lemlandvo Clinic
			201	St. Juliana's Clinic
			202	Heart For Africa-Elrofi Clinic

Field	Question	Answer		
			203	Mliba Nazarene Clinic
			204	The Luke Commission
			205	Phiwinhlanhla Clinic
			206	Philani Clinic (Manzini)
			207	Union Washing (LTD) Clinic
			208	Litsemba Letfu Men's Clinic
			209	Proton Investment Clinic
			210	Lushikishini Clinic
			211	Emoyeni Clinic
			212	Phumelele Clinic
			213	Sigombeni Red Cross Clinic
			214	Kwaluseni University Clinic
			215	Mother Care Clinic
			216	Sibonginkosi Clinic
			217	Malkerns Juvenile Industrial School Clinic
			218	Sappi Health Centre
			219	Nhlambeni Clinic
			220	Dwalile Clinic
			221	Garrison UEDF Clinic
			222	Giant Clothing Clinic
			223	Philani Clinic (Matsapha)
			224	Ekudzeni Thole Clinic
			225	Mdzimba Clinic USDF
			226	Gcina Bethany Clinic
			227	Texray Clinic
			228	Bethany Clinic
			229	Ngculwini Nazarene Clinic
			230	Bhudla Clinic
			231	Malkerns Clinic USDF
			232	Simply Aid Medical Services
			233	Nonhlanhla Clinic
			234	Lulama Health Clinic
			235	Manzini Government Hospital
			236	NAMPAK Clinic
			237	Mathangeni Church of Christ Clinic
			238	Lamvelase Clinic (Zombodze)
			239	Siphiwo Clinic (formerly Sicelwini)
			240	Psychiatric Hospital (National)
			241	Sigcineni Clinic
			242	RSP VCT
			243	King Sobhuza II Health Unit
			244	Ekuthuleni Clinic
			245	Manzini Health Care (Dr Mathunjwa)
			246	Manzini Town Council
			247	St. Florence Clinic
			248	Kwaluseni Clinic
			249	Shamar Family (shammah) center Clinic
			250	Luve Clinic
			251	Nkhabave clinic
			252	Matsapha Unitrans Swaziland Wellness Clinic
			253	Siphosemphilo Clinic (Diabetes)
			254	Ziong Tian Clinic

Field	Question	Answer		
			255	Bhunya Mill Clinic
			256	Mpuluzi Clinic
			257	Sikhuphe Airport Clinic
			258	Engculwini Clinic
			259	Bhahwini Clinic
			260	Temantungwa Clinic
			261	Women & Men Healthcare Clinic
			262	Lunyengo Student Clinic
			263	Mkhiwa Clinic - Manzini
			264	Mahlanya Clinic Dr L Shongwe
			265	Gebeni Clinic
			266	Dr S Hynd - Manzini Medical Center
			267	Sidvokodvo Railway Clinic
			268	Mliba Nazarene Clinic
			269	St. Theresa's Clinic
			270	Criminal Lunatic Assylum Clinic
			271	KaGogo Mamba Clinic
			272	Mbikwakhe Clinic
			273	Mafutseni Nazerene Clinic
			274	Maloyi Clinic
			275	Homeopathy & Physio Clinic
			276	Manzana Clinic (Special Health Care Unit)
			277	TASC Manzini
			278	Correctional College Staff Clinic
			279	Ngonini Royal Clinic (Special Health Care Unit)
			280	Mhlambanyatsi Clinic
			281	Hlathikhulu Police Wellness Clinic
			282	Baylor Clinic - RFM
			283	Matsanjeni Public Health Unit
			284	Lavumisa Clinic
			285	Nhletsheni Clinic
			286	Casualty Department Hlatikulu Hospital
			287	Dwaleni Clinic
			288	Mbangweni UEDF Clinic
			289	New Haven Clinic
			290	Lavumisa Wellness Clinic
			291	Zheng Yong
			292	Mhlosheni Clinic
			293	Matsanjeni Health Center
			294	Gege Clinic
			295	Hlatikhulu Hospital
			296	Nhlangano Public Health Unit
			297	JCI (Mphelandzaba) Clinic
			298	Our Lady of Sorrows Clinic
			299	Mahlandle Clinic
			300	FTM Clinic
			301	Jericho Clinic
			302	Mkhitsini Clinic
			303	Hluti Clinic
			304	SOS Clinic (Nhlangano)
			305	Zombodze Clinic (Shiselweni)
			306	Phunga Clinic
			307	Nsalitje Clinic

Field	Question	Answer	
		308	Kaphunga Nazarene Clinic
		309	KaMfishane (KaNdlovu) Clinic
		310	Nhlangano Health Center
		311	Nkwene Clinic
		312	Moti Clinic
		313	Silele Red Cross Clinic
		314	Mashobeni Clinic
		315	Nhlangano Correctional Clinic
		316	Philani Clinic (Nhlangano)
		317	Nhlangano H.C Wellness Clinic
		318	Magubheleni Clinic
		319	Mgazini Clinic
		320	Hlatikhulu Public Health Unit
		321	Bethany Clinic
		322	Hlathikhulu Wellness Clinic
		323	Ntshanini Clinic
		324	Tfokotani Clinic
		325	Nhlangunjani Clinic
		326	Luyengo Students Clinic
		327	Lubombo Police Regional Clinic
		328	Mbabane Male Wellness Clinic
		329	Mananga Clinic
		330	Hhohho Regional Police Clinic
		331	Ezindwendweni Clinic
		333	Ezulwini Private Hospital
		334	South Africa
		99998	Other, specify
		99999	Not in care
hd11b *(required)*	hd11b: Do you currently seek care for elevated blood sugar/pre-diabetes and if yes what is the name of the clinic/hospital you seek care at?<br>*If not in care search for "not in care".*<br>*Question relevant when: ${hd6} = '1'*	1	Mondi Forest Clinic
		2	Bulembu Clinic (Havelock)
		3	Swazico Med (Clinic and Mobile Clinic)
		4	Maguga Clinic
		5	Mshingishingini Nazarene Clinic
		6	Medisun Clinic
		7	Mangedla Clinic
		8	Mbabane Public Health Unit
		9	Ekuphileni Clinic
		10	S&P Health Care Centre
		11	Jikani Lambu Medical Center
		12	Satellite Clinic
		13	Hhukwini Clinic
		14	Millsite Clinic
		15	Mhlambanyatsi Clinic 2
		16	Ezulwini Clinic (Pharmacy)
		17	Salvation Army Clinic (Mbabane)
		18	Siphocosini Clinic
		19	Ngwenya Wellness Centre
		20	Nkaba Clinic
		21	Children's Clinic (Dr Rukundo)
		22	Ntfonjeni Clinic

Field	Question	Answer		
			23	Baphiwe Healthcare and wellness Clinic
			24	Regina Mundi Clinic / Mondi clinic
			25	Psychiatric Clinic
			26	Piggs' Peak Public Health Unit
			27	Malandzela Nazarene Clinic
			28	Ekuphileni Medical Clinic - Dr S.P.N Shongwe
			29	Ngonini Estate Clinic
			30	Vusweni Clinic
			31	Ngowane Clinic
			32	Sigangeni Clinic
			33	UNISWA Mbabane Campus
			34	Ekufikeni clinic
			35	Giving Life Clinic
			36	Pigg's Peak Nazarene Clinic
			37	National Baptist Mission Clinic
			38	Mdzimba UEDF Clinic
			39	Hhelehhele 11 Clinic
			40	Herefords Community Clinic
			41	Mbabane Government Hospital
			42	The Clinic Group
			43	Motshane Community Clinic
			44	Diabeties Clinic
			45	Mangweni Clinic
			46	Mbabane Correctional Services clinic
			47	Nkoyoyo UEDF Clinic
			48	Ndzingeni Nazarene Clinic
			49	SCU Health Centre
			50	Family Life Clinic (Mbabane)
			51	Nyonyane Clinic
			52	Dvokolwako Health Centre
			53	Family Care Clinic
			54	Childrens Clinic
			55	Emkhuzweni Health Center
			56	Horo Clinic
			57	Bhalekane Nazarene Clinic
			58	University of Limkokwing Clinic
			59	Nkoyoyo Clinic
			60	Dr Eboyens & Partners Clinic
			61	St. Mary's Clinic
			62	Ensingweni Clinic (formerly outreach)
			63	Manzana Clinic (Special Health Care Unit)
			64	Maphalaleni Clinic
			65	Ndvwabangeni Nazarene Clinic
			66	Occupational Theraphy Clinic Mbabane Gov Hospital
			67	Carers Corner Clinic

Field	Question	Answer		
			68	Mbabane City Council Clinic
			69	Amicall Ngwenya
			70	Nsingizini UEDF Clinic
			71	The Clinic (Mbabane)
			72	Mbuluzi Salvation Army Clinic
			73	Siyanaka Medical Centre
			74	Mbasheni Clinic
			75	Piggs Peak Correctional Services Clinic
			76	Lobamba Clinic
			77	Ntintiza Clinic
			78	Dr Stephens Clinic
			79	Clicks clinics (The Gables outlet)
			80	Pigg's Peak Government Hospital
			81	SOS Children's Village Clinic (Mbabane)
			82	Nkabave Clinic
			83	Clicks clinics (Swazi Plaza Outlet)
			84	Correctional Clinic (Bhalekane)
			85	Bulandzeni Clinic
			86	Mahwalala Red Cross Clinic
			87	Ngwenya Port Health Clinic
			88	Sitsatsaweni Nazerene Clinic
			89	Cabrini Ministries Health Care
			90	Mhlume Medical Services
			91	Mambane Clinic
			92	Lubombo Referral
			93	Siphofaneni Clinic
			94	Big Bend Prison Clinic
			95	C.M.C.D Ravenna Clinic
			96	St. Phillip's Clinic
			97	Ubombo Sugar Hospital
			98	Tikhuba Clinic
			99	Lubuli Clinic
			100	Khuphuka Clinic
			101	Manyeveni Nazarene Clinic
			102	Sigcaweni Nazarene Clinic
			103	Siteki Public Health Unit
			104	Mpolonjeni Clinic
			105	Ngwavuma USDF
			106	Flame Clinic (CLOSED)
			107	Matata Clinic
			108	Ikwezi Joy Clinic
			109	C.G.I Clinic
			110	Nkalashane Community Clinic
			111	Mkhaya Clinic-Siteki
			112	Gilgal Clinic
			113	Gucuka Clinic (formerly outreach site)
			114	Tabankulu Estates Clinic
			115	Ngwavuma UEDF Clinic
			116	Mbalenhle Clinic
			117	Tshaneni Clinic
			118	Hlane Clinic
			119	Ebenezer Clinic

Field	Question	Answer		
			120	Lomahasha Clinic
			121	Good Shepherd Public Health Center
			122	KM III Clinic
			123	New Thulwane Clinic
			124	Mpaka Railway Clinic
			125	Tambuti Estate Clinic
			126	Vuvulane Clinic
			127	SOS Clinic (Ekutfokomeni clinic)
			128	Maloma Colliery Clinic
			129	Sitobela Rural Health Center
			130	UTECH Clinic
			131	Mill Clinic
			132	Sinceni Clinic
			133	Shewula Nazarene Clinic
			134	Anchor Clinic
			135	Tsambokulu Clinic
			136	Dr Martins Clinic
			137	Kudvumisa Foundation
			138	Ndzevane Clinic
			139	Nkonjwa Clinic
			140	Malindza Refugee Camp Clinic
			141	Siphofaneni Private Clinic
			142	Siteki Nazarene Clinic
			143	Mlindazwe UEDF Clinic
			144	Good Shepherd Hospital
			145	Bholi Clinic
			146	Ngomane Clinic
			147	Matsetsa Private Clinic
			148	Sikhuphe Airport Clinic
			149	Sibovu Clinic (Mahlangatsha)
			150	Sitsembinkosi Clinic
			151	Mkhulamini Clinic
			152	Teba Clinic-Manzini
			153	Women And Men Health Care Clinic
			154	Clinic 2000 (Dr Mbelu)
			155	Ngonini (OSSU) Clinic
			156	Bhekinkosi Nazarene Clinic
			157	Mahlangatsha Inkhundla
			158	Cana Mission Clinic
			159	Ka-Zondwako Clinic
			160	Family Life Association Clinic (Manzini)
			161	Criminal Lunatic Clinic
			162	Manzini Private Clinic (Imphilo)
			163	Clicks Clinic (Manzini Bhunu Mall)
			164	Kabhudla Clinic
			165	Mawelawela Women Correctional Services Clinic
			166	Leo Garments Clinic
			167	YKK Clinic
			168	National Textile Clinic
			169	Sicalo Health Clinic
			170	Magubheleni Clinic
			171	Nsingizini USDF
			172	RSP Clinic
			173	Mankayane Hospital
			174	Mankayane Public Health Unit

Field	Question	Answer		
			175	Ancher Clinic
			176	Raleigh Fitkin Memorial Hospital
			177	Luyengo Clinic
			178	Mona Healthlife Clinic
			179	Wellness Center Clinic
			180	Lomgelatshane Clinic (Sidvokodvo)
			181	Kabulin Copporels PTY (LTD) Clinic
			182	Etetsembisweni Clinic
			183	Musi Clinic
			184	Swazican Clinic
			185	Mangcongco Clinic
			186	Mobile Clinic(PPP)Matsapha Town Council
			187	SWAPOL Clinic
			188	Ncabaneni Clinic
			189	Hillside Clinic
			190	Mkhaya Clinic
			191	Phocweni Clinic (UEDF)
			192	Gcina UEDF Clinic
			193	Malkerns Family Life Association
			194	LTD Clinic
			195	Women and Children Hospital
			196	Bulunga Nazarene Clinic
			197	New Village Nazarene Clinic
			198	Mbuluzi UEDF Clinic
			199	Mankayane Correctional Services Clinic
			200	Lemlandvo Clinic
			201	St. Juliana's Clinic
			202	Heart For Africa-Elrofi Clinic
			203	Mliba Nazarene Clinic
			204	The Luke Commission
			205	Phiwinhlanhla Clinic
			206	Philani Clinic (Manzini)
			207	Union Washing (LTD) Clinic
			208	Litsemba Letfu Men's Clinic
			209	Proton Investment Clinic
			210	Lushikishini Clinic
			211	Emoyeni Clinic
			212	Phumelele Clinic
			213	Sigombeni Red Cross Clinic
			214	Kwaluseni University Clinic
			215	Mother Care Clinic
			216	Sibonginkosi Clinic
			217	Malkerns Juvenile Industrial School Clinic
			218	Sappi Health Centre
			219	Nhlambeni Clinic
			220	Dwalile Clinic
			221	Garrison UEDF Clinic
			222	Giant Clothing Clinic
			223	Philani Clinic (Matsapha)
			224	Ekudzeni Thole Clinic
			225	Mdzimba Clinic USDF
			226	Gcina Bethany Clinic
			227	Texray Clinic

Field	Question	Answer		
			228	Bethany Clinic
			229	Ngculwini Nazarene Clinic
			230	Bhudla Clinic
			231	Malkerns Clinic USDF
			232	Simply Aid Medical Services
			233	Nonhlanhla Clinic
			234	Lulama Health Clinic
			235	Manzini Government Hospital
			236	NAMPAK Clinic
			237	Mathangeni Church of Christ Clinic
			238	Lamvelase Clinic (Zombodze)
			239	Siphiwo Clinic (formerly Sicelwini)
			240	Psychiatric Hospital (National)
			241	Sigcineni Clinic
			242	RSP VCT
			243	King Sobhuza II Health Unit
			244	Ekuthuleni Clinic
			245	Manzini Health Care (Dr Mathunjwa)
			246	Manzini Town Council
			247	St. Florence Clinic
			248	Kwaluseni Clinic
			249	Shamar Family (shammah) center Clinic
			250	Luve Clinic
			251	Nkhabave clinic
			252	Matsapha Unitrans Swaziland Wellness Clinic
			253	Siphosemphilo Clinic (Diabetes)
			254	Ziong Tian Clinic
			255	Bhunya Mill Clinic
			256	Mpuluzi Clinic
			257	Sikhuphe Airport Clinic
			258	Engculwini Clinic
			259	Bhahwini Clinic
			260	Temantungwa Clinic
			261	Women & Men Healthcare Clinic
			262	Lunyengo Student Clinic
			263	Mkhiwa Clinic - Manzini
			264	Mahlanya Clinic Dr L Shongwe
			265	Gebeni Clinic
			266	Dr S Hynd - Manzini Medical Center
			267	Sidvokodvo Railway Clinic
			268	Mliba Nazarene Clinic
			269	St. Theresa's Clinic
			270	Criminal Lunatic Assylum Clinic
			271	KaGogo Mamba Clinic
			272	Mbikwakhe Clinic
			273	Mafutseni Nazerene Clinic
			274	Maloyi Clinic
			275	Homeopathy & Physio Clinic
			276	Manzana Clinic (Special Health Care Unit)
			277	TASC Manzini
			278	Correctional College Staff Clinic

Field	Question	Answer		
			279	Ngonini Royal Clinic (Special Health Care Unit)
			280	Mhlambanyatsi Clinic
			281	Hlathikhulu Police Wellness Clinic
			282	Baylor Clinic - RFM
			283	Matsanjeni Public Health Unit
			284	Lavumisa Clinic
			285	Nhletsheni Clinic
			286	Casualty Department Hlatikulu Hospital
			287	Dwaleni Clinic
			288	Mbangweni UEDF Clinic
			289	New Haven Clinic
			290	Lavumisa Wellness Clinic
			291	Zheng Yong
			292	Mhlosheni Clinic
			293	Matsanjeni Health Center
			294	Gege Clinic
			295	Hlatikhulu Hospital
			296	Nhlangano Public Health Unit
			297	JCI (Mphelandzaba) Clinic
			298	Our Lady of Sorrows Clinic
			299	Mahlandle Clinic
			300	FTM Clinic
			301	Jericho Clinic
			302	Mkhitsini Clinic
			303	Hluti Clinic
			304	SOS Clinic (Nhlangano)
			305	Zombodze Clinic (Shiselweni)
			306	Phunga Clinic
			307	Nsalitje Clinic
			308	Kaphunga Nazarene Clinic
			309	KaMfishane (KaNdlovu) Clinic
			310	Nhlangano Health Center
			311	Nkwene Clinic
			312	Moti Clinic
			313	Silele Red Cross Clinic
			314	Mashobeni Clinic
			315	Nhlangano Correctional Clinic
			316	Philani Clinic (Nhlangano)
			317	Nhlangano H.C Wellness Clinic
			318	Magubheleni Clinic
			319	Mgazini Clinic
			320	Hlatikhulu Public Health Unit
			321	Bethany Clinic
			322	Hlathikhulu Wellness Clinic
			323	Ntshanini Clinic
			324	Tfokotani Clinic
			325	Nhlangunjani Clinic
			326	Luyengo Students Clinic
			327	Lubombo Police Regional Clinic
			328	Mbabane Male Wellness Clinic
			329	Mananga Clinic
			330	Hhohho Regional Police Clinic

Field	Question	Answer
hd11x *(required)*	hd11x: Specify name of clinic/hosptial of current care<br>*Question relevant when: ${hd11a} = '99998' or ${hd11b} = '99998'*	331 Ezindwendweni Clinic<br>333 Ezulwini Private Hospital<br>334 South Africa<br>99998 Other, specify<br>99999 Not in care
hd12a *(required)*	hd12a: Why are you going to [hd11_name] now and not to [hd10_name], i.e. the clinic/hospital where you were initiated?<br>*Do not read out the answer options. Select all that apply.*<br>*Question relevant when: ${hd11_name} != ${hd10}_name and ${hd11a} != '99999' and ${hd10} != ''*	1 New facility is closer to home<br>2 New facility is closer to work<br>3 More services offered<br>4 Better quality of care<br>5 Cheaper services<br>6 The facility was recommended by others (family members, friends, co-workers)<br>7 Advertisements in community<br>8 I moved to a different community in the meantime<br>9 Drugs are more often available at new clinic<br>98 Other, specify
hd12b *(required)*	hd12b: Why are you going to [hd11_name] now and not to [hd8_name], i.e. the clinic/hospital where you were diagnosed?<br>*Do not read out the answer options. Select all that apply.*<br>*Question relevant when: ${hd11_name} != ${hd8_name} and ${hd11b} != '99999' and ${hd8b} != ''*	1 New facility is closer to home<br>2 New facility is closer to work<br>3 More services offered<br>4 Better quality of care<br>5 Cheaper services<br>6 The facility was recommended by others (family members, friends, co-workers)<br>7 Advertisements in community<br>8 I moved to a different community in the meantime<br>9 Drugs are more often available at new clinic<br>98 Other, specify
hd12x	hd12x: Specify other reasons for switching clinic<br>*Question relevant when: selected(${hd12a} , '98') or selected(${hd12b} , '98')*	
Extended Individual Interview > Section 12: History of raised blood sugar > dclinicfirstvisit<br>*Group relevant when: ${hd11a} != '99999' and ${hd11a} != ''*		
generated_note_name_228	hd13a: When did you first go to [hd11_name] to get care for raised blood sugar/diabetes?<br>*Insert either month and year of the date (first two fields) or one of the following: years, months, or weeks since first visit. If the respondent does not know, enter 77 in weeks and 88 if the respondent refused to answer.*	
hd13am	Month of first visit to [hd11_name] for diabetes care	
hd13ay	Year of first visit to [hd11_name] for diabetes care<br>*Question relevant when: ${hd13am} !=''*	
hd13ays	Year since first visit to [hd11_name] for diabetes care<br>*Question relevant when: ${hd13am} ='' and ${hd13ay} =''*	
hd13ams	Months since first visit to [hd11_name] for diabetes care<br>*Question relevant when: ${hd13am} ='' and ${hd13ay} ='' and ${hd13ays} =''*	
hd13aws *(required)*	Weeks since first visit to [hd11_name] for diabetes care<br>*Question relevant when: ${hd13am} ='' and ${hd13ay} ='' and ${hd13ays} ='' and ${hd13ams} =''*	
generated_note_name_235 *(required)*	The date you entered is in the future. Please revise.<br>*Question relevant when: ${hd13ay} = 2022 and ${hd13am} > ${month}*	
generated_note_name_236 *(required)*	If you enter a year, you also need to enter a month.<br>*Question relevant when: ${hd13ay} != '' and ${hd13am} =''*	
Extended Individual Interview > Section 12: History of raised blood sugar > pdclinicfirstvisit<br>*Group relevant when: ${hd11b} != '99999' and ${hd11b} != ''*		
generated_note_name_239	hd13b: When did you first go to [hd11_name] to get care for elevated blood sugar/pre-diabetes?<br>*Insert either month and year of the date (first two fields) or one of the following: years, months, or weeks since first visit. If the respondent does not know, enter 77 in weeks and 88 if the respondent refused to answer.*	
hd13bm	hd13bm: Month of first visit to [hd11_name] for pre-diabetes care	
hd13by	hd13by: Year of first visit to [hd11_name] for pre-diabetes care<br>*Question relevant when: ${hd13bm} !=''*	
hd13bys	hd13bys: Year since first visit to [hd11_name] for pre-diabetes care<br>*Question relevant when: ${hd13bm} ='' and ${hd13by} =''*	
hd13bms	hd13bms: Months since first visit to [hd11_name] for pre-diabetes care<br>*Question relevant when: ${hd13bm} ='' and ${hd13by} ='' and ${hd13bys} =''*	
hd13bws *(required)*	hd13bws: Weeks since first visit to [hd11_name] for pre-diabetes care<br>*Question relevant when: ${hd13bm} ='' and ${hd13by} ='' and ${hd13bys} ='' and ${hd13bms} =''*	
generated_note_name_246 *(required)*	The date you entered is in the future. Please revise.<br>*Question relevant when: ${hd13by} = 2022 and ${hd13bm} > ${month}*	
generated_note_name_247 *(required)*	If you enter a year, you also need to enter a month.<br>*Question relevant when: ${hd13by} != '' and ${hd13bm} =''*	

Field	Question	Answer
Extended Individual Interview > Section 12: History of raised blood sugar > bgadv<br>*Group relevant when: (${hd11a} != '99999' and ${hd11a} != '') or (${hd11b} != '99999' and ${hd11b} != '')*		
generated_note_name_250	hd14: Are you currently receiving any of the following advice for elevated blood glucose/ pre-diabetes by a doctor or other health worker?<br>*Question relevant when: ${hd11b} != '99999' and ${hd11b} != ''*	
generated_note_name_251	hd14: Are you currently receiving any of the following advice for high blood sugar / diabetes by a doctor or other health worker?<br>*Question relevant when: ${hd11a} != '99999' and ${hd11a} != ''*	
hd14	Options	1 Yes<br>2 No<br>77 Don't know<br>88 Refused
hd14a *(required)*	Quit using tobacco or don't start	1 Yes<br>2 No<br>77 Don't know<br>88 Refused
hd14b *(required)*	Reduce salt in your diet	1 Yes<br>2 No<br>77 Don't know<br>88 Refused
hd14c *(required)*	Reduce fat in your diet	1 Yes<br>2 No<br>77 Don't know<br>88 Refused
hd14d *(required)*	Start or do more physical activity	1 Yes<br>2 No<br>77 Don't know<br>88 Refused
hd14e *(required)*	Maintain a healthy body weight or lose weight	1 Yes<br>2 No<br>77 Don't know<br>88 Refused
hd14f *(required)*	Reduce sugary beverages in your diet	1 Yes<br>2 No<br>77 Don't know<br>88 Refused
hd15 *(required)*	hd15: In the past 12 months, did you miss any or stop going to the follow up visits for high blood sugar / diabetes care and if yes why?<br>*Question relevant when: ${hd11a} != '99999' and ${hd11a} != ''*	1 Need to work<br>2 Needed to take care of family members<br>3 Too far away from home<br>4 No money to pay for transport<br>5 No money to pay for health care services<br>6 Waiting times are too long<br>7 Low quality of services<br>8 Bad treatment by health care workers<br>9 Feeling uncomfortable during consultation<br>10 No need to go because I felt good<br>11 I forgot about the appointment<br>12 The consultation did not help me to feel better<br>13 I went to a traditional healer instead<br>14 There are no drugs available at the facility<br>15 Did not miss a follow-up visit<br>98 Other, specify
hd15x *(required)*	hd15x: Specify other reasons for not going to follow-up visits<br>*Question relevant when: selected(${hd15} , '98')*	
hd16 *(required)*	hd16: What was the reason for stopping to go to the follow up visits for raised blood sugar/diabetes care?<br>*Do not read out the answer options. Select all that apply.*<br>*Question relevant when: ${hd11a} = '99999' or ${hd11b} = '99999'*	1 Need to work<br>2 Needed to take care of family members<br>3 Too far away from home<br>4 No money to pay for transport

Field	Question	Answer
		5 No money to pay for health care services<br>6 Waiting times are too long<br>7 Low quality of services<br>8 Bad treatment by health care workers<br>9 Feeling uncomfortable during consultation<br>10 No need to go because I felt good<br>11 I forgot about the appointment<br>12 The consultation did not help me to feel better<br>13 I went to a traditional healer instead<br>14 There are no drugs available at the facility<br>98 Other, specify
hd16x *(required)*	hd16x: Specify other reasons for not going to follow-up visits<br>*Question relevant when: selected(${hd16} , '98')*	
Extended Individual Interview > Section 13: Participation in DSD models and CDP visits for diabetes care<br>*Group relevant when: ${hd2} ='1' and ${ext_consent} ='1' and ${ps4n} =1*		
dftc1 *(required)*	READ:<br><br>I will now ask you about different models of delivering care for raised blood sugar or diabetes.<br><br>dftc1: In the past 12 months, have you attended a group meeting based at the facility to obtain care for raised blood sugar or diabetes (Facility Treatment Clubs)?	1 Yes<br>2 No<br>77 Don't know<br>88 Refused
Extended Individual Interview > Section 13: Participation in DSD models and CDP visits for diabetes care > Section 13.1: Facility-based treatment clubs<br>*Group relevant when: ${dftc1} ='1' and ${hftc1} !='1'*		
dftc3 *(required)*	dftc3: Over the past 12 months, how many times did you go to such a group meeting?<br>*If the respondent does not know, enter 77 and 88 if the respondent refused to answer.*	
dftc7 *(required)*	dftc7: In the past 12 months, did you miss any group meetings and if yes why?<br>*Do not read out the answer options. Select all that apply.*	1 I did not have time<br>2 I could not afford transport<br>3 I still had medication<br>4 I did not want to go<br>5 I forgot<br>6 I did not know about the meetings<br>7 I knew no medication was available<br>8 Did not miss a meeting<br>98 Other, specify
dftc7x *(required)*	dftc7x: Specify other reasons for missing FTC meetings<br>*Question relevant when: selected(${dftc7} , '98')*	
dcag1 *(required)*	dcag1: In the past 12 months, have you attended a group meeting based in your community or a community nearby where you take turns in collecting the high blood sugar or diabetes medication and/or self-monitor your blood sugar?	1 Yes<br>2 No<br>77 Don't know<br>88 Refused
Extended Individual Interview > Section 13: Participation in DSD models and CDP visits for diabetes care > Section 13.2: Community advisory groups<br>*Group relevant when: ${dcag1} ='1' and ${hcag1} !='1'*		
dcag3 *(required)*	dcag3: Over the past 12 months, how many times did you meet with your group?<br>*If the respondent does not know, enter 77 and 88 if the respondent refused to answer.*	
dcag11 *(required)*	dcag11: In the past 12 months, did you miss any group meetings and if yes why?<br>*Do not read out the answer options. Select all that apply.*	1 I did not have time<br>2 I could not afford transport<br>3 I still had medication<br>4 I did not want to go<br>5 I forgot<br>6 I did not know about the meetings<br>7 I knew no medication was available<br>8 Did not miss a meeting<br>98 Other, specify
dcag11x *(required)*	dcag11x: Specify other reasons for missing community group meetings<br>*Question relevant when: selected(${dcag11} , '98')*	
dft1 *(required)*	dft1: In the past 12 months, have you participated in the fast-track model to collect your blood sugar or diabetesmedication? The fast track model means that you arrive at the facility and don't have to queue with other clients. Instead, you get preferential treatment and get to see the nurse faster.	1 Yes<br>2 No<br>77 Don't know

Field	Question	Answer
		88 Refused
Extended Individual Interview > Section 13: Participation in DSD models and CDP visits for diabetes care > Section 13.3: Fast-track model<br>*Group relevant when: ${dft1} ='1' and ${hft1} !='1'*		
dft3 *(required)*	dft3: Over the past 12 months, how often did you go to the facility for care for raised blood sugar or diabetes with preferential treatment?<br>*If the respondent does not know, enter 77 and 88 if the respondent refused to answer.*	
dcdp1 *(required)*	dcdp1: Over the past 12 months, did you visit an outreach session in your community or a community nearby for raised blood sugar or diabetes care, including medication prescription or drug collection?<br>*Question relevant when: ${hd1} ='1' and ${ext_consent} ='1' and ${ps4n} =1*	1 Yes<br>2 No<br>77 Don't know<br>88 Refused
Extended Individual Interview > Section 13.4: Community Distribution Point<br>*Group relevant when: ${dcdp1} ='1'*		
dcdp3 *(required)*	dcdp3: Over the past 12 months, was your blood sugar measured at an outreach session in your community or a community nearby, where health personnel from clinics provided different health services?<br>*The BG measurement should have been done by a doctor or other health personnel.*	1 Yes<br>2 No<br>77 Don't know<br>88 Refused
dcdp4 *(required)*	dcdp4: Over the past 12 months, did the health personnel at the outreach session refer you to the clinic or hospital for a formal check-up or treatment initiation because of raised blood sugar or diabetes? And if yes, was it for a check-up or treatment initiation or both?<br>*Select all that apply.*	1 Yes, formal check up<br>2 Yes, treatment initiation<br>3 No<br>77 Don't know<br>99 Refused
dcdp6 *(required)*	dcdp6: Over the past 12 months, how many times did you collect your medication for raised blood sugar or diabetes from one of the outreach sessions?<br>*If the respondent does not know, enter 77 and 88 if the respondent refused to answer.*	
Extended Individual Interview > Section 14: Current diabetes medication<br>*Group relevant when: (${hd3} = '1' or ${hd4} = '1') and ${ext_consent} ='1' and ${ps4n} =1*		
cdm1 *(required)*	READ:<br><br>Now, I will ask you some question about the medication for raised blood sugar or diabetes that you are taking.<br><br>cdm1: There are many people who do not take the prescribed dose of their high blood sugar / diabetes medication every day. Has this also happened to you in the past 2 weeks?	1 Yes<br>2 No<br>77 Don't know<br>88 Refused
cdm2 *(required)*	cdm2: Why did you miss one or more doses over the past 2 weeks?<br>*Question relevant when: ${cdm1} ='1'*	1 Drugs were not available at all<br>2 Drugs were available but not for free<br>3 It is hard to remember all the doses / I forget taking them.<br>4 It is hard to pay for this drug<br>5 It is hard to get my refill on time<br>6 I still get unwanted side effects from this drug<br>7 I worry about the long term effects of this drug<br>8 This drug causes other concerns or problems<br>9 I don't feel sick or I don't think I need a drug<br>98 Other, specify
cdm2x *(required)*	cdm2x: Other reason for missing diabetes dose<br>*Question relevant when: selected(${cdm2} , '98')*	
cdm3 *(required)*	cdm3: Of the past 12 months, for how many months did you obtain diabetes drugs?<br>*If the respondent does not know, enter 77 and 88 if the respondent refused to answer.*	
Extended Individual Interview > dtrad<br>*Group relevant when: (${hd2} = '1' or ${hd6} = '1') and ${ext_consent} ='1' and ${ps4n} =1*		
cdm5 *(required)*	cdm5: Have you ever seen a traditional healer for raised blood sugar / diabetes?	1 Yes<br>2 No<br>77 Don't know<br>88 Refused
cdm6 *(required)*	cdm6: Are you currently taking any herbal or traditional remedy for your raised blood sugar / diabetes?	1 Yes<br>2 No<br>77 Don't know<br>88 Refused
Extended Individual Interview > Section 15: Heart attack<br>*Group relevant when: ${ext_consent} ='1' and ${ps4n} =1*		
ha2 *(required)*	ha2: In the past 12 months, did you have a heart attack or chest pain from heart disease (angina) or a stroke (cerebrovascular accident or incident)?<br>*Only count events that were diagnosed by a doctor or other trained health worker.*	1 Yes<br>2 No<br>77 Don't know<br>88 Refused

Field	Question	Answer
Extended Individual Interview > Section 16: Rural Health Motivator<br>*Group relevant when: ${ext_consent} ='1' and ${ps4n} =1*		
generated_note_name_311	READ:<br><br>I would now like to ask you a few questions about Rural Health Motivators. Please bear in mind that all your answers are treated highly confidential. No one will be told about any answers you gave.<br><br>A Rural Health Motivator is someone from the community who regularly visits households. Rural Health Motivators provide information on how to stay healthy and help care for ill people at their home.<br><br>All answers will be treated confidentially and the RHM will not be informed about your responses. Furthermore, it will not be possible for anyone to know which RHM you talk about. The answers of all interviews will help to improve the RHM programme because thanks to the responses the strengths and weaknesses can be identified.	
rhm2n	rhm2: In the past 12 months, has a Rural Health Motivator visited you?<br>*Insert either month and year of the date (first two fields) or if the date is not known: If more than one year, enter years and months; if less than once year, enter months or weeks since last RHM visit. If the respondent does not know, enter 77 in weeks and 88 if the respondent refused to answer.*	1 Yes<br>2 No<br>77 Don't know<br>88 Refused
rhm4 *(required)*	rhm4: Which services did you receive from the Rural Health Motivator during the last visit?<br>*Do not read out the answer options. Select all that apply.*<br>*Question relevant when: ${rhm2n} = '1'*	1 Advice on how to eat healthy<br>2 Information on immunizations for children<br>3 Information and advice on feeding of babies<br>4 Information on pregnancy and childbirth<br>5 Checking if a pregnancy is going well<br>6 Advice or help with sanitation, such as toilets<br>7 Referral to a healthcare facility when I was ill<br>8 Care at home when I was ill<br>9 Observing me taking my medication<br>10 Information on family planning<br>11 Screening for tuberculosis<br>12 Advice on high blood pressure (hypertension)<br>13 Advice on high blood glucose (diabetes)<br>14 Advice on quitting tobacco use<br>15 Advice on reducing/quitting alcohol consumption<br>16 Advice on physical activity<br>17 Referral to a healthcare facility for a health check up<br>18 Screening for diabetes or hypertension<br>98 Other, specify
rhm4x *(required)*	rhm4x: Other services received by RHM<br>*Question relevant when: selected(${rhm4} , '98')*	
rhm8 *(required)*	rhm8: In the past 12 months, has your RHM advised you to go to a health care facility for a check-up or treatment?<br>*Question relevant when: ${rhm2n} = '1' and not(selected(${rhm4} , '7')) and not(selected(${rhm4} , '17'))*	1 Yes<br>2 No<br>77 Don't know<br>88 Refused
rhm10 *(required)*	rhm10: What was the reason for advising you to visit a health care facility?<br>*Do not read out the answer options. Select all that apply.*<br>*Question relevant when: ${rhm8} ='1'*	1 Overweight / high BMI<br>2 Symptoms of high blood sugar<br>3 Symptoms of high blood pressure<br>4 Symptoms of HIV<br>5 Symptoms of other disease<br>6 Injury<br>7 Treatment adherence / follow up (high BP)<br>8 Treatment adherence / follow up (diabetes)<br>9 Treatment adherence / follow up (HIV)

Field	Question	Answer
		10 Treatment adherence / follow up (other disease)<br>11 Routine check up<br>98 Other, specify
rhm10x *(required)*	rhm10x: Other reason for RHM advice to go to health care facility<br>*Question relevant when: selected(${rhm10} , '98')*	
rhm11 *(required)*	rhm11: Has your RHM ever advised you to go to a health care facility for a check-up or treatment because of raised blood sugar/diabetes/raised blood pressure/hypertension?<br>*Question relevant when: ${rhm2n} = '1' and not(selected(${rhm10} , '2')) and not(selected(${rhm10} , '3')) and not(selected(${rhm10} , '7')) and not(selected(${rhm10} , '8'))*	1 Yes<br>2 No<br>77 Don't know<br>88 Refused
Extended Individual Interview > Section 16: Rural Health Motivator > Last RHM referral<br>*Group relevant when: selected(${rhm10} , '2') or selected(${rhm10} , '3') or selected(${rhm10} , '7') or selected(${rhm10} , '8') or ${rhm11} ='1'*		
generated_note_name_321	rhm9: When was the last time s/he advised you to go to a health care facility for a check-up because of high blood sugar / diabetes or high blood pressure / hypertension?<br>*Insert either month and year of the date (first two fields) or one of the following: years, months, or weeks since last referral. If the respondent does not know, enter 77 in weeks and 88 if the respondent refused to answer.*	
rhm9m	Month:	
rhm9y	Year:<br>*Question relevant when: ${rhm9m} !=''*	
rhm9ys	Years since last RHM referral<br>*Question relevant when: ${rhm9m} ='' and ${rhm9y} =''*	
rhm9ms	Months since last RHM referral<br>*Question relevant when: ${rhm9m} ='' and ${rhm9y} ='' and ${rhm9ys} =''*	
rhm9ws *(required)*	Weeks since last RHM referral<br>*Question relevant when: ${rhm9m} ='' and ${rhm9y} ='' and ${rhm9ys} ='' and ${rhm9ms} =''*	
generated_note_name_328 *(required)*	The date you entered is in the future. Please revise.<br>*Question relevant when: ${rhm9y} = 2022 and ${rhm9m} > ${month}*	
generated_note_name_329 *(required)*	If you enter a year, you also need to enter a month.<br>*Question relevant when: ${rhm9y} != '' and ${rhm9m} =''*	
rhm12 *(required)*	rhm12: Did you go to the health-care facility for a check-up for raised blood sugar/diabetes/raised blood pressure/hypertension because the RHM suggested you to?<br>*apparently, if you click yes, then it takes you to rhm13x*<br>*Question relevant when: selected(${rhm10} , '2') or selected(${rhm10} , '3') or selected(${rhm10} , '7') or selected(${rhm10} , '8') or ${rhm11} ='1'*	1 Yes<br>2 No<br>77 Don't know<br>88 Refused
rhm13 *(required)*	rhm13: Why not?<br>*Do not read out the answer options. Select all that apply.*<br>*Question relevant when: ${rhm12} ='2'*	1 Need to work<br>2 Needed to take care of family members<br>3 Too far away from home<br>4 No money to pay for transport<br>5 No money to pay for health care services<br>6 Waiting times are too long<br>7 Low quality of services<br>8 Bad treatment by health care workers<br>9 Feeling uncomfortable during consultation<br>10 No need to go because I felt good<br>11 I forgot about it<br>13 I went to a traditional healer instead<br>14 There are no drugs available at the facility<br>98 Other, specify
rhm13x *(required)*	rhm13x: Other reason for not going to health care facility after RHM's advice<br>*Question relevant when: ${rhm13} ='98'*	
Extended Individual Interview > Section 17: Healthcare utilization and expenditures<br>*Group relevant when: ${ext_consent} ='1' and ${ps4n} =1*		
generated_note_name_337	READ:<br><br>In this section we are asking specifically about hospital visits where a patient was admitted, or required to stay overnight. In later sections we will ask about hospital visits that did not require an overnight stay, and visits to other health facilities like clinics.	
he1 *(required)*	he1: In the past 12 months, how many times were you admitted to the hospital?	
Extended Individual Interview > Section 17: Healthcare utilization and expenditures > Section 17.1: Last hospital admission<br>*Group relevant when: ${he1} >0*		
he4 *(required)*	READ:<br><br>I want to know more about why you needed an overnight stay in a hospital for your most recent visit.	

Field	Question	Answer
	he4: How many days was your last admission to the hospital?<br>*If the respondent does not know, enter 77 and 88 if the respondent refused to answer.*	
he7 *(required)*	he7: Which reason best describes why you were last hospitalised?	1 Communicable disease (infections, malaria, TB, HIV)<br>2 Maternal and perinatal conditions (pregnancy)<br>3 Nutritional deficiencies<br>4 Acute conditions (diarrhea, fever, flu, headaches, cough, other)<br>5 Injury (not occupation related)<br>6 Surgery<br>7 Sleep problems<br>8 Occupation/work related condition/injury<br>9 Chronic pain in your joints/arthritis (joints, back, neck)<br>10 Diabetes or related complications<br>11 Problems with your heart including unexplained pain in chest<br>13 Problems with your mouth, teeth or swallowing<br>14 Problems with your breathing<br>15 High blood pressure/hypertension<br>16 Stroke/sudden paralysis of one side of body<br>17 Generalized pain (stomach, muscle or other nonspecific pain)<br>18 Depression or anxiety<br>19 Cancer<br>20 Neurological disorder (epilepsy, multiple sclerosis, migraine)<br>21 Psychiatric disorder (schizophrenia)<br>22 COVID-19, Coronavirus<br>98 Other, specify
he7x *(required)*	he7x: Other reason for hospital admission<br>*Question relevant when: ${he7} ='98'*	
he8 *(required)*	he8: How much did you pay in fees or charges for your last hospital admission?<br>*in Lilangeni or Rand. If the respondent does not know, enter 777777 and 888888 if the respondent refused to answer.*	
he9 *(required)*	he9: How much did you pay for drugs/medications during your last hospital admission?<br>*in Lilangeni or Rand. If the respondent does not know, enter 777777 and 888888 if the respondent refused to answer.*	
he10 *(required)*	he10: What is the method of transport you used last time to get to the hospital? Was it by car, bike, motorbile/scooter, foot, public transport, or a privately hired taxi?	1 car<br>2 bicycle<br>3 motorbike/scooter<br>4 foot<br>5 public transport<br>6 privately hired taxi<br>98 Other, specify
he10x *(required)*	he10x: Other method of transport<br>*Question relevant when: ${he10} ='98'*	
Extended Individual Interview > Section 17: Healthcare utilization and expenditures > Section 17.1: Last hospital admission > Travel time of last hospital admission		
generated_note_name_349	he12: How long does the journey by [he10_name] take to go from your home to the hospital?<br>*You have to enter hours and minutes. If the respondent does not know, enter 77 in hours and minutes and 88 if the respondent refused to answer.*	
he12h *(required)*	Hours:<br>*If less than 1 hour, enter 0.*	
he12m *(required)*	Minutes:<br>*If more than 60 minutes, convert to hours and minutes.*	
he14 *(required)*	he14: On the last hospital visit how many persons over the age of 6 accompanied you?<br>*If the respondent does not know, enter 77 and 88 if the respondent refused to answer.*	
he17 *(required)*	he17: How much was spent on **accommodation** that the persons (children and adults) accompanying you needed because of the last admission to the hospital?<br>*in Lilageni/Rand.*<br>*Question relevant when: ${he14} > 0 and ${he14} < 77*	1 0<br>2 1 - 99<br>3 100 - 499

Field	Question	Answer
		4 500 - 1199<br>5 1200 and more<br>77 Don't know<br>88 Refused
he18new *(required)*	he18: How much was spent on **food** that the persons (children and adults) accompanying you needed because of the last admission to the hospital?<br>*in Lilageni/Rand.*<br>*Question relevant when: ${he14} > 0 and ${he14} < 77*	1 0<br>2 1-24<br>3 25-49<br>4 50-99<br>5 100-149<br>6 150-249<br>7 250 and above<br>77 Don't know<br>88 Refused
he19 *(required)*	he19: How much was spent on **transport** from your home to the hospital for you and the persons (children and adults) accompanying for the last hospital admission?<br>*in Lilageni/Rand.*	1 0<br>2 1 - 49<br>3 50 - 99<br>4 100 -149<br>5 150 - 199<br>6 200 - 249<br>7 250 - 299<br>8 300 and more<br>77 Don't know<br>88 Refused
hecov *(required)*	hecov: Have you ever been hospitalized because of Covid-19?<br>*Question relevant when: not(selected(${he7} ,'22'))*	1 Yes<br>2 No<br>77 Don't know<br>88 Refused
Extended Individual Interview > Section 17: Healthcare utilization and expenditures > Section 17.2: Outpatient hospital visits		
generated_note_name_361	READ:<br><br>In this section we are asking specifically about hospital visits which did NOT require an overnight hospital visit. Such visits, called outpatient visits, are when the person requiring treatment is not admitted to the hospital. Such hospital visits can be for one single day or for more than one day where the patient is asked to come back the next day. In the next section we will ask about visits to other health facilities such as clinics.	
he20 *(required)*	he20: How many times did you visit a hospital in the last 12 months where you were not admitted to stay the night?	
Extended Individual Interview > Section 17: Healthcare utilization and expenditures > Section 17.2: Outpatient hospital visits > lasthospitalvisit<br>*Group relevant when: ${he20} >0*		
Extended Individual Interview > Section 17: Healthcare utilization and expenditures > Section 17.2: Outpatient hospital visits > lasthospitalvisit > Waiting time during last hospital visit		
generated_note_name_365	READ:<br><br>I want to know more about your most recent hospital visit for outpatient care.<br><br>he23: During your last visit, how much time did you spend waiting at the hospital for the appointment?<br>*You have to enter hours and minutes. If the respondent does not know, enter 77 in hours and minutes and 88 if the respondent refused to answer.*	
he23h *(required)*	Hours:<br>*If less than 1 hour, enter 0.*	
he23m *(required)*	Minutes<br>*If more than 60 minutes, convert to hours and minutes.*	
Extended Individual Interview > Section 17: Healthcare utilization and expenditures > Section 17.2: Outpatient hospital visits > lasthospitalvisit > Time in consultation during last hospital visit		
generated_note_name_370	he24 During your last visit, how much time did you spend in the consultation?<br>*You have to enter hours and minutes. If the respondent does not know, enter 77 in hours and minutes and 88 if the respondent refused to answer.*	
he24h *(required)*	Hours:<br>*If less than 1 hour, enter 0.*	
he24m *(required)*	Minutes<br>*If more than 60 minutes, convert to hours and minutes.*	
he27 *(required)*	he27: Which reason best describes why you needed this visit?	1 Communicable disease (infections, malaria, TB, HIV)<br>2 Maternal and perinatal conditions (pregnancy)<br>3 Nutritional deficiencies<br>4 Acute conditions (diarrhea, fever, flu, headaches, cough, other)<br>5 Injury (not occupation related)<br>6 Surgery<br>7 Sleep problems<br>8 Occupation/work related condition/injury

Field	Question	Answer
		9 Chronic pain in your joints/arthritis (joints, back, neck)<br>10 Diabetes or related complications<br>11 Problems with your heart including unexplained pain in chest<br>13 Problems with your mouth, teeth or swallowing<br>14 Problems with your breathing<br>15 High blood pressure/hypertension<br>16 Stroke/sudden paralysis of one side of body<br>17 Generalized pain (stomach, muscle or other nonspecific pain)<br>18 Depression or anxiety<br>19 Cancer<br>20 Neurological disorder (epilepsy, multiple sclerosis, migraine)<br>21 Psychiatric disorder (schizophrenia)<br>22 COVID-19, Coronavirus<br>98 Other, specify
he27x *(required)*	he27x: Other reason for last hospital visit<br>*Question relevant when: ${he27} ='98'*	
he28 *(required)*	he28: Which services were provided to you?	1 Cervical cancer screening (via VIA)<br>2 Breast cancer screening by palpitation<br>3 Prostate cancer screening by palpitation<br>4 Diabetes screening<br>5 Hypertension screening<br>6 Nutritional assessment (BMI, MUAC, promotion of healthy diet)<br>7 HIV testing<br>8 TB testing<br>9 Asking for STI symptoms<br>10 Testing for STIs<br>11 Family planning need assessment<br>12 Screening for gender-based violence<br>13 Mental health assessment<br>14 Drug provision<br>98 Other, specify
he28x *(required)*	he28x: Other services provided<br>*Question relevant when: selected(${he28} , '98')*	
he29 *(required)*	he29: How much did you pay in fees or charges for your last hospital visit?<br>*in Lilangeni or Rand. If the respondent does not know, enter 777777 and 888888 if the respondent refused to answer.*	
he30 *(required)*	he30: How much did you pay for drugs/medications during your last hospital visit?<br>*in Lilangeni or Rand. If the respondent does not know, enter 777777 and 888888 if the respondent refused to answer.*	
he31 *(required)*	he31: What is the method of transport you used last time to get to the hospital? Was it by car, bike, motorbile/scooter, foot, public transport, or a privately hired taxi?	1 car<br>2 bicycle<br>3 motorbike/scooter<br>4 foot<br>5 public transport<br>6 privately hired taxi<br>98 Other, specify
he31x *(required)*	he31x: Other method of transport<br>*Question relevant when: ${he31} ='98'*	
Extended Individual Interview > Section 17: Healthcare utilization and expenditures > Section 17.2: Outpatient hospital visits > lasthospitalvisit > Travel time of last hospital visit		
generated_note_name_384	he33: How long does the journey by [he31_name] take to go from your home to the hospital?<br>*You have to enter hours and minutes. If the respondent does not know, enter 77 in hours and minutes and 88 if the respondent refused to answer.*	

Field	Question	Answer
he33h *(required)*	Hours:<br>*If less than 1 hour, enter 0.*	
he33m *(required)*	Minutes<br>*If more than 60 minutes, convert to hours and minutes.*	
he34 *(required)*	he34: On the last hospital visit how many persons aged 6 and above accompanied you?<br>*If the respondent does not know, enter 77 and 88 if the respondent refused to answer.*	
he38new *(required)*	he38: How much was spent on **food** that you and the persons (children and adults) accompanying you needed because of the last visit to the hospital?<br>*in Lilageni/Rand.*	1 0<br>2 1-24<br>3 25-49<br>4 50-99<br>5 100-149<br>6 150-249<br>7 250 and above<br>77 Don't know<br>88 Refused
he39 *(required)*	he39: How much was spent on **transport** that you and the persons (children and adults) accompanying you needed because of the last hospital visit?<br>*in Lilageni/Rand.*	1 0<br>2 1 - 49<br>3 50 - 99<br>4 100 -149<br>5 150 - 199<br>6 200 - 249<br>7 250 - 299<br>8 300 and more<br>77 Don't know<br>88 Refused
Extended Individual Interview > Section 17: Healthcare utilization and expenditures > Section 17.3: Clinic visits		
generated_note_name_395	READ:<br><br>In this section we are asking specifically about visits to community clinics or healthcare facilities at the community level other than those to a hospital. A hospital is typically a larger facility offering more sophisticated procedures for surgeries and a wide range of other medical services. Hospitals tend to be located in urban areas and bigger towns and cities. Clinics and smaller healthcare facilities are smaller establishments and generally offer a limited selection of services and treatment consultation. More complicated procedures at clinics are referred to hospitals. Clinics are primary healthcare facilities while hospitals are tertiary facilities. Clinics are also more likely to be found in villages and rural areas and typically provides non-urgent care that's routine or preventative.	
he47 *(required)*	he47: How many times did you visit a clinic in the last 12 months?	
he53 *(required)*	he53: What is the name of the clinic you visited last time?<br>*Question relevant when: ${he47} >0*	1 Mondi Forest Clinic<br>2 Bulembu Clinic (Havelock)<br>3 Swazico Med (Clinic and Mobile Clinic)<br>4 Maguga Clinic<br>5 Mshingishingini Nazarene Clinic<br>6 Medisun Clinic<br>7 Mangedla Clinic<br>8 Mbabane Public Health Unit<br>9 Ekuphileni Clinic<br>10 S&P Health Care Centre<br>11 Jikani Lambu Medical Center<br>12 Satellite Clinic<br>13 Hhukwini Clinic<br>14 Millsite Clinic<br>15 Mhlambanyatsi Clinic 2<br>16 Ezulwini Clinic (Pharmacy)<br>17 Salvation Army Clinic (Mbabane)<br>18 Siphocosini Clinic<br>19 Ngwenya Wellness Centre<br>20 Nkaba Clinic<br>21 Children's Clinic (Dr Rukundo)<br>22 Ntfonjeni Clinic<br>23 Baphiwe Healthcare and wellness Clinic

Field	Question	Answer		
			24	Regina Mundi Clinic / Mondi clinic
			25	Psychiatric Clinic
			26	Piggs' Peak Public Health Unit
			27	Malandzela Nazarene Clinic
			28	Ekuphileni Medical Clinic - Dr S.P.N Shongwe
			29	Ngonini Estate Clinic
			30	Vusweni Clinic
			31	Ngowane Clinic
			32	Sigangeni Clinic
			33	UNISWA Mbabane Campus
			34	Ekufikeni clinic
			35	Giving Life Clinic
			36	Pigg's Peak Nazarene Clinic
			37	National Baptist Mission Clinic
			38	Mdzimba UEDF Clinic
			39	Hhelehhele 11 Clinic
			40	Herefords Community Clinic
			41	Mbabane Government Hospital
			42	The Clinic Group
			43	Motshane Community Clinic
			44	Diabeties Clinic
			45	Mangweni Clinic
			46	Mbabane Correctional Services clinic
			47	Nkoyoyo UEDF Clinic
			48	Ndzingeni Nazarene Clinic
			49	SCU Health Centre
			50	Family Life Clinic (Mbabane)
			51	Nyonyane Clinic
			52	Dvokolwako Health Centre
			53	Family Care Clinic
			54	Childrens Clinic
			55	Emkhuzweni Health Center
			56	Horo Clinic
			57	Bhalekane Nazarene Clinic
			58	University of Limkokwing Clinic
			59	Nkoyoyo Clinic
			60	Dr Eboyens & Partners Clinic
			61	St. Mary's Clinic
			62	Ensingweni Clinic (formerly outreach)
			63	Manzana Clinic (Special Health Care Unit)
			64	Maphalaleni Clinic
			65	Ndvwabangeni Nazarene Clinic
			66	Occupational Theraphy Clinic Mbabane Gov Hospital
			67	Carers Corner Clinic
			68	Mbabane City Council Clinic

Field	Question	Answer		
			69	Amicall Ngwenya
			70	Nsingizini UEDF Clinic
			71	The Clinic (Mbabane)
			72	Mbuluzi Salvation Army Clinic
			73	Siyanaka Medical Centre
			74	Mbasheni Clinic
			75	Piggs Peak Correctional Services Clinic
			76	Lobamba Clinic
			77	Ntintiza Clinic
			78	Dr Stephens Clinic
			79	Clicks clinics (The Gables outlet)
			80	Pigg's Peak Government Hospital
			81	SOS Children's Village Clinic (Mbabane)
			82	Nkabave Clinic
			83	Clicks clinics (Swazi Plaza Outlet)
			84	Correctional Clinic (Bhalekane)
			85	Bulandzeni Clinic
			86	Mahwalala Red Cross Clinic
			87	Ngwenya Port Health Clinic
			88	Sitsatsaweni Nazerene Clinic
			89	Cabrini Ministries Health Care
			90	Mhlume Medical Services
			91	Mambane Clinic
			92	Lubombo Referral
			93	Siphofaneni Clinic
			94	Big Bend Prison Clinic
			95	C.M.C.D Ravenna Clinic
			96	St. Phillip's Clinic
			97	Ubombo Sugar Hospital
			98	Tikhuba Clinic
			99	Lubuli Clinic
			100	Khuphuka Clinic
			101	Manyeveni Nazarene Clinic
			102	Sigcaweni Nazarene Clinic
			103	Siteki Public Health Unit
			104	Mpolonjeni Clinic
			105	Ngwavuma USDF
			106	Flame Clinic (CLOSED)
			107	Matata Clinic
			108	Ikwezi Joy Clinic
			109	C.G.I Clinic
			110	Nkalashane Community Clinic
			111	Mkhaya Clinic-Siteki
			112	Gilgal Clinic
			113	Gucuka Clinic (formerly outreach site)
			114	Tabankulu Estates Clinic
			115	Ngwavuma UEDF Clinic
			116	Mbalenhle Clinic
			117	Tshaneni Clinic
			118	Hlane Clinic
			119	Ebenezer Clinic
			120	Lomahasha Clinic

Field	Question	Answer

	121	Good Shepherd Public Health Center
	122	KM III Clinic
	123	New Thulwane Clinic
	124	Mpaka Railway Clinic
	125	Tambuti Estate Clinic
	126	Vuvulane Clinic
	127	SOS Clinic (Ekutfokomeni clinic)
	128	Maloma Colliery Clinic
	129	Sitobela Rural Health Center
	130	UTECH Clinic
	131	Mill Clinic
	132	Sinceni Clinic
	133	Shewula Nazarene Clinic
	134	Anchor Clinic
	135	Tsambokulu Clinic
	136	Dr Martins Clinic
	137	Kudvumisa Foundation
	138	Ndzevane Clinic
	139	Nkonjwa Clinic
	140	Malindza Refugee Camp Clinic
	141	Siphofaneni Private Clinic
	142	Siteki Nazarene Clinic
	143	Mlindazwe UEDF Clinic
	144	Good Shepherd Hospital
	145	Bholi Clinic
	146	Ngomane Clinic
	147	Matsetsa Private Clinic
	148	Sikhuphe Airport Clinic
	149	Sibovu Clinic (Mahlangatsha)
	150	Sitsembinkosi Clinic
	151	Mkhulamini Clinic
	152	Teba Clinic-Manzini
	153	Women And Men Health Care Clinic
	154	Clinic 2000 (Dr Mbelu)
	155	Ngonini (OSSU) Clinic
	156	Bhekinkosi Nazarene Clinic
	157	Mahlangatsha Inkhundla
	158	Cana Mission Clinic
	159	Ka-Zondwako Clinic
	160	Family Life Association Clinic (Manzini)
	161	Criminal Lunatic Clinic
	162	Manzini Private Clinic (Imphilo)
	163	Clicks Clinic (Manzini Bhunu Mall)
	164	Kabhudla Clinic
	165	Mawelawela Women Correctional Services Clinic
	166	Leo Garments Clinic
	167	YKK Clinic
	168	National Textile Clinic
	169	Sicalo Health Clinic
	170	Magubheleni Clinic
	171	Nsingizini USDF
	172	RSP Clinic
	173	Mankayane Hospital
	174	Mankayane Public Health Unit
	175	Ancher Clinic

Field	Question	Answer		
			176	Raleigh Fitkin Memorial Hospital
			177	Luyengo Clinic
			178	Mona Healthlife Clinic
			179	Wellness Center Clinic
			180	Lomgelatshane Clinic (Sidvokodvo)
			181	Kabulin Copporels PTY (LTD) Clinic
			182	Etetsembisweni Clinic
			183	Musi Clinic
			184	Swazican Clinic
			185	Mangcongco Clinic
			186	Mobile Clinic(PPP)Matsapha Town Council
			187	SWAPOL Clinic
			188	Ncabaneni Clinic
			189	Hillside Clinic
			190	Mkhaya Clinic
			191	Phocweni Clinic (UEDF)
			192	Gcina UEDF Clinic
			193	Malkerns Family Life Association
			194	LTD Clinic
			195	Women and Children Hospital
			196	Bulunga Nazarene Clinic
			197	New Village Nazarene Clinic
			198	Mbuluzi UEDF Clinic
			199	Mankayane Correctional Services Clinic
			200	Lemlandvo Clinic
			201	St. Juliana's Clinic
			202	Heart For Africa-Elrofi Clinic
			203	Mliba Nazarene Clinic
			204	The Luke Commission
			205	Phiwinhlanhla Clinic
			206	Philani Clinic (Manzini)
			207	Union Washing (LTD) Clinic
			208	Litsemba Letfu Men's Clinic
			209	Proton Investment Clinic
			210	Lushikishini Clinic
			211	Emoyeni Clinic
			212	Phumelele Clinic
			213	Sigombeni Red Cross Clinic
			214	Kwaluseni University Clinic
			215	Mother Care Clinic
			216	Sibonginkosi Clinic
			217	Malkerns Juvenile Industrial School Clinic
			218	Sappi Health Centre
			219	Nhlambeni Clinic
			220	Dwalile Clinic
			221	Garrison UEDF Clinic
			222	Giant Clothing Clinic
			223	Philani Clinic (Matsapha)
			224	Ekudzeni Thole Clinic
			225	Mdzimba Clinic USDF
			226	Gcina Bethany Clinic
			227	Texray Clinic
			228	Bethany Clinic

Field	Question	Answer		
			229	Ngculwini Nazarene Clinic
			230	Bhudla Clinic
			231	Malkerns Clinic USDF
			232	Simply Aid Medical Services
			233	Nonhlanhla Clinic
			234	Lulama Health Clinic
			235	Manzini Government Hospital
			236	NAMPAK Clinic
			237	Mathangeni Church of Christ Clinic
			238	Lamvelase Clinic (Zombodze)
			239	Siphiwo Clinic (formerly Sicelwini)
			240	Psychiatric Hospital (National)
			241	Sigcineni Clinic
			242	RSP VCT
			243	King Sobhuza II Health Unit
			244	Ekuthuleni Clinic
			245	Manzini Health Care (Dr Mathunjwa)
			246	Manzini Town Council
			247	St. Florence Clinic
			248	Kwaluseni Clinic
			249	Shamar Family (shammah) center Clinic
			250	Luve Clinic
			251	Nkhabave clinic
			252	Matsapha Unitrans Swaziland Wellness Clinic
			253	Siphosemphilo Clinic (Diabetes)
			254	Ziong Tian Clinic
			255	Bhunya Mill Clinic
			256	Mpuluzi Clinic
			257	Sikhuphe Airport Clinic
			258	Engculwini Clinic
			259	Bhahwini Clinic
			260	Temantungwa Clinic
			261	Women & Men Healthcare Clinic
			262	Lunyengo Student Clinic
			263	Mkhiwa Clinic - Manzini
			264	Mahlanya Clinic Dr L Shongwe
			265	Gebeni Clinic
			266	Dr S Hynd - Manzini Medical Center
			267	Sidvokodvo Railway Clinic
			268	Mliba Nazarene Clinic
			269	St. Theresa's Clinic
			270	Criminal Lunatic Assylum Clinic
			271	KaGogo Mamba Clinic
			272	Mbikwakhe Clinic
			273	Mafutseni Nazerene Clinic
			274	Maloyi Clinic
			275	Homeopathy & Physio Clinic
			276	Manzana Clinic (Special Health Care Unit)
			277	TASC Manzini
			278	Correctional College Staff Clinic

Field	Question	Answer
		279 Ngonini Royal Clinic (Special Health Care Unit)<br>280 Mhlambanyatsi Clinic<br>281 Hlathikhulu Police Wellness Clinic<br>282 Baylor Clinic - RFM<br>283 Matsanjeni Public Health Unit<br>284 Lavumisa Clinic<br>285 Nhletsheni Clinic<br>286 Casualty Department Hlatikulu Hospital<br>287 Dwaleni Clinic<br>288 Mbangweni UEDF Clinic<br>289 New Haven Clinic<br>290 Lavumisa Wellness Clinic<br>291 Zheng Yong<br>292 Mhlosheni Clinic<br>293 Matsanjeni Health Center<br>294 Gege Clinic<br>295 Hlatikhulu Hospital<br>296 Nhlangano Public Health Unit<br>297 JCI (Mphelandzaba) Clinic<br>298 Our Lady of Sorrows Clinic<br>299 Mahlandle Clinic<br>300 FTM Clinic<br>301 Jericho Clinic<br>302 Mkhitsini Clinic<br>303 Hluti Clinic<br>304 SOS Clinic (Nhlangano)<br>305 Zombodze Clinic (Shiselweni)<br>306 Phunga Clinic<br>307 Nsalitje Clinic<br>308 Kaphunga Nazarene Clinic<br>309 KaMfishane (KaNdlovu) Clinic<br>310 Nhlangano Health Center<br>311 Nkwene Clinic<br>312 Moti Clinic<br>313 Silele Red Cross Clinic<br>314 Mashobeni Clinic<br>315 Nhlangano Correctional Clinic<br>316 Philani Clinic (Nhlangano)<br>317 Nhlangano H.C Wellness Clinic<br>318 Magubheleni Clinic<br>319 Mgazini Clinic<br>320 Hlatikhulu Public Health Unit<br>321 Bethany Clinic<br>322 Hlathikhulu Wellness Clinic<br>323 Ntshanini Clinic<br>324 Tfokotani Clinic<br>325 Nhlangunjani Clinic<br>326 Luyengo Students Clinic<br>327 Lubombo Police Regional Clinic<br>328 Mbabane Male Wellness Clinic<br>329 Mananga Clinic<br>330 Hhohho Regional Police Clinic

Field	Question	Answer
he53x *(required)*	he53x: Other name of clinic<br>*Question relevant when: ${he53} ='99998'*	331 Ezindwendweni Clinic<br>333 Ezulwini Private Hospital
he48 *(required)*	he48: Did you always visit the same clinic?<br>*Question relevant when: ${he47} >1*	334 South Africa<br>99998 Other, specify<br>1 Yes<br>2 No<br>77 Don't know<br>88 Refused
Extended Individual Interview > Section 17: Healthcare utilization and expenditures > Section 17.3: Clinic visits > lastclinicvisit<br>*Group relevant when: ${he47} >0*		
Extended Individual Interview > Section 17: Healthcare utilization and expenditures > Section 17.3: Clinic visits > lastclinicvisit > Date of last clinic visit		
generated_note_name_403	READ:<br><br>I want to know more about your most recent visit to the clinic.<br><br>he51: When did you last visit a clinic?<br>*Insert either month and year of the date (first two fields) or one of the following: years, months, or weeks since last clinic visit. If the respondent does not know, enter 77 in weeks and 88 if the respondent refused to answer.*	
he51m	Month:	
he51y	Year:<br>*Question relevant when: ${he51m} !=''*	
he51ys	Year since last clinic visit<br>*Question relevant when: ${he51m} ='' and ${he51y} =''*	
he51ms	Months since last clinic visit<br>*Question relevant when: ${he51m} ='' and ${he51y} ='' and ${he51ys} =''*	
he51ws *(required)*	Weeks since last clinic visit<br>*Question relevant when: ${he51m} ='' and ${he51y} ='' and ${he51ys} ='' and ${he51ms} =''*	
generated_note_name_410 *(required)*	The date you entered is in the future. Please revise.<br>*Question relevant when: ${he51y} = 2022 and ${he51m} > ${month}*	
generated_note_name_411 *(required)*	If you enter a year, you also need to enter a month.<br>*Question relevant when: ${he51y} != '' and ${he51m} =''*	
generated_note_name_413	The date you entered is more than 12 months ago. Please revise.<br>*Question relevant when: ${clinicvisitdate} =0 and ${he47} >0*	
Extended Individual Interview > Section 17: Healthcare utilization and expenditures > Section 17.3: Clinic visits > lastclinicvisit > Waiting time during last clinic visit		
generated_note_name_418	he49: During your last visit, how much time did you spend waiting at the clinic for the appointment?<br>*You have to enter hours and minutes. If the respondent does not know, enter 77 in hours and minutes and 88 if the respondent refused to answer.*	
he49h *(required)*	Hours:<br>*If less than 1 hour, enter 0.*	
he49m *(required)*	Minutes<br>*If more than 60 minutes, convert to hours and minutes.*	
Extended Individual Interview > Section 17: Healthcare utilization and expenditures > Section 17.3: Clinic visits > lastclinicvisit > Time in consultation during last clinic visit		
generated_note_name_423	he50: During your last visit, how much time did you spend in the consultation?<br>*You have to enter hours and minutes. If the respondent does not know, enter 77 in hours and minutes and 88 if the respondent refused to answer.*	
he50h *(required)*	Hours:<br>*If less than 1 hour, enter 0.*	
he50m *(required)*	Minutes<br>*If more than 60 minutes, convert to hours and minutes.*	
he54 *(required)*	he54: Which reason best describes why you needed this visit?	1 Communicable disease (infections, malaria, TB, HIV)<br>2 Maternal and perinatal conditions (pregnancy)<br>3 Nutritional deficiencies<br>4 Acute conditions (diarrhea, fever, flu, headaches, cough, other)<br>5 Injury (not occupation related)<br>6 Surgery<br>7 Sleep problems<br>8 Occupation/work related condition/injury<br>9 Chronic pain in your joints/arthritis (joints, back, neck)<br>10 Diabetes or related complications<br>11 Problems with your heart including unexplained pain in chest<br>13 Problems with your mouth, teeth or swallowing<br>14 Problems with your breathing

Field	Question	Answer
		15 High blood pressure/hypertension<br>16 Stroke/sudden paralysis of one side of body<br>17 Generalized pain (stomach, muscle or other nonspecific pain)<br>18 Depression or anxiety<br>19 Cancer<br>20 Neurological disorder (epilepsy, multiple sclerosis, migraine)<br>21 Psychiatric disorder (schizophrenia)<br>22 COVID-19, Coronavirus<br>98 Other, specify
he54x *(required)*	he54x: Other reason for last clinic visit<br>*Question relevant when: ${he54} ='98'*	
he55 *(required)*	he55: Which services were provided to you?	1 Cervical cancer screening (via VIA)<br>2 Breast cancer screening by palpitation<br>3 Prostate cancer screening by palpitation<br>4 Diabetes screening<br>5 Hypertension screening<br>6 Nutritional assessment (BMI, MUAC, promotion of healthy diet)<br>7 HIV testing<br>8 TB testing<br>9 Asking for STI symptoms<br>10 Testing for STIs<br>11 Family planning need assessment<br>12 Screening for gender-based violence<br>13 Mental health assessment<br>14 Drug provision<br>98 Other, specify
he55x *(required)*	he55x: Other services provided<br>*Question relevant when: selected(${he55} , '98')*	
he56 *(required)*	he56: How much did you pay in fees or charges for your last clinic visit?<br>*in Lilangeni or Rand. If the respondent does not know, enter 777777 and 888888 if the respondent refused to answer.*	
he57 *(required)*	he57: How much did you pay for drugs/medications for your last clinic visit?<br>*in Lilangeni or Rand. If the respondent does not know, enter 777777 and 888888 if the respondent refused to answer.*	
he58 *(required)*	he58: What was the method of transport you used last time to get to the clinic appointment? Was it by car, bicycle, motorbile/scooter, foot, public transport, or a privately hired taxi?	1 car<br>2 bicycle<br>3 motorbike/scooter<br>4 foot<br>5 public transport<br>6 privately hired taxi<br>98 Other, specify
he58x *(required)*	he58x: Other method of transport<br>*Question relevant when: ${he58} ='98'*	
Extended Individual Interview > Section 17: Healthcare utilization and expenditures > Section 17.3: Clinic visits > lastclinicvisit > Travel time of last clinic visit		
generated_note_name_437	he60: How long does the journey by [he58_name] take to go from your home to the clinic?<br>*You have to enter hours and minutes. If the respondent does not know, enter 77 in hours and minutes and 88 if the respondent refused to answer.*	
he60h *(required)*	Hours:<br>*If less than 1 hour, enter 0.*	
he60m *(required)*	Minutes<br>*If more than 60 minutes, convert to hours and minutes.*	
he61 *(required)*	he61: On your last clinic visit how many persons over the age of 6 accompanied you?<br>*If the respondent does not know, enter 77 and 88 if the respondent refused to answer.*	
he65new *(required)*	he65: How much was spent on **food** that you and the persons (children and adults) accompanying you needed because of the last visit to the clinic?<br>*in Lilageni/Rand.*	1 0<br>2 1-24<br>3 25-49<br>4 50-99<br>5 100-149<br>6 150-249

Field	Question	Answer
		7 250 and above<br>77 Don't know<br>88 Refused
he66 *(required)*	he66: How much was spent on **transport** that you and the persons (children and adults) accompanying you needed because of the last hospital clinic?<br>*in Lilageni/Rand.*	1 0<br>2 1 - 49<br>3 50 - 99<br>4 100 -149<br>5 150 - 199<br>6 200 - 249<br>7 250 - 299<br>8 300 and more<br>77 Don't know<br>88 Refused
Extended Individual Interview > Section 17: Healthcare utilization and expenditures > Section 17.4: Visit of traditional healer		
he74 *(required)*	he74: Did you seek advice or treatment with a traditional healer for any health condition?	1 Yes<br>2 No<br>77 Don't know<br>88 Refused
he75 *(required)*	he75: Which reasons best describe why you visited a traditional healer?<br>*Question relevant when: ${he74} ='1'*	1 Communicable disease (infections, malaria, TB, HIV)<br>2 Maternal and perinatal conditions (pregnancy)<br>3 Nutritional deficiencies<br>4 Acute conditions (diarrhea, fever, flu, headaches, cough, other)<br>5 Injury (not occupation related)<br>6 Surgery<br>7 Sleep problems<br>8 Occupation/work related condition/injury<br>9 Chronic pain in your joints/arthritis (joints, back, neck)<br>10 Diabetes or related complications<br>11 Problems with your heart including unexplained pain in chest<br>13 Problems with your mouth, teeth or swallowing<br>14 Problems with your breathing<br>15 High blood pressure/hypertension<br>16 Stroke/sudden paralysis of one side of body<br>17 Generalized pain (stomach, muscle or other nonspecific pain)<br>18 Depression or anxiety<br>19 Cancer<br>20 Neurological disorder (epilepsy, multiple sclerosis, migraine)<br>21 Psychiatric disorder (schizophrenia)<br>22 COVID-19, Coronavirus<br>98 Other, specify
he75x *(required)*	he75x: Other reason for visiting traditional healer<br>*Question relevant when: ${he75} ='98'*	
Extended Individual Interview > Section 17: Healthcare utilization and expenditures > Other healthcare expenditure		
he76 *(required)*	he76: In the last six months, did you incur any out-of-pocket (OOP) expenditures for healthcare? If yes, how did you fund them?	1 No OOP healthcare expenses incurred<br>2 Savings<br>3 Sold personal/household items<br>4 Borrowed from relatives<br>5 Borrowed from other sources

Field	Question	Answer
		77 Don't know<br>88 Refused
he77 *(required)*	he77: How much did you spend on other health care in the past SIX months, such as traditional medicines, medicines from a convenience store, special food, etc.?<br>*in Lilangeni or Rand. If the respondent does not know, enter 777777 and 888888 if the respondent refused to answer.*	
he80 *(required)*	he80: Do you have a health insurance or medical aid?	1 Yes<br>2 No<br>77 Don't know<br>88 Refused
Extended Individual Interview > Section 18: Knowledge on diabetes and hypertension<br>*Group relevant when: ${ext_consent} ='1' and ${ps4n} =1*		
generated_note_name_461	READ:<br><br>Now I will read out some statements about hypertension. Please tell me whether you think they are true or false. If you are not sure or don't know, state the answer that you think could be correct	
ta1 *(required)*	ta1: A person always knows when they have high blood pressure / hypertension or high blood sugar / diabetes.	1 True<br>2 False
ta2 *(required)*	ta2: If you have a family history of high blood pressure / hypertension or high blood sugar / diabetes, you are at risk for developing high blood pressure / hypertension or high blood sugar / diabetes.	1 True<br>2 False
ta3 *(required)*	ta3: Young people have the same probability of having high blood pressure / hypertension or high blood sugar / diabetes as old people.	1 True<br>2 False
ta4 *(required)*	ta4: Having high blood pressure / hypertension or high blood sugar / diabetes puts you at higher risk of developing heart disease.	1 True<br>2 False
ta5 *(required)*	ta5: Eating salty food helps a person to lower their blood pressure.	1 True<br>2 False
ta6 *(required)*	ta6: Regular physical activity will lower a person's chance of getting high blood pressure / hypertension or high blood sugar / diabetes.	1 True<br>2 False
ta7 *(required)*	ta7: A person who has high blood pressure / hypertension or high blood sugar / diabetes can reduce their risk of developing heart disease if they keep their blood pressure levels under control.	1 True<br>2 False
ta8 *(required)*	ta8: If a person forgets to take their high blood pressure / hypertension or high blood sugar / diabetes medication, they should take twice the dosage the next time.	1 True<br>2 False
ta9 *(required)*	ta9: A person who has high blood pressure / hypertension or high blood sugar / diabetes can reduce their risk of developing heart disease if they keep their weight under control.	1 True<br>2 False
Extended Individual Interview > Section 18: Knowledge on diabetes and hypertension > lse<br>*Group relevant when: ${hd2} = '1'*		
generated_note_name_474	READ:<br><br>ta18: If you feel the beginnings of a low blood sugar episode reaction, you should immediately…	
ta18	Options	1 True<br>2 False
ta18a *(required)*	take some insulin or your daily diabetes medication	1 True<br>2 False
ta18b *(required)*	lie down and rest	1 True<br>2 False
ta18c *(required)*	eat or drink something sweet.	1 True<br>2 False
Extended Individual Interview > Section 19: Behavioural measurements<br>*Group relevant when: ${ext_consent} ='1' and ${ps4n} =1*		
bm1 *(required)*	READ:<br><br>Now I am going to ask you some questions about tobacco use, physical activity and fruit and vegetable consumption.<br><br>bm1: Do you currently smoke any tobacco products, such as cigarettes, cigars or pipes? And if yes, do you smoke daily or occasionally?	1 Yes, daily<br>2 Yes, occasionally<br>3 No<br>88 Refused
bm3 *(required)*	bm3: In the past, did you ever smoke any tobacco products? And if yes, did you smoke daily or occasionally?<br>*Question relevant when: ${bm1} ='3'*	1 Yes, daily<br>2 Yes, occasionally<br>3 No<br>88 Refused
bm4n *(required)*	bm4: Have you quit smoking in the past 12 months?<br>*Question relevant when: ${bm3} ='1' or ${bm3} ='2'*	1 Yes<br>2 No<br>77 Don't know<br>88 Refused
generated_note_name_487	READ:<br>We are interested in finding out about the kinds of physical activities that people do as part of their everyday lives. The questions will ask you about the time you spent being physically active in the last 7 days. Please answer each question even if you do not consider yourself to be an active person. Please think about the activities you do at	

Field	Question	Answer
	work, as part of your house and yard work, to get from place to place, and in your spare time for recreation, exercise or sport.<br><br>Think about all the vigorous activities that you did in the last 7 days. Vigorous physical activities refer to activities that take hard physical effort and make you breathe much harder than normal. Think only about those physical activities that you did for at least 10 minutes at a time	
bm6 *(required)*	bm6: During the last 7 days, on how many days did you do vigorous physical activities like heavy lifting, digging, aerobics, or fast bicycling?<br>*Includes activity at work, travel to and from places, and recreation. If the respondent does not know, enter 77.*	
Extended Individual Interview > Section 19: Behavioural measurements > Vigorous physical activity<br>*Group relevant when: ${bm6} >0 and ${bm6} < 77*		
generated_note_name_490	bm7: How much time did you usually spend doing vigorous physical activities on one of those days?<br>*You have to enter hours and minutes. If the respondent does not know, enter 77 in hours and minutes.*	
bm7h *(required)*	hours<br>*If less than 1 hour, enter 0.*	
bm7m *(required)*	minutes<br>*If more than 60 minutes, convert to hours and minutes.*	
generated_note_name_495 *(required)*	You have to enter more than 0 hours and 0 minutes of vigorous activity<br>*Question relevant when: ${vigzero} =0 and ${bm6} >0 and ${bm6} < 77*	
generated_note_name_496	READ:<br><br>Think about all the moderate activities that you did in the last 7 days. Moderate activities refer to activities that take moderate physical effort and make you breathe somewhat harder than normal. Think only about those physical activities that you did for at least 10 minutes at a time.	
bm8 *(required)*	bm8:During the last 7 days, on how many days did you do moderate physical activities like heavy lifting, digging, aerobics, or fast bicycling?<br>*Includes activity at work, travel to and from places, and recreation. If the respondent does not know, enter 77.*	
Extended Individual Interview > Section 19: Behavioural measurements > Moderate physical activity<br>*Group relevant when: ${bm8} >0 and ${bm8} < 77*		
generated_note_name_499	bm9: How much time did you usually spend doing moderate physical activities on one of those days?<br>*You have to enter hours and minutes. If the respondent does not know, enter 77 in hours and minutes.*	
bm9h *(required)*	hours<br>*If less than 1 hour, enter 0.*	
bm9m *(required)*	minutes<br>*If more than 60 minutes, convert to hours and minutes.*	
generated_note_name_504 *(required)*	You have to enter more than 0 hours and 0 minutes of moderate activity<br>*Question relevant when: ${modzero} =0 and ${bm8} >0 and ${bm8} <77*	
generated_note_name_505	READ:<br><br>Think about the time you spent walking in the last 7 days. This includes at work and at home, walking to travel from place to place, and any other walking that you have done solely for recreation, sport, exercise, or leisure	
bm10 *(required)*	bm10: During the last 7 days, on how many days did you walk for at least 10 minutes at a time?<br>*Includes acticity at work, travel to and from places, and recreation. If the respondent does not know, enter 77.*	
Extended Individual Interview > Section 19: Behavioural measurements > Walking<br>*Group relevant when: ${bm10} >0 and ${bm10} <77*		
generated_note_name_508	bm11: How much time did you usually spend walking on one of those days?<br>*You have to enter hours and minutes. If the respondent does not know, enter 77 in hours and minutes.*	
bm11h *(required)*	hours<br>*If less than 1 hour, enter 0.*	
bm11m *(required)*	minutes<br>*If more than 60 minutes, convert to hours and minutes.*	
generated_note_name_513 *(required)*	You have to enter more than 0 hours and 0 minutes of walking<br>*Question relevant when: ${walkzero} =0 and ${bm10} >0 and ${bm10} < 77*	
generated_note_name_514	READ:<br><br>The last question is about the time you spent sitting on weekdays during the last 7 days. Include time spent at work, at home, while doing course work and during leisure time. This may include time spent sitting at a desk, visiting friends, reading, or sitting or lying down to watch television.	
Extended Individual Interview > Section 19: Behavioural measurements > Sedentary behaviour		
generated_note_name_516	bm12: How much time do you usually spend sitting or reclining on a typical day?<br>*You have to enter hours and minutes. If the respondent does not know, enter 77 in hours and minutes.*	
bm12h *(required)*	hours<br>*If less than 1 hour, enter 0.*	
bm12m *(required)*	minutes<br>*If more than 60 minutes, convert to hours and minutes.*	
generated_note_name_521 *(required)*	You have to enter more than 0 hours and 0 minutes of sitting<br>*Question relevant when: ${sitzero} =0 and ${bm12h} <77 and ${bm12m} <77*	
bm18 *(required)*	bm18: Have you consumed any alcohol within the past 12 months?	1 Yes<br>2 No<br>77 Don't know<br>88 Refused
bm20 *(required)*	bm20: During the past 12 months, how frequently have you had at least one standard alcoholic drink? Was it daily, 5-6 days per week, 3-4 days per week, 1-2 days per week, 1-3 days per month, less than once a month or never?<br>*Question relevant when: ${bm18} ='1'*	1 Daily<br>2 5-6 days per week<br>3 3-4 days per week

Field	Question	Answer
		4 1-2 days per week<br>5 1-3 days per month<br>6 Less than once a month<br>7 Never<br>88 Refused
Extended Individual Interview > Section 20: General anxiety and depression<br>*Group relevant when: ${ext_consent} ='1' and ${ps4n} =1*		
generated_note_name_529	READ<br><br>Now, I would like to ask you how you were feeling over the past two weeks.<br><br>Over the last 2 weeks, how often have you been bothered by the following problems? Not at all, on several days, on more than half the days or nearly every day?	
ga1 *(required)*	ga1: Feeling nervous, anxious or on edge	1 Never or one day (no days or 1 day)<br>2 Several days (2 to 6 days)<br>3 More than half the days (7 to 11 days)<br>4 Nearly every day (12 to 14 days<br>88 Refused
ga2 *(required)*	ga2: Not being able to stop or control worrying	1 Never or one day (no days or 1 day)<br>2 Several days (2 to 6 days)<br>3 More than half the days (7 to 11 days)<br>4 Nearly every day (12 to 14 days<br>88 Refused
ga3 *(required)*	ga3: Little interest or pleasure in doing things?	1 Never or one day (no days or 1 day)<br>2 Several days (2 to 6 days)<br>3 More than half the days (7 to 11 days)<br>4 Nearly every day (12 to 14 days<br>88 Refused
ga4 *(required)*	ga4: Feeling down, depressed, or hopeless?	1 Never or one day (no days or 1 day)<br>2 Several days (2 to 6 days)<br>3 More than half the days (7 to 11 days)<br>4 Nearly every day (12 to 14 days<br>88 Refused
ga5 *(required)*	ga5: Trouble falling or staying asleep, or sleeping too much?	1 Never or one day (no days or 1 day)<br>2 Several days (2 to 6 days)<br>3 More than half the days (7 to 11 days)<br>4 Nearly every day (12 to 14 days<br>88 Refused
ga6 *(required)*	ga6: Feeling tired or having little energy?	1 Never or one day (no days or 1 day)<br>2 Several days (2 to 6 days)<br>3 More than half the days (7 to 11 days)<br>4 Nearly every day (12 to 14 days<br>88 Refused
ga7 *(required)*	ga7: Poor appetite or overeating?	1 Never or one day (no days or 1 day)<br>2 Several days (2 to 6 days)<br>3 More than half the days (7 to 11 days)<br>4 Nearly every day (12 to 14 days

Field	Question	Answer
		88 Refused
ga8 *(required)*	ga8: Feeling bad about yourself — or that you are a failure or have let yourself or your family down?	1 Never or one day (no days or 1 day)<br>2 Several days (2 to 6 days)<br>3 More than half the days (7 to 11 days)<br>4 Nearly every day (12 to 14 days<br>88 Refused
ga9 *(required)*	ga9: Trouble concentrating on things, such as reading the newspaper or watching television?	1 Never or one day (no days or 1 day)<br>2 Several days (2 to 6 days)<br>3 More than half the days (7 to 11 days)<br>4 Nearly every day (12 to 14 days<br>88 Refused
ga10 *(required)*	ga10: Moving or speaking so slowly that other people could have noticed? Or so fidgety or restless that you have been moving a lot more than usual?	1 Never or one day (no days or 1 day)<br>2 Several days (2 to 6 days)<br>3 More than half the days (7 to 11 days)<br>4 Nearly every day (12 to 14 days<br>88 Refused
ga11 *(required)*	ga11: Thoughts that you would be better off dead, or thoughts of hurting yourself in some way?	1 Never or one day (no days or 1 day)<br>2 Several days (2 to 6 days)<br>3 More than half the days (7 to 11 days)<br>4 Nearly every day (12 to 14 days<br>88 Refused
Extended Individual Interview > Section 22: HIV status<br>*Group relevant when: ${ext_consent} ='1' and ${ps4n} =1*		
hiv1 *(required)*	READ:<br><br>Now, we have reached the final part of the interview.<br><br>hiv1: Have you ever been tested for HIV?	1 Yes<br>2 No<br>77 Don't know<br>88 Refused
hiv2 *(required)*	hiv2: What was the result of the HIV test?<br>*Question relevant when: ${hiv1} ='1'*	1 HIV positive<br>2 HIV negative<br>3 Do not want to disclose
Extended Individual Interview > Section 22: HIV status > Last HIV test<br>*Group relevant when: ${hiv2} ='2'*		
generated_note_name_548	hiv3: When was the last time you were tested?<br>*Enter only one of the following: years or months. Enter 77 in months if the respondent does not know and 88 if the respondent refused to anwer.*	
hiv3y	years ago	
hiv3m	months ago<br>*Question relevant when: ${hiv3y} !=''*	
hiv4 *(required)*	hiv4: Would you like to participate in an HIV test?<br>*Question relevant when: ${hiv1} ='2' or (${hiv1} ='1' and ${hiv2} ='2' and (${hiv3y} >0 or ${hiv3m} >=6))*	1 Yes<br>2 No
hiv6 *(required)*	hiv6: Test result<br>*Question relevant when: ${hiv4} ='1'*	1 HIV positive<br>2 HIV negative
generated_note_name_559	READ:<br><br>Thank you so much for taking the time to participate in this interview. This is very valuable and with your help the provision of health services for high blood sugar and blood pressure can be improved.	
